# A Randomized Controlled Trial of Teat-Sealant and Antibiotic Dry-Cow Treatments for Mastitis Prevention Shows Similar Effect on the Healthy Milk Microbiome

**DOI:** 10.3389/fvets.2020.00581

**Published:** 2020-09-02

**Authors:** Filippo Biscarini, Paola Cremonesi, Bianca Castiglioni, Alessandra Stella, Valerio Bronzo, Clara Locatelli, Paolo Moroni

**Affiliations:** ^1^Institute of Biology and Biotechnology in Agriculture, National Research Council (CNR), Milan, Italy; ^2^Università degli Studi di Milano, Dipartimento di Medicina Veterinaria, via dell'Università 6, Lodi, Italy; ^3^Quality Milk Production Services, Animal Health Diagnostic Center, Cornell University, Ithaca, NY, United States

**Keywords:** dairy cows, prophylaxis, selective dry-cow therapy, antibiotics, milk microbiome, teat sealant, cephalonium, cloxacillin

## Abstract

Lactating cows are routinely treated at dry-off with antibiotic infusions in each quarter for the cure and prevention of pathogenic intramammary infection, which remains the most common disease in dairy herds. This approach is known as blanket dry-cow therapy, usually effective for the prevention and cure of infections, but has been shown to potentially contribute to the emergence and spreading of antibiotic resistant bacterial strains. Exploring the use of non-antibiotic treatments coupled with selective dry-cow therapy is necessary to reduce the risk of antibiotic resistance and potential interference with milk microbiome balance. The impact of selective dry-cow therapy on the physiological milk microbiome needs to be carefully evaluated. In this small-scale trial, five healthy (no mastits, SCC <200,000 cells mL^−1^) second-parity cows from dry-off to 5 days after calving were sampled. For every cow, each quarter received a different treatment: (i) bismuth salnitrate (internal teat sealant, OrbSeal®, Zoetis, Italy), front right quarter; (ii) cephalonium dihydrate (Cepravin®, MSD, Italy), rear right quarter; (iii) benzathine cloxacillin (Cloxalene dry, Ati, Italy), rear left quarter. No treatment was applied to the remaining quarter (front left) which served as experimental control. For 16S rRNA gene sequencing, bacterial DNA was extracted from 5 ml of milk samples, amplified using the primers for the V3–V4 hypervariable regions and sequenced in one MiSeq (Illumina) run with 2 × 250-base paired-end reads. Bacteriological results confirmed that the quarters were all healthy. The phyla *Proteobacteria, Firmicutes*, and *Actinobacteria* were the most abundant for all treatments and controls at all three timepoints, accounting for over 80% of the entire milk microbiota composition. No significant differences were found between treatments and controls in terms of the major alpha and beta diversity indexes, revealing that antibiotic, and non-antibiotic treatments for selective dry-cow therapy did not alter significantly the milk microbiome of dairy cows. The milk microbiota composition showed a clear evolution over the lactation cycle, and the overall changes in the milk microbiota diversity over the lactation cycle were mainly independent of treatments.

## 1. Introduction

Intramammary infections (IMI) are still the disease class with the largest prevalence in dairy cattle farms worldwide [e.g., 24.8% of cows reported to be affected in the USA in 2013; (1)]. Given the high prevalence and the considerable estimated cost per case [$325–426; (2)], it has a substantial impact on the profitability of dairy farms. The main underlying pathogens involved in the aetiology of bovine mastitis include Gram-negative (e.g., *Escherichia coli*) and Gram-positive (e.g., *Staphylococcus aureus*) bacteria (3). Consequently, antibiotics have historically had a major role in the treatment of clinical and subclinical forms of mastitis in dairy cattle (4). The different means for therapy and prevention of IMI are implemented in mastitis control programmes that are adopted on a large scale by commercial dairy farms. The most common mastitis control protocols include blanket dry-cow therapy (BDCT), which relies on the antibiotic treatment of every cow during the dry period, and selective dry-cow therapy (SDCT), which targets those animals and specific mammary quarters that are infected and need to be treated (5, 6). The dry period is a critical component of the milk production cycle for two main reasons: (i) high cure rates for IMI can be achieved (7, 8), and (ii) the rate of new IMI is greater in the periparturient period than at any other point during lactation (9). Growing concerns and evidence on the development of antibiotic-resistant bacterial strains and their spread to other livestock species and humans, with potential zoonotic risks, are pushing the investigation and adoption of alternative strategies (10–12). Non-antibiotic solutions include probiotics, bacteriocins, bacteriophages, teat sealants, lactoferrin, herbal compounds, and vaccinations (4, 13–15). For dairy herds with a low prevalence of contagious mastitis and a consistently low somatic cell count (SCC), SDCT is a preferable alternative approach to mastitis control. Internal teat sealants (ITS) are a class of non-antimicrobial products that has proven to be just as efficacious as dry-cow therapy (DCT) in the prevention of IMI during the dry period. ITS may provide just a physical barrier or also inhibit bacterial growth (16). The use of an ITS in a SDCT program ensures that all healthy quarters have some form of protection against dry-period IMI. Studies have found that SDCT is better than BDCT in the prevention and treatment of IMI during the dry period and can reduce the use of antimicrobials by 21% (6, 17, 18).

Evidence has been accumulating on the role of the udder microbiomes (teat canal and milk) on the mammary health: their dysbiosis has been hypothesized as a predisposing factor for mastitis (19), in line with recent views that challenge Koch's “one microbe–one disease” paradigm in favor of the more complex concept of the pathobiome as etiologic agent (20). Mastitic quarters have been found to show higher bacterial load and lower diversity compared to healthy quarters (21–23). Previous works on the effect of mastitis treatments on the teat-canal and milk microbiomes involved mastitic cows treated with antibiotics or healthy cows under DCT with antibiotics and teat sealant. Results showed that the udder microbiomes change with infection and over time but appear to be resilient to therapeutic and prophylactic antimicrobial treatments (23). Derakhshani et al. (24) assessed the use of a penicillin-novobiocin formulation together with teat sealant; Bonsaglia et al. (25) evaluated the effect of a third-generation cephalosporin (ceftiofur) combined with teat sealant, and of teat sealant alone, on the milk microbiome. It remains to be determined whether or not other classes of antimicrobials may have a long-lasting effect on the composition of the udder microbiome as a whole and, specifically, of the milk microbiome.

Considering that 3rd and 4th generation cephalosporins are currently not recommended for veterinary use according to EU guidelines (26), it is important to evaluate other types of antimicrobials used in DCT and their effect on the bovine milk microbiome, relative to antibiotic-less prophylactic strategies and untreated controls. In this small-scale trial, we sampled healthy cows under DCT and implemented a within-subject experimental design based on udder quarters: each quarter received a different treatment: cephalonium dihydrate (first-generation cephalosporin), benzathine cloxacillin, and bismuth subnitrate (internal teat sealant); the last quarter was left untreated and served as experimental control. We hypothesize that antibiotic and non-antibiotic treatments for SDCT do not alter significantly the milk microbiome of healthy dairy cows: this would further support the replacement of antibiotics with teat-sealant for SDCT. We followed the microbiome research terminology proposed by Marchesi and Ravel (27).

## 2. Materials and Methods

### 2.1. Ethics Statements

This study was conducted on a single commercial dairy farm in Romano di Lombardia (Bergamo, Italy), thanks to its long-standing relationship with the University of Milan. The study was reviewed and approved by the Ethics Committee for Animal Welfare of the University of Milan (authorization n. 88/2019).

### 2.2. Animals, Treatments, and Sampling Time

Five Holstein-Friesian cows were selected for this study from a 140 lactating-cows dairy farm in Northern Italy, with 1 year average bulk tank somatic cell count (SCC) of 159· 10^3^ cells mL^−1^ and herd milk production average of 37 L d^−1^. These were all second-parity cows without any symptoms of clinical mastitis and SCC < 200,000 cells mL^−1^ per lactation based on DHIA data (Dairy Herd Information Association), as per the study inclusion criteria. Cows had freestall housing with cubicles bedded with pelleted straw for lactating animals and straw during the dry period (duration in the range 54–62 days). The herd was also prescreened using bulk tank culture to determine whether cows were confirmed negative for *Mycoplasma* spp. The animals were followed over a period of 12 weeks, and sampled at three time points: dry-off, calving (colostrum) and 5 days in milk (5 DIM). Drying-off was abrupt. The animals remained healthy for the entire sampling period, without signs of clinical mastitis. During the experimental period, cows were fed ad libitum with a silage-free mixed ration using alfalfa hay, straw, and supplemented minerals and vitamins. After parturition, cows were milked twice a day (3 a.m., 3 p.m.) in a double-6 herringbone parlour.

During the dry-off period, in each cow three of the four quarters were treated with: (i) bismuth subnitrate (internal teat sealant, Orbeseal®, Zoetis, Italy), front right quarter; (ii) cephalonium dihydrate (Cepravin®, MSD, Italy), rear right quarter; and (iii) benzathine cloxacillin (Cloxalene dry, Ati, Italy), rear left quarter. No treatment was applied to the remaining quarter (front left) which served as experimental control. Cepravin is a first-generation semi-synthetic cephalosporin antibiotic (cephalonium dihydrate) with activity against aerobic Gram-positive and a few community-acquired Gram-negative bacteria. Cephalonium is used in veterinary medicine and has broad-spectrum activity. Cloxalene is benzathine cloxacillin, suited for dry-off and for the treatment of subclinical Gram-positive associated mastitis susceptible to cloxacillin [e.g., *S. aureus, Streptococcus agalactiae, Streptococcus dysgalactiae, Streptococcus uberis*, non-aureus *Staphylococci* (NAS), *Trueperella pyogenes*]. It is also used to prevent mammary infections that may arise during the dry period or around calving and early lactation. From each quarter milk samples were collected at dry-off (T1), the day of calving (T2, colostrum), and 5 DIM (T3): milk samples were collected before the afternoon milking. Sampling was carried out following the best practices for 16S rRNA-gene sequencing experiments (28). The sample size (5 cows, 4 quarters, 3 timepoints) was determined as a trade-off between ethics constraints (the fewer animals used, the better) and statistical power calculations (80% power to detect an effect of 0.41–0.44 standard deviations with 0.05 false positive -α- threshold). Milk microbiome studies of comparable size have been reported (24, 29, 30).

### 2.3. Milk Samples Procedures and Somatic Cell Count

Before milk sample collection, teat ends were carefully cleaned and disinfected with chlorhexidine and 70% alcohol in accordance with National Mastitis Council (NMC, 2017) recommendations for aseptic collection of milk samples. First streams of foremilk were discharged, and then approximately 10 mL of milk was collected with a sterile technique from each teat into sterile vials. These vials were previously identified with herd, cow number, quarter, and date. Samples were transported at 4 °C to the laboratory and frozen at -20 °C until bacteriological assays and SCC tests were performed. The SCC was estimated on a per-quarter basis with an automated somatic cell counter (Bentley Somacount 150, Bentley Instrument, Chaska, MN). Milk samples were split in two aliquots, one for bacteriology and one for sequencing. The same procedure was performed at all timepoints.

### 2.4. Bacteriological Analysis

Bacteriological milk cultures were performed at the University of Milan following published procedures recognized by the NMC (2017). From each sample, 10 μL of milk were spread on blood agar plates (5% defibrinated sheep blood). Plates were incubated aerobically at 37 °C and examined after 24 and 48 h. Colonies were provisionally identified based on size, Gram stain, morphology, and hemolysis pattern. Representative colonies were then subcultured on blood agar plates and incubated again at 37 °C for 24 h to obtain pure cultures. Catalase-negative Gram-positive cocci were identified as *Streptococci* and species were differentiated by further biochemical tests (growth in 6.5% NaCl broth, esculin hydrolysis, fermentation of ribose, sorbitol, sucrose, and inulin). Coagulase tube test was used to differentiate catalase-positive gram-positive cocci as *S. aureus* or NAS. Gram-negative isolates were identified using colony morphology, Gram-staining characteristics, oxidase test, indol test, and inoculation in Simmons citrate (Laboratorios Conda, Madrid, Spain), motility indol ornithine, and biochemical reactions on MacConkey (Oxoid, Basingstoke, UK). Microorganisms other than bacteria were confirmed by microscopic appearance. Samples where three or more pathogens grew were considered contaminated.

### 2.5. 16S rRNA-Gene Sequencing

For each quarter, 5 mL of milk were centrifuged by using a DNA extraction method based on the combination of a chaotropic agent, guanidium thiocyanate, with silica particles, to obtain bacterial cell lysis and nuclease inactivation (31). DNA quality and quantity were assessed using a NanoDrop ND-1000 spectrophotometer (NanoDrop Technologies, Wilmington, DE, USA). The isolated DNA was stored at -20 °C until use. Bacterial DNA was amplified using the primers described in literature (32) which target the V3–V4 hypervariable regions of the 16S rRNA gene. All PCR amplifications were performed in 25 μL volume per sample. A total of 12.5 μL of Phusion High-Fidelity Master Mix 2x (ThermoFisher Scientific, Walthem, MA, USA) and 0.2 μL of each primer (100 μm) were added to 2 μL of genomic DNA (5 ng μL^−1^). Blank controls (i.e., no DNA template added to the reaction) were also performed. No DNA extraction negative controls have been run. A first amplification step was performed in an Applied Biosystem 2,700 thermal cycler (ThermoFisher Scientific). Samples were denatured at 95 °C for 3 min, followed by 25 cycles with a denaturing step at 98 °C for 30 s, annealing at 56 °C for 1 min and extension at 72 °C for 1 min, with a final extension at 72 °C for 7 min. Amplicons were cleaned with Agencourt AMPure XP (Beckman, Coulter Brea, CA, USA) and libraries were prepared following the 16S Metagenomic Sequencing Library Preparation Protocol (Illumina, San Diego, CA, USA). The libraries obtained were quantified by Real Time PCR with KAPA Library Quantification Kits (Kapa Biosystems, Inc., MA, USA), pooled in equimolar proportion and sequenced in one MiSeq (Illumina) run with 2 × 250-base paired-end reads. The 16S rRNA gene sequences obtained from this study were deposited in the EMBL-EBI European Nucleotide Archive (ENA) repository with the accession number PRJEB38332.

### 2.6. Bioinformatics Processing

Demultiplexed paired-end reads from 16S rRNA-gene sequencing were first checked for quality using FastQC (33) for an initial assessment. Forward and reverse paired-end reads were joined into single reads using the C++ program SeqPrep (34). After joining, reads were filtered for quality based on: (i) maximum three consecutive low-quality base calls (Phred < 19) allowed; (ii) fraction of consecutive high-quality base calls (Phred > 19) in a read over total read length ≥ 0.75; (iii) no “N” -labeled bases (missing/uncalled) allowed. Reads that did not match all the above criteria were excluded. All remaining reads were combined in a single FASTA file for the identification and quantification of OTUs (operational taxonomic units). Reads were aligned against the Greengenes closed reference sequence collection release 13.8, with 97% cluster identity (35), applying the CD-HIT clustering algorithm (36). A predefined taxonomy map of reference sequences to taxonomies was then used for taxonomic identification along the main taxa ranks down to the genus level (domain, phylum, class, order, family, genus). By counting the abundance of each OTU, the OTU table was created and then grouped at each phylogenetic level. Records belonging to OTUs with total counts lower than 10 in fewer than 2 samples were filtered out. All of the above steps, except the FastQC reads quality check, were performed with the QIIME 1.9 open-source bioinformatics pipeline for microbiome analysis (37). The command lines and parameters used to process 16S rRNA-gene sequence data are detailed in Biscarini et al. (38).

### 2.7. Alpha and Beta Diversity

The milk microbial diversity was assessed within- (alpha diversity) and across- (beta diversity) samples. All indices (alpha and beta diversity) were estimated from the complete OTU table (at the OTU level), filtered for OTUs with more than 10 total counts distributed in at least two samples. Besides the number of observed OTUs directly counted from the OTU table, within-sample microbial richness and diversity were estimated using the following indices: Chao1 and ACE (Abundance-based coverage Estimator) for richness, Shannon, Simpson, and Fisher's alpha for diversity (39–44), Simpson E and Pielou's J (Shannon's evenness) for evenness (45). The across-sample milk microbiota diversity was quantified by calculating Bray-Curtis dissimilarities (46). Prior to the calculation of the Bray-Curtis dissimilarities, OTU counts were normalized for uneven sequencing depth by cumulative sum scaling [CSS; (47)]. Among groups (teat sealant, cephalonium, cloxacillin, and control) and pairwise Bray-Curtis dissimilarities were evaluated non-parametrically using the permutational analysis of variance approach [999 permutations; (48)]. Details on the calculation of the mentioned alpha- and beta-diversity indices can be found in (38) (S2 Appendix).

### 2.8. Statistical Analysis

As a consequence of the chosen experimental design, data were hierarchically structured with treatments nested within individuals, and measurements repeated over time. Therefore, observations could not be assumed to be independent from each other, but were correlated within individual cows. This was taken into account in the linear models used to analyse between-group (treatments, timepoints) differences in terms of SCC, alpha diversity indices and OTU counts; SCC data were not normally distributed and have been log-transformed prior to the analysis. The model had the following form:

(1) $$\begin{array}{l} y_{ijk}=\mu +cow_{j}+{\left[treatment|timepoint\right]}_{k\left(j\right)}+e_{ijk} \end{array}$$

where *y*_*ijk*_ is the *log*(*SCC*), alpha diversity index value or OTU counts for record *i* from cow *j* with treatment or timepoint *k*; μ is the intercept, *cow*_*j*_ is the systematic effect of the individual cow, [*treatment*|*timepoint*]_*k*(*j*)_ is the effect of treatment or timepoint *k* nested within cow *j* and *e*_*ijk*_ is the residual. $Var\left(y\right)=\Sigma +\text{I}\sigma_{e}^{2}$, where **Σ** is a block diagonal matrix, with 1s on the diagonal and the covariances **σ**_*ij*_ between records within cows in the off-diagonal block elements; **I** is the identity matrix and $\sigma_{e}^{2}$ is the residual variance. To test the interaction between treatments and timepoints, model 1 was expanded as follows:

(2) $$\begin{array}{l} y_{ijkz}=\mu +cow_{j}+treatment_{k\left(j\right)}+timepoint_{z\left(j\right)}\\ \begin{array}{l} \;+{\left(\text{treatment x timepoint}\right)}_{kz\left(j\right)}+e_{ijkz} \end{array} \end{array}$$

where terms were as in model 1 with the addition of the interaction terms (treatment x timepoint)_*kz*(*j*)_, again nested within individual cows. Besides correctly accounting for not independent nested observations, multilevel models as those in Equations (1) and (2) have the property of increasing the power of analysis through lower between-subject variability (each subject is its own control, fewer degrees of freedom).

### 2.9. Software

Reads from 16S rRNA-gene sequencing were processed with the QIIME pipeline v. 1.9 (37), used also to estimate most diversity indices. The ACE index and sample-base rarefaction were estimated using own *Python* (https://github.com/filippob/Rare-OTUs-ACE.git) and *R* (https://github.com/filippob/sampleBasedRarefaction) scripts. Plots were generated using the ggplot2 *R* package (49). Additional data handling and analysis was performed with the *R* environment for statistical computing (50).

## 3. Results

### 3.1. SCC and Culture-Based Bacteriology

At the onset of the experiment the median quarter SCC per group was in the range 43,000 (cephalonium)–139,000 (cloxacillin) cells mL^−1^. At the end of the experiment, SCC increased in the control group (+41.0%), and decreased with the teat sealant (−22.1%), cephalonium (−53.5%), and cloxacillin (−66.9%) treatments (Table 1). These differences were however not statistically significant. A physiological marked SCC increment was observed at calving (colostrum) across all groups (up to 2,000,000 cells mL^−1^). Results from culture-based bacteriology showed that the milk samples used in this study were all negative to culture. No differences have been observed along the sampling period and among the quarters with different treatments.

**Table 1:** Median somatic cell count (cells mL^−1^) per treatment and time-point.

	**Timepoint**			
**Treatment**	**N (x time)**	**Dry-off**	**Calving**	**Early milk**
Cephalonium	5	4.30 × 10^4^	1.38 × 10^6^	2.00 × 10^4^
Cloxacillin	5	1.39 × 10^5^	1.39 × 10^6^	4.60 × 10^4^
Teat sealant	5	9.50 × 10^4^	2.03 × 10^6^	7.40 × 10^4^
Control	5	1.34 × 10^5^	1.56 × 10^6^	1.89 × 10^5^

### 3.2. Sequencing Metrics

Sequencing the V3–V4 regions of the bacterial 16S rRNA-gene produced a total of 10,707,392 reads (joined R1-R2 paired-end reads), with an average of 178,456 reads per sample (5 cows × 4 quarters × 3 time-points = 60 samples). After quality filtering, 2,543,623 sequences were removed, leaving 8,163,769 sequences for subsequent analyses (76% average retention rate, maximum 85%, minimum 61%). The average number of sequences per treatment and time-point is reported in Table S1: this varies from a minimum of 93,474 (± 23,020) in the cephalonium group at dry-off to a maximum of 176,831 (± 122,987) in the cephalonium group at calving. The initial number of OTUs identified was 11603; after filtering out OTUs with less than 10 counts in at least 2 samples, 4,495 distinct OTUs were left. To check whether sequencing depth and sample size were adequate to characterize the composition of the bovine milk microbiota, sequence-based and sample-based rarefaction curves were generated from the OTU table before filtering (11,603 OTUs). Sequence-based rarefaction curves were obtained from the QIIME pipeline; the sample-based rarefaction curve was produced with *ad hoc R* functions. The observed number of OTUs detected was plotted as a function of the number of reads (up to 40,922) in each sample and of the number of samples (Figure S1). Both curves tend to plateau asymptotically toward a maximum, indicating that sequencing depth and the number of samples were adequate to characterize the milk microbiota in the present study. Deeper sequencing or the addition of any other samples would likely not increase significantly the number of new OTUs discovered.

### 3.3. Core Milk Microbiome

**Figure 1:**
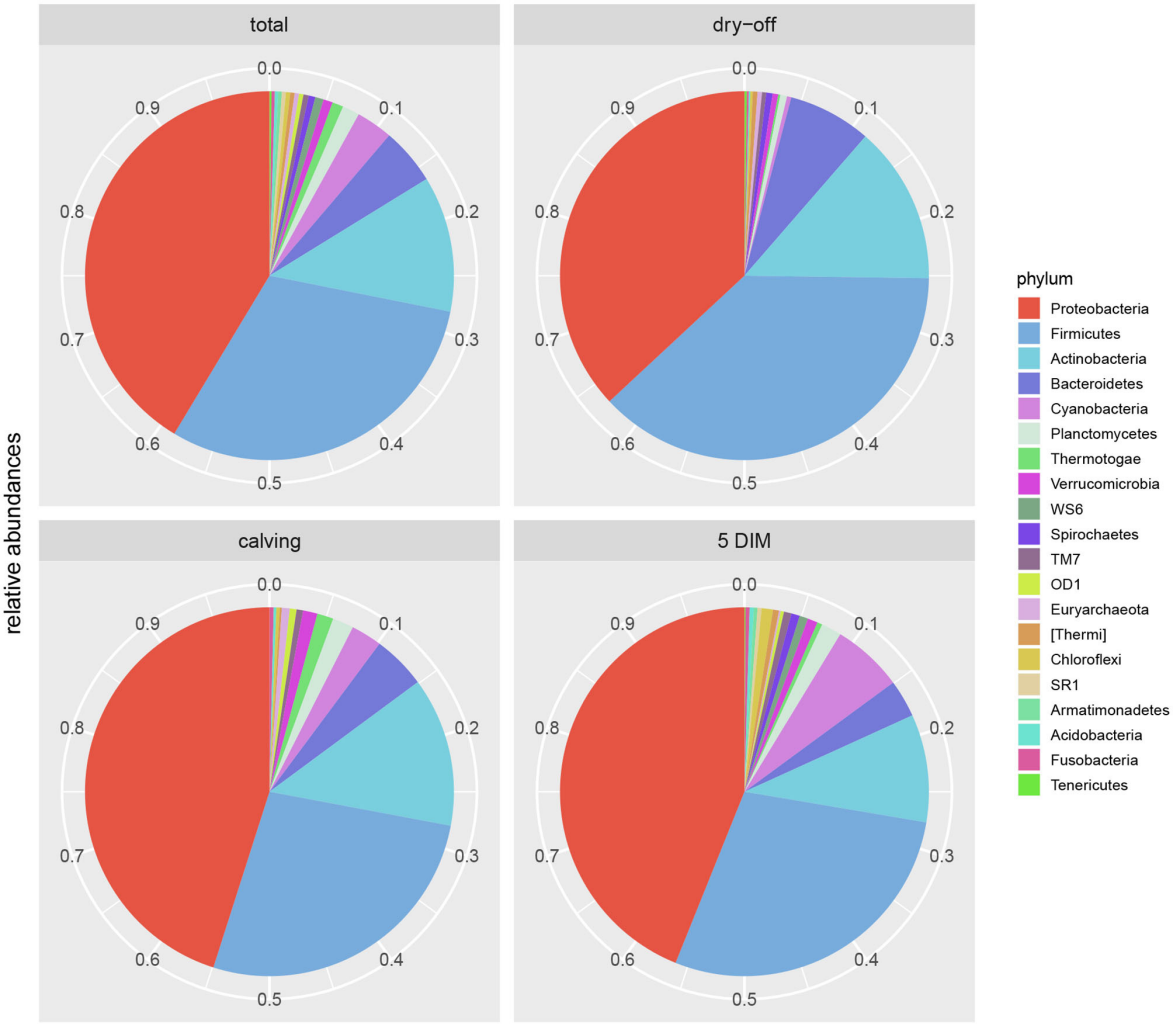
Pie-charts of phylum relative abundances in the dairy cow milk microbiome over time. All data, and time point breakdown.

Results from culture-based bacteriology confirmed that there were no milk samples either patently contaminated or from infected quarters. Therefore, results from 16S rRNA-gene sequencing from all samples could be used to characterize the core milk microbiome in dairy cows. Nevertheless, we can not positively exclude that a fraction of the bacterial taxa detected from 16S rRNA-gene sequencing at very low abundances in our milk samples could be the result of sporadic contamination. OTUs were grouped taxonomically from the phylum to genus level (phylum, class, order, family, genus). The 4,495 OTUs with more than 10 counts across samples clustered into 23 distinct phyla, 51 classes, 95 orders, 221 families, and 542 genera. Taxa with relative abundance < 0.1% were not considered. Considering OTUs shared by 99% of the samples, the dairy cow core milk microbiota comprised only a small portion of the total detected OTUs (Table 2), restricted to three phyla (*Proteobacteria, Firmicutes, Actinobacteria*). The core milk microbiome featured the genera *Corynebacterium, Propionibacterium, Staphylococcus, Lactobacillus, Lactococcus, Streptococcus, Bradyrhizobium, Achromobacter, Enhydrobacter* with a relative majority of the families *Pseudomonadaceae, Alcaligenaceae*, and *Streptococcaceae*. In terms of relative abundances, Figure 1 reveals that most of the reads belonged to the phyla *Proteobacteria, Firmicutes*, and *Actinobacteria*, which accounted for over 80% of the entire milk microbiota. A complete list of the bacterial groups at phylum, family, and genus level as well as their relative abundances is reported in Table S2.

**Table 2:** Distinct OTUs included in the dairy cow core milk microbiome (100% of the samples).

**Phylum**	**Class**	**Order**	**Family**	**Genus**	**Avg counts**
*Actinobacteria*	*Actinobacteria*	*Actinomycetales*	*Corynebacteriaceae*	*Corynebacterium*	347.45
*Actinobacteria*	*Actinobacteria*	*Actinomycetales*	*Propionibacteriaceae*	*Propionibacterium*	1201.24
*Firmicutes*	*Bacilli*	*Bacillales*	*Staphylococcaceae*	*Staphylococcus*	166.46
*Firmicutes*	*Bacilli*	*Lactobacillales*	*Lactobacillaceae*	*Lactobacillus*	277.52
*Firmicutes*	*Bacilli*	*Lactobacillales*	*Streptococcaceae*	*Lactococcus*	28.40
*Firmicutes*	*Bacilli*	*Lactobacillales*	*Streptococcaceae*	*Streptococcus*	1901.83
*Proteobacteria*	*Alphaproteobacteria*	*Rhizobiales*	*Bradyrhizobiaceae*	*Bradyrhizobium*	621.75
*Proteobacteria*	*Betaproteobacteria*	*Burkholderiales*	*Alcaligenaceae*	*Achromobacter*	46.87
*Proteobacteria*	*Gammaproteobacteria*	*Pseudomonadales*	*Moraxellaceae*	*Enhydrobacter*	1373.12
*Proteobacteria*	*Gammaproteobacteria*	*Pseudomonadales*	*Pseudomonadaceae*	*Pseudomonas*	3280.24
*Proteobacteria*	*Betaproteobacteria*	*Burkholderiales*	*Alcaligenaceae*		2702.20
*Proteobacteria*	*Gammaproteobacteria*	*Pseudomonadales*	*Pseudomonadaceae*		220.06
*Proteobacteria*	*Betaproteobacteria*	*Burkholderiales*			10240.48

### 3.4. Development of the Milk Microbiome Over Time (Dry-Off, Calving, 5 DIM)

**Figure 2:**
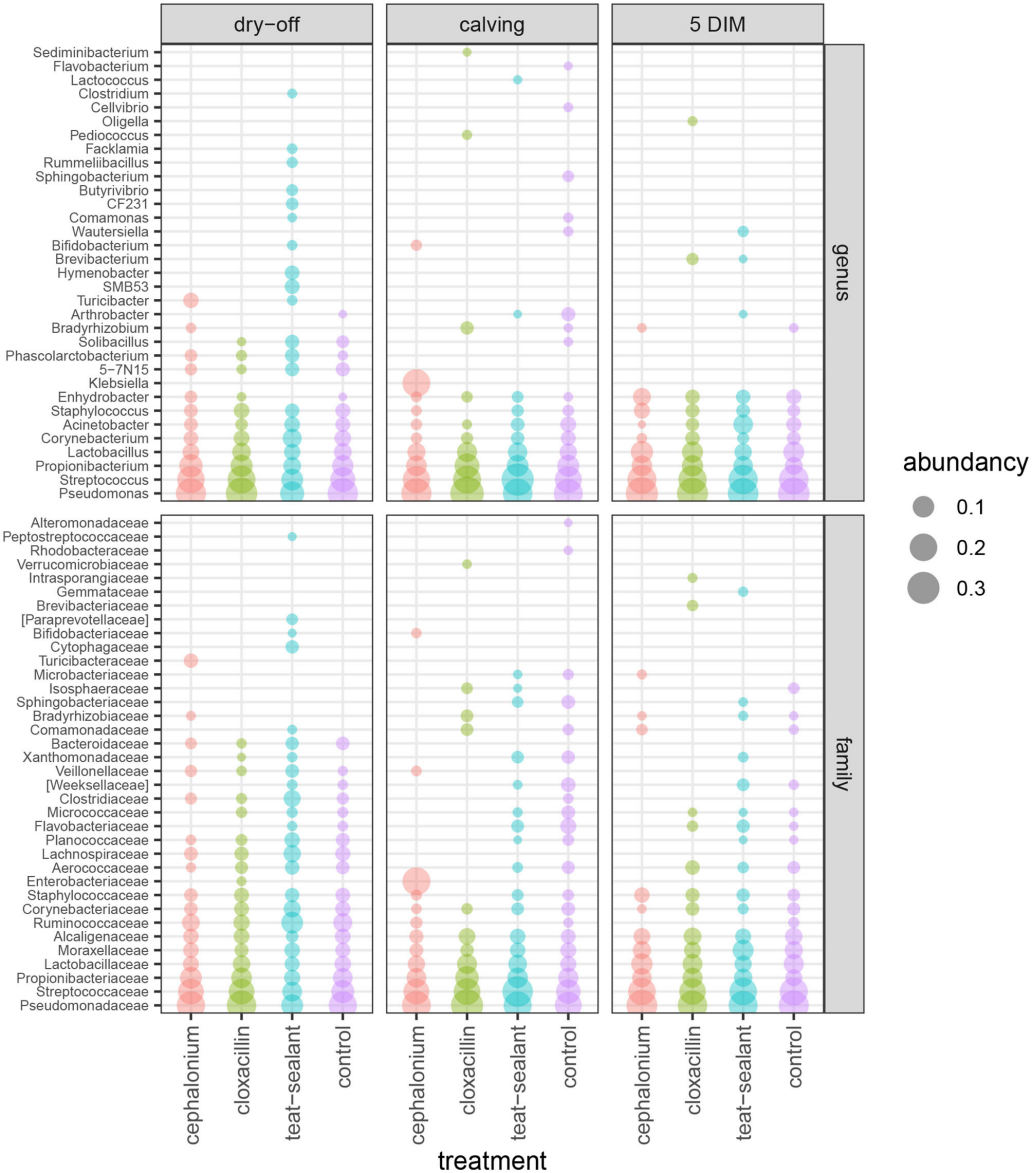
Bubble chart of relative abundances for the families and genera identified in the bovine milk microbiome from 60 samples taken from differentially treated quarters at three timepoints (*n* = 5 lactating cows). Only taxa with relative abundance ≥ 1% are shown.

**Figure 3:**
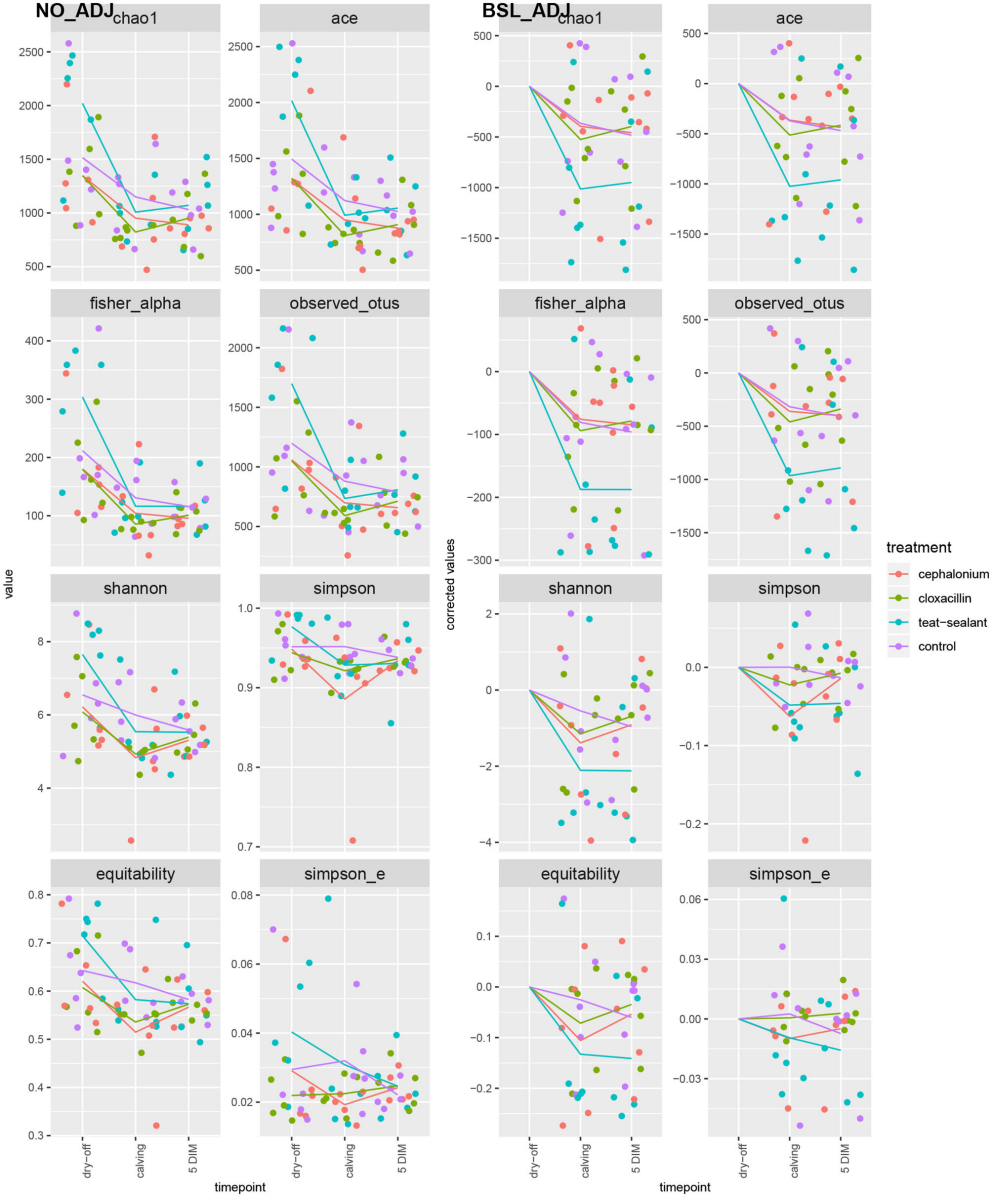
Alpha diversity indices over time and per treatment. Raw values on the left pane (NO_ADJ), values adjusted for baseline on the right pane (BSL_ADJ). Solid lines are the average values per treatment and timepoint.

**Figure 4:**
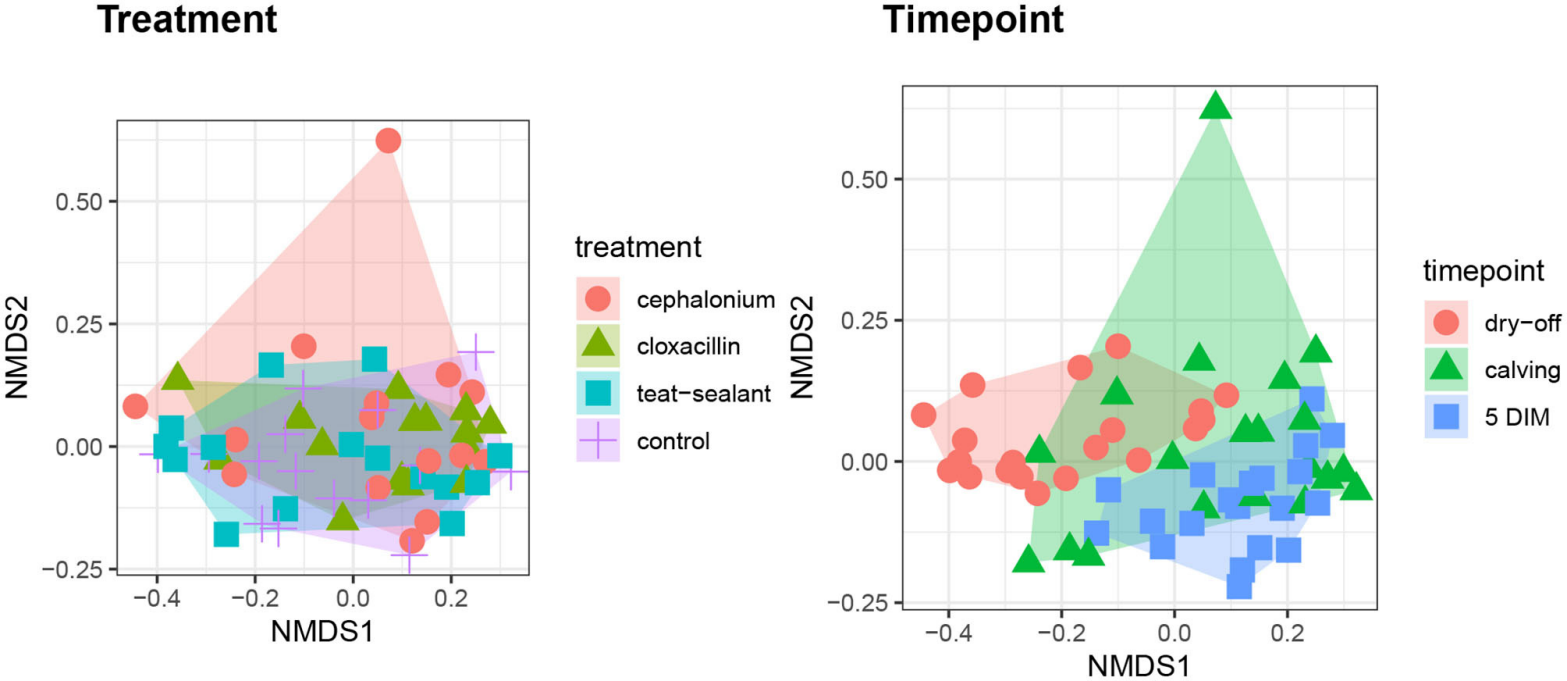
First two dimensions from the (non-metric) multi-dimensional scaling of the Bray-Curtis dissimilarity matrix. Samples were grouped by experimental units: by treatment on the left pane, by timepoint on the right pane. PERMANOVA among treatments p-value = 0.157, PERMANOVA among timepoints p-value = 0.001 (using 999 permutations).

Figures 1, 2 show the relative abundance of phyla and genera in the milk microbiome, overall and over time (dry-off, calving, 5 DIM). *Firmicutes* were found to be the most abundant phylum in the milk microbiome during dry-off, with *Proteobacteria* running up (39.3 and 36.7%, respectively), while at calving and 5 DIM milk sampling this was reverted, with *Proteobacteria* (47.9 and 46.2%) more abundant than *Firmicutes* (28 and 29.2%). The third and fourth most abundant phyla were *Actinobacteria* (13.9, 13.5, 10.8%) and *Bacteroidetes* (7.9, 5.5, 4.2%), at all timepoints. This has consequences on the *Firmicutes* to *Bacteroidetes* ratio (F:B), which is lower at dry-off (10.6) and higher at later time points (18.3, 22.3). Table 3 reports the 61 OTUs, at the various taxonomic levels, which are significantly differentially abundant over time. The top significantly different OTUs are the genera *rc4-4, Saliniccocus, Dorea, Ruminococcus*, and *YRC22*, the families *Peptococcaceae* and *RF16*, the orders *RF39* and *Chlorophyta*, the phylum *Tenericutes*. In all cases, the largest difference in counts was observed at dry-off vs calving and 5 DIM. Indexes of richness (observed number of OTUs, Chao1, ACE), evenness (Simpson E, equitability -a.k.a. Shannon's evenness) and combinations thereof (Shannon's and Simpson's diversity indices, Fisher's alpha) describe the diversity of the milk microbiota. Results per timepoint are reported in Figure 3 and Table 4: all comparisons between time points, except for Simpson E, were statistically significant. Figure 4 shows the first two dimensions from the (non-metric) multi-dimensional scaling of the Bray-Curtis dissimilarity matrix.

**Table 3:** OTUs with significant differential abundance between time points (alpha ≤ 0.05).

**Taxon**	**OTU**	**Dry-off**	**Calving**	**5 DIM**	***p*-value**
Phylum	Tenericutes	57.80	1.30	17.30	1.39e-03
Phylum	Cyanobacteria	270.50	2586.15	8457.60	2.56e-02
Class	Mollicutes	49.45	0.95	17.30	1.66e-03
Class	Clostridia	12480.30	3593.15	2885.95	9.03e-03
Class	Verruco-5	24.70	1.95	0.00	1.93e-02
Class	4C0d-2	119.00	25.40	0.05	2.01e-02
Class	Chloroplast	148.40	2506.65	8450.05	2.37e-02
Class	Bacteroidia	3646.05	755.60	756.25	3.14e-02
Order	RF39	15.40	0.75	2.50	5.37e-05
Order	Chlorophyta	1.10	545.95	1268.30	3.18e-04
order	Clostridiales	12454.70	3304.15	2732.80	9.55e-03
Order	Aeromonadales	325.10	17.20	32.25	1.75e-02
Order	Rhodospirillales	42.05	277.95	599.15	1.88e-02
Order	WCHB1-41	24.70	1.95	0.00	1.93e-02
Order	YS2	119.00	25.40	0.05	2.01e-02
Order	Bacteroidales	3646.05	755.60	756.25	3.14e-02
Order	Anaeroplasmatales	16.90	0.05	0.00	3.74e-02
Order	Streptophyta	107.45	1959.00	7178.40	3.83e-02
Family	Peptococcaceae	75.85	7.65	5.00	2.52e-05
Family	RF16	185.40	18.05	27.20	1.35e-03
Family	Clostridiaceae	1740.15	709.35	384.25	1.86e-03
Family	S24-7	189.95	40.20	3.80	4.12e-03
Family	Ruminococcaceae	4620.30	881.80	733.45	9.30e-03
Family	Lachnospiraceae	2374.00	327.95	450.35	9.47e-03
Family	Moraxellaceae	2245.30	2558.45	6109.85	1.36e-02
Family	Rhodospirillaceae	31.35	267.65	592.85	1.74e-02
Family	RFP12	24.70	1.95	0.00	1.93e-02
Family	p-2534-18B5	15.80	1.40	0.15	2.10e-02
Family	Carnobacteriaceae	160.35	95.05	353.90	2.13e-02
Family	Peptostreptococcaceae	460.85	91.25	144.20	2.17e-02
Family	Succinivibrionaceae	323.00	7.05	28.40	2.40e-02
Family	Rikenellaceae	349.95	61.75	85.25	3.15e-02
Family	Bacteroidaceae	1261.20	329.30	244.50	3.16e-02
Family	Anaeroplasmataceae	16.90	0.05	0.00	3.74e-02
Genus	rc4-4	70.10	6.90	0.05	6.46e-06
Genus	Salinicoccus	34.90	0.00	0.50	4.63e-05
Genus	Dorea	267.80	26.40	25.30	4.91e-05
Genus	Ruminococcus	275.20	50.10	21.95	7.89e-04
Genus	YRC22	82.90	8.50	3.05	7.93e-04
Genus	Succinivibrio	145.50	4.25	18.50	1.54e-03
Genus	Alloiococcus	78.75	10.10	7.20	2.30e-03
Genus	GW-34	26.20	0.00	0.80	2.66e-03
Genus	Mogibacterium	13.90	4.20	0.65	5.32e-03
Genus	[Clostridium]	121.20	22.10	37.10	7.77e-03
Genus	SMB53	768.40	336.95	183.80	8.80e-03
Genus	Limnohabitans	0.45	2.20	26.45	9.91e-03
Genus	Helcococcus	14.75	0.05	0.00	1.38e-02
genus	Polaromonas	0.00	2.30	29.70	1.45e-02
Genus	Epulopiscium	91.05	21.25	1.00	1.46e-02
Genus	Phascolarctobacterium	989.60	288.30	132.85	1.79e-02
Genus	Roseburia	51.90	3.10	15.80	2.16e-02
Genus	Butyrivibrio	448.50	108.40	160.55	2.24e-02
Genus	Clostridium	159.15	63.90	41.00	2.28e-02
Genus	Propionicimonas	38.55	1.00	0.20	2.98e-02
Genus	Coprococcus	153.25	28.35	40.10	3.03e-02
Genus	5-7N15	1084.85	225.20	220.15	3.16e-02
Genus	Anaerostipes	92.35	4.85	26.75	3.36e-02
Genus	Ruminobacter	165.80	0.50	5.15	4.32e-02
Genus	[Ruminococcus]	67.35	12.60	20.10	4.47e-02
Genus	Erythrobacter	5.90	27.90	2.65	4.63e-02
Genus	Rummeliibacillus	370.80	84.35	135.55	4.90e-02

*Taxonomic level, specific OTU, counts at dry-off, calving, and 5 DIM, p-value*.

**Table 4:** Alpha diversity indices by timepoint and treatment (*p*-value from linear mixed model).

**Indices**	**Dry-off**	**Calving**	**5 DIM**	**Cephal**.	**Cloxac**.	**t-sealant**	**Controls**	**Timepoint**	**Treatment**	**Treat:time**
Chao1	1557.57	982.90	985.66	1062.76	1040.18	1366.56	1232.00	5.51e-06	6.36e-02	4.18e-01
Fisher alpha	218.75	109.21	107.07	126.70	121.98	178.70	152.66	6.91e-07	7.06e-02	3.62e-01
Observed otus	1251.65	726.20	741.85	804.93	784.80	1080.73	955.80	5.85e-06	6.41e-02	3.10e-01
Shannon	6.62	5.32	5.45	5.45	5.47	6.24	6.04	2.94e-04	8.80e-02	5.67e-01
Simpson	0.96	0.92	0.93	0.92	0.93	0.95	0.95	3.23e-02	3.03e-01	4.55e-01
Equitability	0.65	0.56	0.57	0.57	0.57	0.62	0.61	2.33e-03	1.14e-01	5.46e-01
Simpson e	0.03	0.03	0.02	0.02	0.02	0.03	0.03	3.81e-01	3.33e-01	7.28e-01
ACE	1536.77	954.79	957.43	1034.17	1011.04	1348.66	1204.79	4.74e-06	5.43e-02	3.73e-01

### 3.5. Effect of Mastitis Treatments

**Figure 5:**
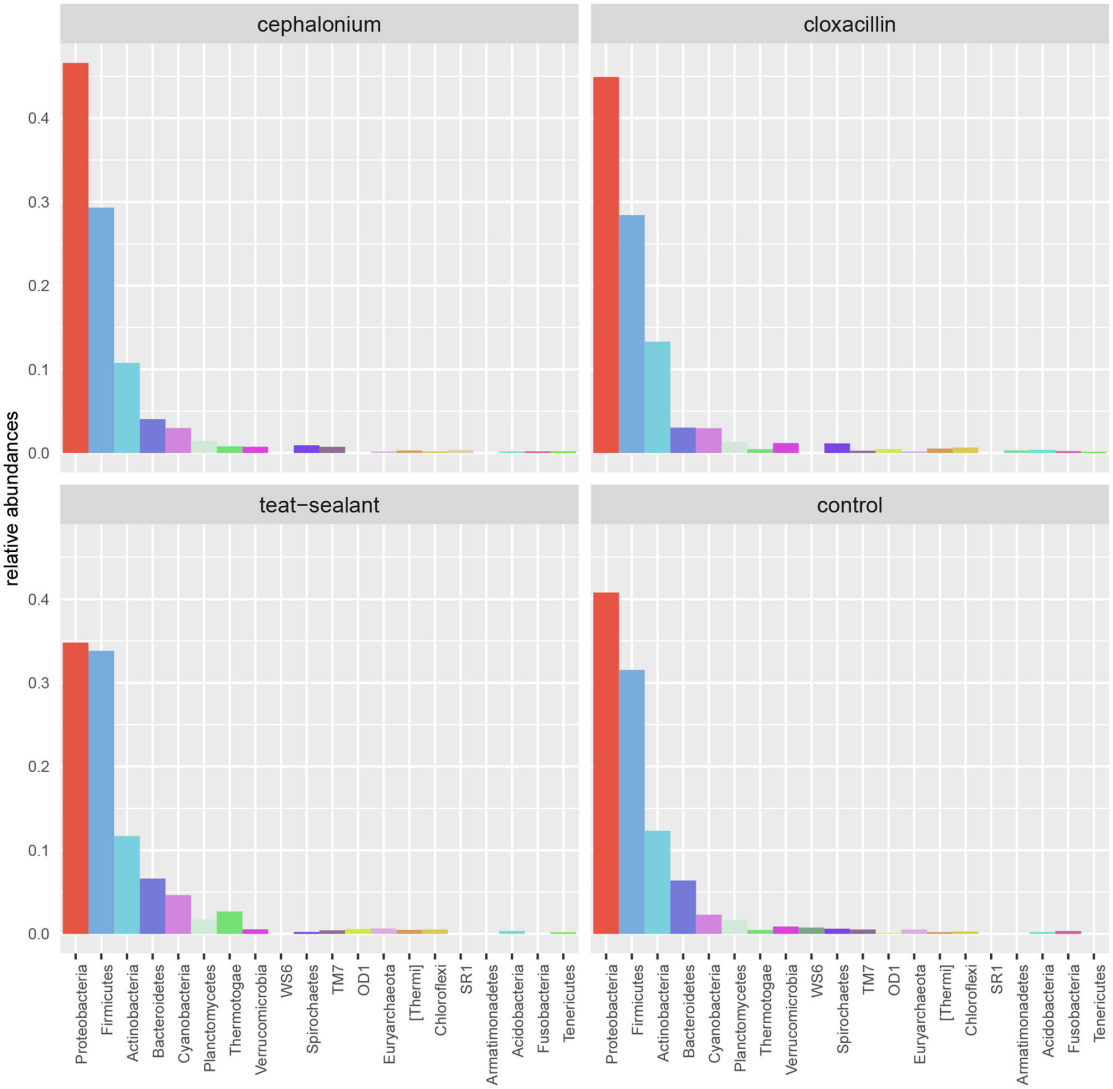
Bar-charts of phylum relative abundances in the dairy cow milk microbiome between mastitis treatments: cephalonium, cloxacillin, teat sealant, controls.

Figure 5 reports the barchart of the relative abundances of phyla in the milk microbiome by dry-off treatment (cephalonium, cloxacillin, teat sealant, and control). The top three most represented phyla were the same in all treatments: *Proteobacteria* (43.0, 38.4, 46.4, 49.4%), *Firmicutes* (31.5, 33.7, 29.7, 31.1%), *Actinobacteria* (12.8, 11.9, 14.5, 10.8%). The fourth most common phylum was *Bacteroidetes* in milk samples from cephalonium (7.44%), cloxacillin (7.49%), and control (3.84%) quarters, *Cyanobacteria* in teat-sealant quarters (3.58%). The average F:B ratio was highest with teat sealant (31.6), followed by cloxacillin (16.4), controls (12.4), and cephalonium (7.8). Only five OTUs were significantly differentially abundant between mastitis treatments (Table 5): the phylum *Tenericutes* (p-value = 0.031), the class *Mollicutes* (*p*-value = 0.017), the order *Acholeplasmatales* (p-value = 0.040), the family *Yaniellaceae* (p-value = 0.036) with its genus *Yaniella* (p-value = 0.036). In all cases, the highest average number of counts was observed in quarters treated with cloxacillin. Overall comparisons of alpha diversity indices between treatments were not significant (Table 4). However, teat-sealant treated quarters showed a decrease in all diversity indices over time, when adjusting for variability at baseline (Figure 4, right pane). The first two dimensions from the (non-metric) multi-dimensional scaling of the Bray-Curtis dissimilarity matrix show extensive overlap between treatments, with no significant clustering (*p*-value from Permanova is 0.157).

**Table 5:** OTUs with significant differential abundance between treatments (alpha ≤ 0.05).

**Taxon**	**OTU**	**Cephalonium**	**Cloxacillin**	**Teat-sealant**	**Controls**	***p*-value**
Phylum	Tenericutes	11.93	59.87	12.33	17.73	3.06e-02
Class	Mollicutes	10.87	54.00	10.07	15.33	1.70e-02
Order	Acholeplasmatales	6.13	31.27	2.20	3.20	4.04e-02
Family	Yaniellaceae	129.40	192.87	24.93	21.93	3.64e-02
Genus	Yaniella	129.40	192.87	24.93	21.93	3.64e-02

*Taxonomic level, specific OTU, counts in quarters treated with cepravin, cloxalene, teat-sealant or controls, *p*-value*.

## 4. Discussion

In this paper, the effect on the milk microbiome of different treatments for mastitis prevention applied during the dry-off period has been investigated. Specifically, the antibiotics cephalonium and cloxacillin have been tested against a non-antibiotic treatment based on the application of an internal teat sealant, on a quarter by quarter basis. Untreated quarters were included in the experimental design as controls. Exploring non-antibiotic alternative options for the prevention of IMI at dry-off in dairy cows is a current research topic of paramount importance in the reduction of widespread antibiotic use in livestock, thereby contributing to alleviate issues related to antibiotic-resistant bacterial strains in veterinary and human medicine (11, 51).

The most interesting results on how the milk microbiome is altered in response to mastitis-prevention treatments are hereby discussed, together with insights into the general composition of the milk microbiome in dairy cows, and its development over the physiological status of lactating animals.

### 4.1. The Milk Microbiome in Response to Treatments

Two antibiotic (cephalonium and cloxacillin) and one non-antibiotic (teat sealant) treatments were compared in this study for their effect on the milk microbiome in Holstein-Friesian dairy cows. The most abundant phyla were consistently *Proteobacteria, Firmicutes*, and *Actinobacteria*, in this order, across treatments and controls. No significant differences were found between treatments and controls in terms of the major alpha and beta diversity indexes and OTU abundances (only 5 OTUs significantly different between treatments, Table 5). This is in line with similar findings from studies on DCT treatments and the milk microbiome: in clinically healthy Holstein-Friesian cows, Derakhshani et al. (24) found no differences in alpha diversity indices before and after BDCT treatment (combination of penicillin G and novobiocin, plus teat sealant), except for Chao1 (higher richness before BDCT than after), although in their study the effect of treatment was confounded with time (dry-off, calving). Bonsaglia et al. (25) also found no significant effect on the milk microbiome of DCT with either antibiotic (ceftiofur) plus teat sealant or teat sealant alone. The use of ITS does not lead to higher infection rates compared to antibiotics in DCT and at the same time appears to be neutral with respect to the milk microbiome (no differences between antibiotics, ITS, and controls): this justifies the replacement of antibiotics with ITS for DCT, which helps reduce the use of antimicrobials in dairy farms.

To reduce confounding from individual variability at the first sampling time (dry-off), alpha diversity indices were adjusted for baseline effect by removing the average values at dry-off (Figure 3, right pane): teat-sealant quarters appear to have lower adjusted diversity (except for Simpson's indices and equitability) compared to the two antimicrobial treatments and controls at calving and 5 DIM. Bonsaglia et al. (25) also found lower Chao1 and Shannon indices at DIM 7 with teat sealant compared to the combination of antibiotic plus teat sealant, though not significant. Bismuth subnitrate products not only act as a physical barrier, but also show inhibitory effect on bacterial growth (16): this can partially explain the efficacy of bismuth-based formulations in the prevention of intramammary infections over the dry period. Other products have been tested as teat sealants for their physical-barrier action, like wax plugs or intramammary polystyrene devices, but were unsuccessful in the long-term protection of cows against IMI and mastitis (52–54).

Contrary to expectations, antibiotic treatments did not cause a marked reduction of the milk microbiome diversity and bacterial counts, as reported also by previous publications (23, 25). This may be related to the specificity of the chosen antimicrobials, which targeted pathogens while leaving the rest of the microbiome practically unaltered [e.g., reduction of *Enterobacteriaceae* upon treatment with ceftiofur in the study of (23)]. Additional factors that can explain any differences between the results reported here and those found in literature include the study design, the time of sampling, the status of cows enrolled in the experiment, the libraries used for 16S rRNA-gene sequencing.

### 4.2. Core Milk Microbiome and Lactation Cycle

Table 2 and Figures 1, 2 offer a description of the milk microbiome in Holstein Friesian cows and of how it evolves over the lactation cycle. The core milk microbiome was defined as OTU shared by 99% of the samples (all): among genera usually associated with the milk milieu (*Lactobacillus, Lactococcus, Propionibacterium*), this includes also bacterial taxa commonly regarded as mastitis pathogens (*Staphylococcus, Pseudomonas, Streptococcus*). Similar findings have been reported in previous studies on the bovine milk microbiome (55). The most abundant taxa detected in the milk microbiota are the phyla *Proteobacteria, Firmicutes, Actinobacteria, Bacteroidetes, Cyanobacteria, Planctomycetes*, the families *Pseudomonadaceae, Streptococcaceae, Propionibacteriaceae, Lactobacillaceae, Moraxellaceae, Alcaligenaceae, Ruminococcaceae*, and the genera *Pseudomonas, Streptococcus, Propionibacterium, Lactobacillus, Corynebacterium, Acinetobacter*. *Proteobacteria, Firmicutes*, and *Actinobacteria* accounted for over 80% of the entire milk microbiota. These results are in agreement with the composition of the healthy milk microbiome previously reported in literature [see (55) for a review]. The milk microbiome from cows with clinical or subclinical mastitis is known to have lower alpha diversity and a different composition (22, 23). In Gram-negative mastitis, for instance, there is a higher relative abundance of *Proteobacteria* in the milk microbiome, specifically, of *Enterobacteriaceae* [over 60%, (23)]: in the present study, the relative abundance of *Enterobacteriaceae* was < 1% at all timepoints (except for control quarters at calving, where it went up to ~ 20%).

**Figure 6:**
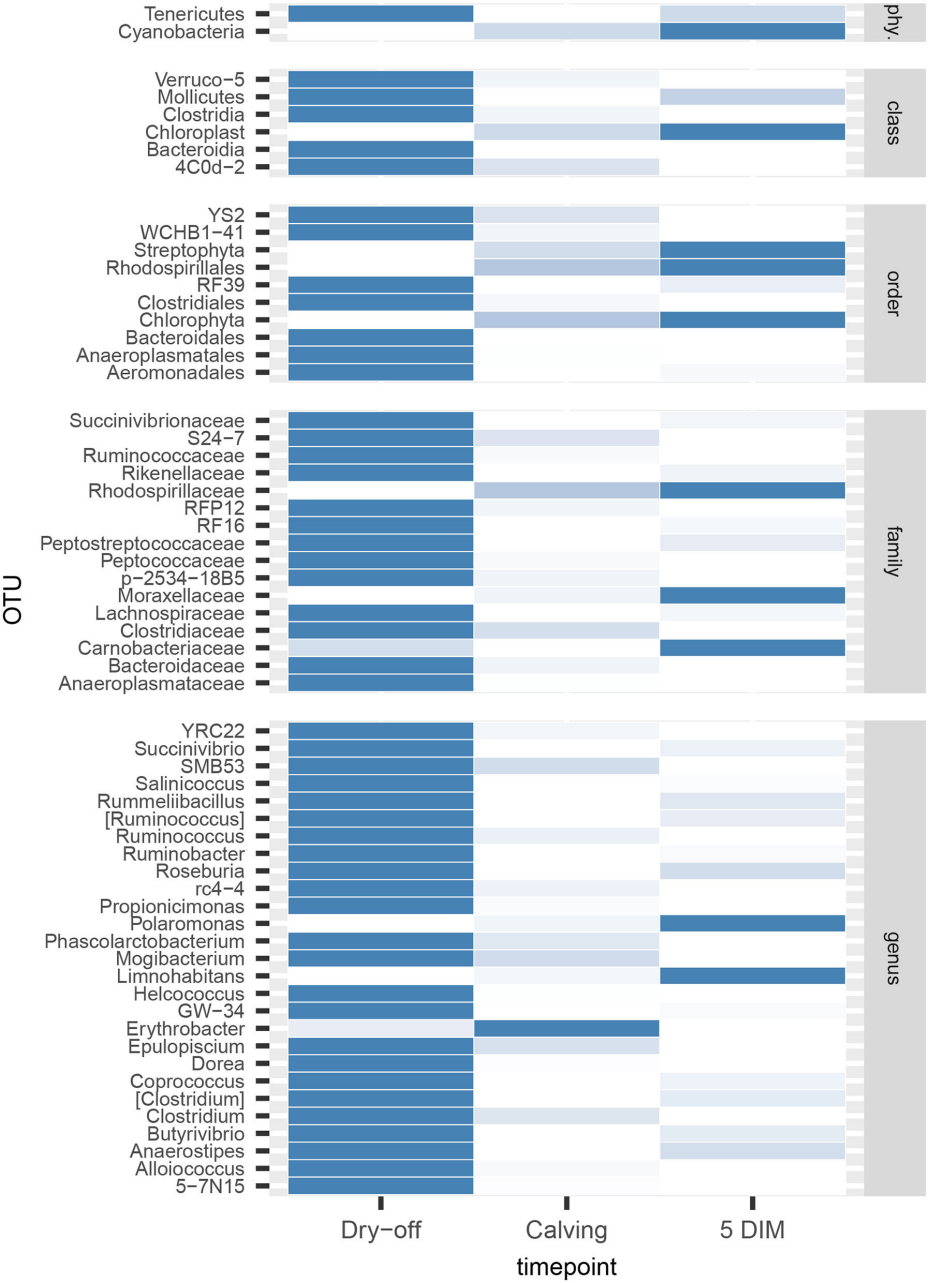
Heatmap of bacterial counts at different timepoints for OTUs found to be significantly different over the lactation cycle. phy.: phylum.

The milk microbiome showed a clear evolution over the lactation cycle -dry-off, calving (colostrum) and 5 DIM- as indicated by the distinct clustering of Bray-Curtis distances, which showed progressive separation from dry-off to calving and then to 5 DIM, and by the significantly different diversity indices between timepoints. In total, 61 OTU showed significant differences in abundance over time. As already reported by Derakhshani et al. (24), the family *Clostridiaceae* and the genus *Butyrivibrio* were significantly overrepresented in pre-DCT milk (dry-off, Table 3). In most cases (50 out of 61), these OTU were more abundant at dry-off (beginning of the experiment) than at subsequent timepoints (Figure 6). The transition from colostrum to mature milk comes along with shifts in the composition of mammary secretions, and some milk components, like milk oligosaccharides, can affect the composition of the milk microbiome (55). In humans, the milk microbiota composition has been reported to be related to host factors like BMI (body mass index) (56): in cattle, body condition (e.g., as measured by BCS: body condition score) is known to change profoundly from dry-off to early lactation, as a consequence of the major physiological changes associated with parturition and the onset of milk production, and it is therefore plausible that it can likewise influence the milk microbiome.

All alpha diversity indices differ significantly between timepoints (except Simpson's E), while the interaction term (timepoint × treatment) was never significant (Table 4, alpha diversity) indicating that overall changes in the milk microbiota diversity over the lactation cycle were independent of treatments. However, the fact that significantly different OTUs were more abundant at the beginning of the experiment (dry-off) may hint at a possible effect of treatments on the depletion of specific microbial taxa, in addition (or in combination) to the physiological influence of the lactation cycle.

When looking at phyla, a shift from *Firmicutes* to *Proteobacteria* as the most abundant phylum was observed between dry-off vs calving and 5 DIM. This is reflected in the evolution of the *Firmicutes*:*Bacteroidetes* (F:B) ratio, which increased with time. The F:B ratio has been used to describe the shift in the gut microbiota associated with aging in humans (57), where it has been reported to increase with time, as found in the present study but on a different timespan. More importantly, the F:B ratio in the gut microbiota is known to play a role in adipogenesis: in studies on obesity in mice and humans, it has been related to higher blood and tissue fat (58, 59), although cause/effect remains unresolved. In Holstein-Friesian cows, Jami et al. (60) observed a strong positive correlation between the F:B ratio in the rumen microbiota and milk-fat yield: this latter finding is mirrored in this study, where a higher F:B ratio has been found in the milk microbiome at the onset of milk production (calving and 5 DIM), when a sharp increase in fat anabolism in the mammary gland takes place. A yet unresolved but interesting question is whether the parallel association between increased F:B ratio and milk yield in both the rumen and milk microbiota is linked to the role of common metabolic pathways in the biosynthesis of fatty acids or, on the other hand, points to interconnections between the two microbial communities.

### 4.3. Implications for the Dry-Cow Therapy

Selective dry-cow therapy (SDCT) consists of treating with antimicrobials only cows with IMI, while non-antibiotic treatments are used on healthy cows. Since 80% of the antibiotics used in dairy farming are used to treat mastitis (23, 61), the adoption of SDCT over BCDT is bound to have a large global effect and can help reduce the spread of antimicrobials resistance (62). Teat sealants are among the non-antibiotic treatments commonly used for SDCT, and it is relevant to assess their impact on the milk microbiome relative to BDCT. Bonsaglia et al. (25) already suggested that cows screened as negative for mastitis during lactation can be managed at dry-off with teat sealant alone without detrimental effects on milk microbiome and bacterial load at first week postpartum. Similar results have been found in the present study, where antimicrobials were directly compared to teat sealant alone rather than in combination, with the added value of testing a first-generation cephalosporin rather than, as did previous works (23, 25), third-generation cephalosporins which are currently not recommended for veterinary use in EU. Our study included cows with low SCC (<200,000 cells mL^−1^) along the whole lactation and without IMI before dry-off, and we found no differences in the prevalence of IMI after calving between quarters treated with different DCT antibiotics and quarters treated only with ITS. ITS play a key role in the success of SDCT programs and their use is highly recommended to achieve good results (7, 63). Importantly, we found no significant differences in the milk microbiome between DCT treatments with antibiotics or ITS. It is however important to be aware of the potential limitations of the present study, which include: (i) the sample size (5 cows, although the statistical power has been increased by adopting a nested quarter-based design); (ii) results are directly applicable only to Holstein-Frisian second-parity cows; (iii) cows were sampled from a single intensive-farming herd in Northern Italy.

Summarizing, the milk microbiomes of healthy dairy cows prophylactically treated with either antibiotics or teat sealants did not show significant differences within 5 DIM from calving. Combined with the analogous efficacy for mastitis prevention and the reduction in the use of antimicrobials, this further supports the adoption of teat sealants as replacement of antibiotic prophylaxis (BDCT) in healthy cows.

## Data Availability Statement

The datasets presented in this study can be found in online repositories. The names of the repository/repositories and accession number(s) can be found below: https://www.ebi.ac.uk/ena/browser/view/PRJEB38332.

## Author Contributions

PM and VB: experimental design and supervision. VB: sampling of biological material. CL and VB: bacteriology. PC and BC: sequencing. FB and AS: bioinformatics. FB: statistical analysis. PC, BC, FB, and PM: writing of the paper. All authors contributed to the article and approved the submitted version.

## Conflict of Interest

The authors declare that the research was conducted in the absence of any commercial or financial relationships that could be construed as a potential conflict of interest.
