# Supplementary material for: A Randomized Controlled Trial of Teat-Sealant and Antibiotic Dry-Cow Treatments for Mastitis Prevention Shows Similar Effect on the Healthy Milk Microbiome
Source: Front Vet Sci. 2020 Sep 2;7:581. doi: 10.3389/fvets.2020.00581 (PMC7492605; doi:10.3389/fvets.2020.00581)
Supplement: Supplementary file 1 [file Data_Sheet_1.PDF]

Supplementary material

FIGURES

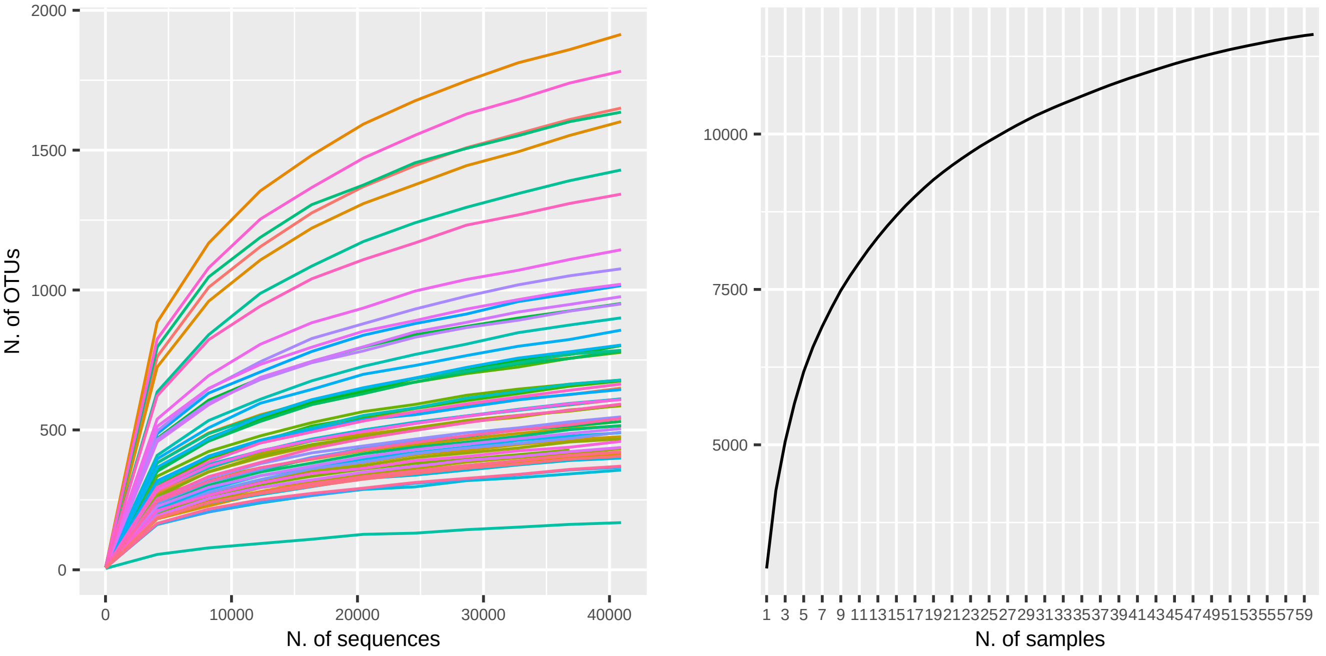

Figure S1. Rarefaction curves. Sequence-based (left) and sample-based (right) rarefaction curves for the sampled rumen microbiotas. Number of detected OTUs on the y-axis; number of sequences (left) and of samples (right) on the x-axis.

TABLES

Table S1. number of available sequences (after quality filtering) and percentage filtering-loss per treatment (quarter) and time-point.

timepoint	N	cephalonium	cloxacillin	teat sealant	control
calving	5.00	174672 +/-25561	152975 +/-31821	116206 +/-34902	176831 +/-122987
dry-off	5.00	112144 +/-40674	105861 +/-43904	124680 +/-60568	93474 +/-23020
5 DIM	5.00	122388 +/-33765	158936 +/-121171	159724 +/-57298	134863 +/-66853
calving	5.00	0.2217	0.2455	0.2806	0.2916
dry-off	5.00	0.2448	0.2562	0.2305	0.2627
5 DIM	5.00	0.2098	0.2428	0.2068	0.2095

Table S2: Average OTU counts per treatment and timepoints

taxa	OTU	treatment	Dry-off	Calving	5 DIM
phylum	Acidobacteria	cephalonium	39.20	14.20	63.20
phylum	Acidobacteria	cloxacillin	18.80	68.00	6.40
phylum	Acidobacteria	teat-sealant	29.20	53.40	399.60
phylum	Acidobacteria	control	35.60	93.00	77.00
phylum	Actinobacteria	cephalonium	9170.20	19086.80	9610.60
phylum	Actinobacteria	cloxacillin	12564.60	9409.40	12896.40
phylum	Actinobacteria	teat-sealant	10280.40	12585.00	19085.40
phylum	Actinobacteria	control	10933.20	10507.20	9159.60
phylum	Armatimonadetes	cephalonium	0.00	0.00	0.00
phylum	Armatimonadetes	cloxacillin	0.00	0.00	0.00
phylum	Armatimonadetes	teat-sealant	0.00	0.40	76.80
phylum	Armatimonadetes	control	0.00	0.00	0.00
phylum	Bacteroidetes	cephalonium	5226.60	12060.60	4774.80
phylum	Bacteroidetes	cloxacillin	10457.00	4066.00	7516.80
phylum	Bacteroidetes	teat-sealant	3880.20	1299.20	4953.20
phylum	Bacteroidetes	control	4644.20	3681.80	2511.20
phylum	Chloroflexi	cephalonium	122.00	237.80	7.60
phylum	Chloroflexi	cloxacillin	92.00	0.20	261.80
phylum	Chloroflexi	teat-sealant	0.20	0.20	200.60
phylum	Chloroflexi	control	69.40	16.40	0.20
phylum	Cyanobacteria	cephalonium	155.00	2149.40	5604.40
phylum	Cyanobacteria	cloxacillin	433.00	2545.20	15221.20
phylum	Cyanobacteria	teat-sealant	305.60	2629.60	7381.60
phylum	Cyanobacteria	control	188.40	3020.40	5623.20
phylum	Elusimicrobia	cephalonium	0.00	32.60	0.00
phylum	Elusimicrobia	cloxacillin	1.00	0.00	0.00
phylum	Elusimicrobia	teat-sealant	0.00	0.00	0.00
phylum	Elusimicrobia	control	0.00	0.00	0.00
phylum	Euryarchaeota	cephalonium	252.80	43.20	20.40
phylum	Euryarchaeota	cloxacillin	443.00	337.80	45.00
phylum	Euryarchaeota	teat-sealant	103.00	35.40	44.60
phylum	Euryarchaeota	control	132.20	43.80	23.40
phylum	Firmicutes	cephalonium	25328.00	31529.20	36367.00
phylum	Firmicutes	cloxacillin	40144.80	25442.00	33506.00
phylum	Firmicutes	teat-sealant	25948.80	21648.40	38121.40
phylum	Firmicutes	control	29523.40	28289.80	29925.00
phylum	Fusobacteria	cephalonium	24.20	126.40	86.00
phylum	Fusobacteria	cloxacillin	6.60	0.00	7.20
phylum	Fusobacteria	teat-sealant	52.20	2.00	104.40
phylum	Fusobacteria	control	6.60	50.40	7.60
phylum	Lentisphaerae	cephalonium	1.20	0.00	0.00
phylum	Lentisphaerae	cloxacillin	10.00	0.00	0.00
phylum	Lentisphaerae	teat-sealant	8.40	0.00	0.00
phylum	Lentisphaerae	control	0.00	0.00	0.00

phylum	OD1	cephalonium	0.00	0.00	24.60
phylum	OD1	cloxacillin	0.20	110.80	0.40
phylum	OD1	teat-sealant	0.00	0.00	110.80
phylum	OD1	control	0.00	0.00	16.40
phylum	Planctomycetes	cephalonium	163.60	1326.60	1928.20
phylum	Planctomycetes	cloxacillin	29.80	719.40	1894.20
phylum	Planctomycetes	teat-sealant	114.00	1426.00	1267.80
phylum	Planctomycetes	control	472.20	919.20	1207.40
phylum	Proteobacteria	cephalonium	27427.00	47459.00	52586.80
phylum	Proteobacteria	cloxacillin	23135.40	30803.20	59003.00
phylum	Proteobacteria	teat-sealant	30276.60	46680.40	56864.80
phylum	Proteobacteria	control	32312.20	57803.40	49558.60
phylum	Spirochaetes	cephalonium	185.60	1.20	543.80
phylum	Spirochaetes	cloxacillin	81.20	0.00	74.40
phylum	Spirochaetes	teat-sealant	166.20	0.20	0.20
phylum	Spirochaetes	control	133.80	0.40	0.00
phylum	SR1	cephalonium	0.00	0.00	0.00
phylum	SR1	cloxacillin	6.80	0.00	0.00
phylum	SR1	teat-sealant	0.00	0.00	0.20
phylum	SR1	control	0.00	0.20	46.40
phylum	Tenericutes	cephalonium	27.40	1.20	7.20
phylum	Tenericutes	cloxacillin	124.80	1.80	53.00
phylum	Tenericutes	teat-sealant	36.80	0.00	0.20
phylum	Tenericutes	control	42.20	2.20	8.80
phylum	[Thermi]	cephalonium	71.60	129.80	0.00
phylum	[Thermi]	cloxacillin	286.80	3.40	285.20
phylum	[Thermi]	teat-sealant	204.00	6.20	0.20
phylum	[Thermi]	control	64.00	22.80	53.20
phylum	Thermotogae	cephalonium	25.60	131.40	0.00
phylum	Thermotogae	cloxacillin	0.20	464.80	0.60
phylum	Thermotogae	teat-sealant	0.20	0.20	108.80
phylum	Thermotogae	control	0.40	202.80	0.40
phylum	TM7	cephalonium	116.00	400.60	205.40
phylum	TM7	cloxacillin	515.60	0.60	97.80
phylum	TM7	teat-sealant	148.40	1.20	42.00
phylum	TM7	control	17.40	7.60	118.60
phylum	Verrucomicrobia	cephalonium	114.20	335.00	866.40
phylum	Verrucomicrobia	cloxacillin	501.60	193.40	357.60
phylum	Verrucomicrobia	teat-sealant	261.40	1234.80	152.60
phylum	Verrucomicrobia	control	256.40	256.60	384.40
phylum	WPS-2	cephalonium	0.00	45.60	3.00
phylum	WPS-2	cloxacillin	0.00	0.00	0.00
phylum	WPS-2	teat-sealant	0.00	0.00	3.80
phylum	WPS-2	control	2.40	0.00	0.00
phylum	WS6	cephalonium	0.00	0.40	140.20

phylum	WS6	cloxacillin	0.00	0.00	0.00
phylum	WS6	teat-sealant	0.00	0.00	0.00
phylum	WS6	control	0.00	0.00	0.00
class	4C0d-2	cephalonium	40.20	70.80	0.00
class	4C0d-2	cloxacillin	252.20	0.40	0.00
class	4C0d-2	teat-sealant	148.80	0.60	0.20
class	4C0d-2	control	34.80	29.80	0.00
class	Acidimicrobiia	cephalonium	5.60	0.00	0.00
class	Acidimicrobiia	cloxacillin	3.80	0.00	0.00
class	Acidimicrobiia	teat-sealant	0.00	61.40	40.40
class	Acidimicrobiia	control	0.00	0.00	0.00
class	Acidobacteria-6	cephalonium	0.00	0.00	0.00
class	Acidobacteria-6	cloxacillin	0.00	4.00	0.20
class	Acidobacteria-6	teat-sealant	0.00	0.00	0.00
class	Acidobacteria-6	control	0.00	0.00	0.00
class	Actinobacteria	cephalonium	9116.80	18678.40	9516.00
class	Actinobacteria	cloxacillin	12393.40	9363.60	12476.40
class	Actinobacteria	teat-sealant	10210.00	12452.20	18950.40
class	Actinobacteria	control	10872.80	10456.60	8957.80
class	Alphaproteobacteria	cephalonium	1646.60	5616.40	3566.40
class	Alphaproteobacteria	cloxacillin	2760.00	2402.00	5705.00
class	Alphaproteobacteria	teat-sealant	1327.00	4291.80	4554.60
class	Alphaproteobacteria	control	1949.20	2325.20	4035.60
class	Anaerolineae	cephalonium	0.20	189.60	0.00
class	Anaerolineae	cloxacillin	0.00	0.00	258.20
class	Anaerolineae	teat-sealant	0.20	0.20	156.80
class	Anaerolineae	control	0.00	0.20	0.00
class	Bacilli	cephalonium	15367.00	26123.20	32000.80
class	Bacilli	cloxacillin	17367.60	23699.20	31370.80
class	Bacilli	teat-sealant	18204.40	19891.00	35471.60
class	Bacilli	control	19759.00	22739.60	27057.60
class	Bacteroidia	cephalonium	3012.20	1392.00	1392.80
class	Bacteroidia	cloxacillin	5820.60	577.00	804.40
class	Bacteroidia	teat-sealant	2650.20	12.20	473.60
class	Bacteroidia	control	3101.20	1041.20	354.20
class	Betaproteobacteria	cephalonium	10201.00	14715.20	20080.00
class	Betaproteobacteria	cloxacillin	7252.40	11218.00	18506.80
class	Betaproteobacteria	teat-sealant	10898.80	17815.40	21642.40
class	Betaproteobacteria	control	11787.40	12026.20	18776.20
class	[Chloracidobacteria]	cephalonium	0.00	1.40	0.00
class	[Chloracidobacteria]	cloxacillin	9.80	0.00	0.00
class	[Chloracidobacteria]	teat-sealant	0.00	0.00	0.00
class	[Chloracidobacteria]	control	0.00	24.20	26.40
class	Chloroplast	cephalonium	108.80	1951.60	5574.40
class	Chloroplast	cloxacillin	174.40	2514.00	15221.20

class	Chloroplast	teat-sealant	156.80	2593.60	7381.40
class	Chloroplast	control	153.60	2967.40	5623.20
class	Clostridia	cephalonium	9911.80	5340.00	4208.40
class	Clostridia	cloxacillin	22553.80	1739.40	2081.40
class	Clostridia	teat-sealant	7722.80	1757.40	2649.80
class	Clostridia	control	9732.80	5535.80	2604.20
class	Coriobacteriia	cephalonium	46.40	367.60	0.20
class	Coriobacteriia	cloxacillin	146.40	26.40	417.20
class	Coriobacteriia	teat-sealant	59.20	0.20	60.80
class	Coriobacteriia	control	55.40	36.80	157.80
class	Cytophagia	cephalonium	227.40	668.20	182.60
class	Cytophagia	cloxacillin	1889.40	231.60	605.20
class	Cytophagia	teat-sealant	43.20	162.20	157.20
class	Cytophagia	control	111.40	174.20	184.20
class	DA052	cephalonium	21.80	12.60	0.00
class	DA052	cloxacillin	5.80	63.60	0.00
class	DA052	teat-sealant	27.00	45.60	202.80
class	DA052	control	25.60	4.20	0.80
class	Deinococci	cephalonium	71.60	129.80	0.00
class	Deinococci	cloxacillin	286.80	3.40	285.20
class	Deinococci	teat-sealant	204.00	6.20	0.20
class	Deinococci	control	64.00	22.80	53.20
class	Deltaproteobacteria	cephalonium	7.60	105.40	13.20
class	Deltaproteobacteria	cloxacillin	35.40	9.80	32.00
class	Deltaproteobacteria	teat-sealant	25.80	0.00	1.20
class	Deltaproteobacteria	control	46.00	0.00	5.00
class	Elusimicrobia	cephalonium	0.00	32.60	0.00
class	Elusimicrobia	cloxacillin	1.00	0.00	0.00
class	Elusimicrobia	teat-sealant	0.00	0.00	0.00
class	Elusimicrobia	control	0.00	0.00	0.00
class	Epsilonproteobacteria	cephalonium	64.20	0.00	0.20
class	Epsilonproteobacteria	cloxacillin	107.00	25.80	169.40
class	Epsilonproteobacteria	teat-sealant	80.40	3.80	0.00
class	Epsilonproteobacteria	control	57.60	50.00	0.20
class	Erysipelotrichi	cephalonium	49.20	66.00	157.80
class	Erysipelotrichi	cloxacillin	223.40	3.40	53.80
class	Erysipelotrichi	teat-sealant	21.60	0.00	0.00
class	Erysipelotrichi	control	31.60	14.40	263.20
class	[Fimbriimonadia]	cephalonium	0.00	0.00	0.00
class	[Fimbriimonadia]	cloxacillin	0.00	0.00	0.00
class	[Fimbriimonadia]	teat-sealant	0.00	0.40	76.80
class	[Fimbriimonadia]	control	0.00	0.00	0.00
class	Flavobacteriia	cephalonium	1502.20	7259.60	2087.80
class	Flavobacteriia	cloxacillin	2025.80	2110.40	4736.20
class	Flavobacteriia	teat-sealant	776.00	284.20	3029.20

class	Flavobacteriia	control	906.40	1353.40	1316.80
class	Fusobacteriia	cephalonium	24.20	126.40	86.00
class	Fusobacteriia	cloxacillin	6.60	0.00	7.20
class	Fusobacteriia	teat-sealant	52.20	2.00	104.40
class	Fusobacteriia	control	6.60	50.40	7.60
class	Gammaproteobacteria	cephalonium	15507.60	27022.00	28927.00
class	Gammaproteobacteria	cloxacillin	12980.60	17147.60	34589.60
class	Gammaproteobacteria	teat-sealant	17944.60	24569.40	30666.60
class	Gammaproteobacteria	control	18470.60	43402.00	26741.20
class	[Lentisphaeria]	cephalonium	1.20	0.00	0.00
class	[Lentisphaeria]	cloxacillin	10.00	0.00	0.00
class	[Lentisphaeria]	teat-sealant	8.40	0.00	0.00
class	[Lentisphaeria]	control	0.00	0.00	0.00
class	MB-A2-108	cephalonium	0.00	0.00	2.60
class	MB-A2-108	cloxacillin	2.00	0.00	0.00
class	MB-A2-108	teat-sealant	0.00	0.00	0.00
class	MB-A2-108	control	0.00	0.00	0.00
class	Methanobacteria	cephalonium	252.80	43.20	20.40
class	Methanobacteria	cloxacillin	441.60	337.80	45.00
class	Methanobacteria	teat-sealant	103.00	35.40	44.60
class	Methanobacteria	control	130.20	43.80	23.40
class	Methanomicrobia	cephalonium	0.00	0.00	0.00
class	Methanomicrobia	cloxacillin	1.40	0.00	0.00
class	Methanomicrobia	teat-sealant	0.00	0.00	0.00
class	Methanomicrobia	control	2.00	0.00	0.00
class	Mollicutes	cephalonium	24.20	1.20	7.20
class	Mollicutes	cloxacillin	108.60	0.40	53.00
class	Mollicutes	teat-sealant	30.00	0.00	0.20
class	Mollicutes	control	35.00	2.20	8.80
class	Opitutae	cephalonium	0.00	0.00	0.00
class	Opitutae	cloxacillin	0.00	0.00	0.00
class	Opitutae	teat-sealant	0.00	456.80	0.20
class	Opitutae	control	0.00	0.00	0.00
class	Oscillatoriophycideae	cephalonium	6.00	127.00	30.00
class	Oscillatoriophycideae	cloxacillin	6.40	30.80	0.00
class	Oscillatoriophycideae	teat-sealant	0.00	35.40	0.00
class	Oscillatoriophycideae	control	0.00	23.20	0.00
class	Phycisphaerae	cephalonium	0.00	0.00	33.40
class	Phycisphaerae	cloxacillin	0.00	0.00	0.00
class	Phycisphaerae	teat-sealant	0.00	0.00	0.00
class	Phycisphaerae	control	0.00	79.40	0.00
class	Planctomycetia	cephalonium	163.60	1326.60	1894.80
class	Planctomycetia	cloxacillin	29.80	719.40	1894.20
class	Planctomycetia	teat-sealant	114.00	1426.00	1267.80
class	Planctomycetia	control	472.20	839.80	1207.40

class	RF3	cephalonium	3.20	0.00	0.00
class	RF3	cloxacillin	16.20	1.40	0.00
class	RF3	teat-sealant	6.80	0.00	0.00
class	RF3	control	7.20	0.00	0.00
class	[Saprospirae]	cephalonium	63.20	150.20	548.60
class	[Saprospirae]	cloxacillin	32.00	85.80	224.60
class	[Saprospirae]	teat-sealant	92.80	811.80	559.20
class	[Saprospirae]	control	360.60	548.60	552.40
class	SC72	cephalonium	0.00	0.40	140.20
class	SC72	cloxacillin	0.00	0.00	0.00
class	SC72	teat-sealant	0.00	0.00	0.00
class	SC72	control	0.00	0.00	0.00
class	Solibacteres	cephalonium	17.40	0.20	63.20
class	Solibacteres	cloxacillin	3.20	0.40	6.20
class	Solibacteres	teat-sealant	2.20	7.80	196.80
class	Solibacteres	control	10.00	64.60	49.80
class	[Spartobacteria]	cephalonium	0.00	0.00	0.00
class	[Spartobacteria]	cloxacillin	21.00	0.00	88.80
class	[Spartobacteria]	teat-sealant	0.00	0.00	0.00
class	[Spartobacteria]	control	0.00	107.20	0.00
class	Sphingobacteriia	cephalonium	421.60	2590.60	563.00
class	Sphingobacteriia	cloxacillin	689.20	1061.20	1146.40
class	Sphingobacteriia	teat-sealant	318.00	28.80	734.00
class	Sphingobacteriia	control	164.60	564.40	103.60
class	Spirochaetes	cephalonium	185.60	1.20	543.80
class	Spirochaetes	cloxacillin	81.20	0.00	74.40
class	Spirochaetes	teat-sealant	166.20	0.20	0.20
class	Spirochaetes	control	133.80	0.40	0.00
class	Thermoleophilia	cephalonium	1.40	40.80	91.80
class	Thermoleophilia	cloxacillin	19.00	19.40	2.80
class	Thermoleophilia	teat-sealant	11.20	71.20	33.80
class	Thermoleophilia	control	5.00	13.80	44.00
class	Thermomicrobia	cephalonium	121.80	48.20	7.60
class	Thermomicrobia	cloxacillin	92.00	0.20	3.60
class	Thermomicrobia	teat-sealant	0.00	0.00	43.80
class	Thermomicrobia	control	69.40	16.20	0.20
class	Thermotogae	cephalonium	25.60	131.40	0.00
class	Thermotogae	cloxacillin	0.20	464.80	0.60
class	Thermotogae	teat-sealant	0.20	0.20	108.80
class	Thermotogae	control	0.40	202.80	0.40
class	TM7-1	cephalonium	0.00	0.00	0.00
class	TM7-1	cloxacillin	6.80	0.00	0.00
class	TM7-1	teat-sealant	0.00	0.00	0.00
class	TM7-1	control	0.00	0.00	0.00
class	TM7-3	cephalonium	116.00	400.60	205.40

class	TM7-3	cloxacillin	508.80	0.60	97.80
class	TM7-3	teat-sealant	148.40	1.20	42.00
class	TM7-3	control	17.40	7.60	118.60
class	Verruco-5	cephalonium	15.00	0.20	0.00
class	Verruco-5	cloxacillin	46.60	0.00	0.00
class	Verruco-5	teat-sealant	25.60	0.40	0.00
class	Verruco-5	control	11.60	7.20	0.00
class	Verrucomicrobiae	cephalonium	99.20	334.80	866.40
class	Verrucomicrobiae	cloxacillin	434.00	193.40	268.80
class	Verrucomicrobiae	teat-sealant	235.80	777.60	152.40
class	Verrucomicrobiae	control	244.80	142.20	384.40
class	ZB2	cephalonium	0.00	0.00	24.60
class	ZB2	cloxacillin	0.20	110.80	0.40
class	ZB2	teat-sealant	0.00	0.00	110.80
class	ZB2	control	0.00	0.00	16.40
order	0319-7L14	cephalonium	0.00	0.00	2.60
order	0319-7L14	cloxacillin	2.00	0.00	0.00
order	0319-7L14	teat-sealant	0.00	0.00	0.00
order	0319-7L14	control	0.00	0.00	0.00
order	Acholeplasmatales	cephalonium	12.20	0.00	6.20
order	Acholeplasmatales	cloxacillin	40.40	0.40	53.00
order	Acholeplasmatales	teat-sealant	6.60	0.00	0.00
order	Acholeplasmatales	control	9.40	0.20	0.00
order	Acidimicrobiales	cephalonium	5.60	0.00	0.00
order	Acidimicrobiales	cloxacillin	3.80	0.00	0.00
order	Acidimicrobiales	teat-sealant	0.00	61.40	40.40
order	Acidimicrobiales	control	0.00	0.00	0.00
order	Actinomycetales	cephalonium	8945.60	18556.00	9338.00
order	Actinomycetales	cloxacillin	11591.00	9241.20	12407.60
order	Actinomycetales	teat-sealant	10087.20	12299.60	18928.80
order	Actinomycetales	control	10604.60	9320.00	8957.60
order	Aeromonadales	cephalonium	174.00	1.00	72.20
order	Aeromonadales	cloxacillin	493.80	30.00	33.60
order	Aeromonadales	teat-sealant	150.60	0.40	22.60
order	Aeromonadales	control	482.00	37.40	0.60
order	Alteromonadales	cephalonium	91.20	1049.80	89.80
order	Alteromonadales	cloxacillin	169.00	129.60	267.60
order	Alteromonadales	teat-sealant	20.60	15.40	122.00
order	Alteromonadales	control	72.80	204.40	34.80
order	Anaeroplasmatales	cephalonium	0.00	0.00	0.00
order	Anaeroplasmatales	cloxacillin	44.20	0.00	0.00
order	Anaeroplasmatales	teat-sealant	11.40	0.00	0.00
order	Anaeroplasmatales	control	12.00	0.20	0.00
order	Bacillales	cephalonium	3654.00	4109.00	2872.40
order	Bacillales	cloxacillin	5385.20	2158.40	3676.00

order	Bacillales	teat-sealant	3234.60	753.20	4549.40
order	Bacillales	control	2903.60	1501.60	3215.40
order	Bacteroidales	cephalonium	3012.20	1392.00	1392.80
order	Bacteroidales	cloxacillin	5820.60	577.00	804.40
order	Bacteroidales	teat-sealant	2650.20	12.20	473.60
order	Bacteroidales	control	3101.20	1041.20	354.20
order	BD7-3	cephalonium	0.00	0.00	0.00
order	BD7-3	cloxacillin	0.00	0.00	0.00
order	BD7-3	teat-sealant	0.00	0.00	0.00
order	BD7-3	control	0.20	0.40	69.80
order	Bifidobacteriales	cephalonium	171.20	122.40	178.00
order	Bifidobacteriales	cloxacillin	802.40	122.40	68.80
order	Bifidobacteriales	teat-sealant	122.80	152.60	21.60
order	Bifidobacteriales	control	268.20	1136.60	0.20
order	Burkholderiales	cephalonium	10129.20	14303.40	19969.00
order	Burkholderiales	cloxacillin	7163.60	11160.60	17995.20
order	Burkholderiales	teat-sealant	10772.20	17209.60	21499.60
order	Burkholderiales	control	11602.80	11963.60	18158.00
order	Caldilineales	cephalonium	0.20	189.60	0.00
order	Caldilineales	cloxacillin	0.00	0.00	258.20
order	Caldilineales	teat-sealant	0.20	0.20	156.80
order	Caldilineales	control	0.00	0.20	0.00
order	Campylobacterales	cephalonium	64.20	0.00	0.20
order	Campylobacterales	cloxacillin	107.00	25.80	169.40
order	Campylobacterales	teat-sealant	80.40	3.80	0.00
order	Campylobacterales	control	57.60	50.00	0.20
order	Cardiobacteriales	cephalonium	18.40	0.00	0.00
order	Cardiobacteriales	cloxacillin	0.40	0.20	198.00
order	Cardiobacteriales	teat-sealant	0.00	0.00	0.00
order	Cardiobacteriales	control	0.00	0.00	0.00
order	Caulobacterales	cephalonium	216.00	521.00	141.80
order	Caulobacterales	cloxacillin	121.20	198.80	111.20
order	Caulobacterales	teat-sealant	100.20	277.80	257.60
order	Caulobacterales	control	147.00	137.20	233.60
order	Chlorophyta	cephalonium	0.20	71.80	496.20
order	Chlorophyta	cloxacillin	1.40	866.40	2216.00
order	Chlorophyta	teat-sealant	0.60	511.00	1204.00
order	Chlorophyta	control	2.20	734.60	1157.00
order	Chroococcales	cephalonium	0.00	83.60	30.00
order	Chroococcales	cloxacillin	0.00	0.00	0.00
order	Chroococcales	teat-sealant	0.00	0.00	0.00
order	Chroococcales	control	0.00	0.00	0.00
order	[Chthoniobacterales]	cephalonium	0.00	0.00	0.00
order	[Chthoniobacterales]	cloxacillin	21.00	0.00	88.80
order	[Chthoniobacterales]	teat-sealant	0.00	0.00	0.00

order	[Chthoniobacterales]	control	0.00	107.20	0.00
order	Clostridiales	cephalonium	9911.80	5151.40	4134.60
order	Clostridiales	cloxacillin	22553.80	1739.40	2081.40
order	Clostridiales	teat-sealant	7722.80	1532.00	2649.80
order	Clostridiales	control	9630.40	4793.80	2065.40
order	Coriobacteriales	cephalonium	46.40	367.60	0.20
order	Coriobacteriales	cloxacillin	146.40	26.40	417.20
order	Coriobacteriales	teat-sealant	59.20	0.20	60.80
order	Coriobacteriales	control	55.40	36.80	157.80
order	CW040	cephalonium	51.40	390.40	61.40
order	CW040	cloxacillin	401.80	0.00	6.80
order	CW040	teat-sealant	110.20	0.20	2.00
order	CW040	control	15.20	0.60	0.00
order	Cytophagales	cephalonium	227.40	668.20	182.60
order	Cytophagales	cloxacillin	1889.40	231.60	605.20
order	Cytophagales	teat-sealant	43.20	162.20	157.20
order	Cytophagales	control	111.40	174.20	184.20
order	Deinococcales	cephalonium	71.60	129.80	0.00
order	Deinococcales	cloxacillin	277.20	3.20	26.80
order	Deinococcales	teat-sealant	143.00	1.80	0.20
order	Deinococcales	control	64.00	22.80	53.20
order	Desulfovibrionales	cephalonium	4.00	95.20	13.20
order	Desulfovibrionales	cloxacillin	33.40	0.00	3.00
order	Desulfovibrionales	teat-sealant	25.80	0.00	1.20
order	Desulfovibrionales	control	46.00	0.00	0.00
order	Ellin329	cephalonium	0.00	0.00	0.20
order	Ellin329	cloxacillin	0.00	0.20	6.00
order	Ellin329	teat-sealant	1.00	0.60	0.00
order	Ellin329	control	0.00	0.00	0.60
order	Ellin6513	cephalonium	21.80	12.60	0.00
order	Ellin6513	cloxacillin	5.80	63.60	0.00
order	Ellin6513	teat-sealant	27.00	45.60	202.80
order	Ellin6513	control	25.60	4.20	0.80
order	Elusimicrobiales	cephalonium	0.00	32.60	0.00
order	Elusimicrobiales	cloxacillin	1.00	0.00	0.00
order	Elusimicrobiales	teat-sealant	0.00	0.00	0.00
order	Elusimicrobiales	control	0.00	0.00	0.00
order	Enterobacteriales	cephalonium	71.20	217.00	80.80
order	Enterobacteriales	cloxacillin	119.00	111.00	136.00
order	Enterobacteriales	teat-sealant	688.80	86.60	94.80
order	Enterobacteriales	control	45.60	18906.80	377.60
order	Erysipelotrichales	cephalonium	49.20	66.00	157.80
order	Erysipelotrichales	cloxacillin	223.40	3.40	53.80
order	Erysipelotrichales	teat-sealant	21.60	0.00	0.00
order	Erysipelotrichales	control	31.60	14.40	263.20

order	EW055	cephalonium	22.80	7.00	23.00
order	EW055	cloxacillin	27.20	0.40	43.40
order	EW055	teat-sealant	26.20	0.60	0.20
order	EW055	control	0.20	2.20	8.40
order	[Fimbriimonadales]	cephalonium	0.00	0.00	0.00
order	[Fimbriimonadales]	cloxacillin	0.00	0.00	0.00
order	[Fimbriimonadales]	teat-sealant	0.00	0.40	76.80
order	[Fimbriimonadales]	control	0.00	0.00	0.00
order	Flavobacteriales	cephalonium	1502.20	7259.60	2087.80
order	Flavobacteriales	cloxacillin	2025.80	2110.40	4736.20
order	Flavobacteriales	teat-sealant	776.00	284.20	3029.20
order	Flavobacteriales	control	906.40	1353.40	1316.80
order	Fusobacteriales	cephalonium	24.20	126.40	86.00
order	Fusobacteriales	cloxacillin	6.60	0.00	7.20
order	Fusobacteriales	teat-sealant	52.20	2.00	104.40
order	Fusobacteriales	control	6.60	50.40	7.60
order	Gaiellales	cephalonium	1.20	40.60	1.00
order	Gaiellales	cloxacillin	18.80	6.40	2.80
order	Gaiellales	teat-sealant	0.40	70.40	20.40
order	Gaiellales	control	5.00	13.60	18.20
order	Gemellales	cephalonium	4.80	0.00	0.00
order	Gemellales	cloxacillin	0.60	51.00	10.60
order	Gemellales	teat-sealant	0.00	0.00	0.00
order	Gemellales	control	0.00	0.00	0.00
order	Gemmatales	cephalonium	100.20	1298.20	1517.00
order	Gemmatales	cloxacillin	29.40	698.20	1790.20
order	Gemmatales	teat-sealant	114.00	1425.60	935.40
order	Gemmatales	control	372.80	835.80	1207.20
order	GMD14H09	cephalonium	3.60	10.20	0.00
order	GMD14H09	cloxacillin	0.80	9.80	12.40
order	GMD14H09	teat-sealant	0.00	0.00	0.00
order	GMD14H09	control	0.00	0.00	5.00
order	Halanaerobiales	cephalonium	0.00	1.60	0.00
order	Halanaerobiales	cloxacillin	0.00	0.00	0.00
order	Halanaerobiales	teat-sealant	0.00	0.00	0.00
order	Halanaerobiales	control	1.00	348.40	0.40
order	iii1-15	cephalonium	0.00	0.00	0.00
order	iii1-15	cloxacillin	0.00	4.00	0.20
order	iii1-15	teat-sealant	0.00	0.00	0.00
order	iii1-15	control	0.00	0.00	0.00
order	JG30-KF-CM45	cephalonium	121.80	48.20	7.60
order	JG30-KF-CM45	cloxacillin	92.00	0.20	3.60
order	JG30-KF-CM45	teat-sealant	0.00	0.00	43.80
order	JG30-KF-CM45	control	69.40	16.20	0.20
order	Lactobacillales	cephalonium	11379.00	21956.60	29125.60

order	Lactobacillales	cloxacillin	11275.20	21485.40	27668.80
order	Lactobacillales	teat-sealant	14753.80	19107.60	30920.20
order	Lactobacillales	control	14892.00	21090.80	23840.60
order	Legionellales	cephalonium	297.80	2.60	46.20
order	Legionellales	cloxacillin	80.80	245.20	3.20
order	Legionellales	teat-sealant	260.40	553.80	91.40
order	Legionellales	control	3.00	644.80	1.20
order	MBA08	cephalonium	0.00	0.00	73.60
order	MBA08	cloxacillin	0.00	0.00	0.00
order	MBA08	teat-sealant	0.00	225.40	0.00
order	MBA08	control	0.20	391.20	297.20
order	Methanobacteriales	cephalonium	252.80	43.20	20.40
order	Methanobacteriales	cloxacillin	441.60	337.80	45.00
order	Methanobacteriales	teat-sealant	103.00	35.40	44.60
order	Methanobacteriales	control	130.20	43.80	23.40
order	Methanosarcinales	cephalonium	0.00	0.00	0.00
order	Methanosarcinales	cloxacillin	1.40	0.00	0.00
order	Methanosarcinales	teat-sealant	0.00	0.00	0.00
order	Methanosarcinales	control	2.00	0.00	0.00
order	Methylophilales	cephalonium	0.00	0.00	0.00
order	Methylophilales	cloxacillin	0.00	0.00	193.00
order	Methylophilales	teat-sealant	0.00	156.60	0.20
order	Methylophilales	control	0.00	0.60	475.80
order	ML615J-28	cephalonium	3.20	0.00	0.00
order	ML615J-28	cloxacillin	16.20	1.40	0.00
order	ML615J-28	teat-sealant	6.80	0.00	0.00
order	ML615J-28	control	7.20	0.00	0.00
order	Myxococcales	cephalonium	0.00	0.00	0.00
order	Myxococcales	cloxacillin	1.20	0.00	16.60
order	Myxococcales	teat-sealant	0.00	0.00	0.00
order	Myxococcales	control	0.00	0.00	0.00
order	Neisseriales	cephalonium	31.80	234.80	31.00
order	Neisseriales	cloxacillin	72.60	51.60	297.80
order	Neisseriales	teat-sealant	126.20	424.00	127.20
order	Neisseriales	control	176.00	58.60	68.20
order	Oceanospirillales	cephalonium	63.40	562.40	117.40
order	Oceanospirillales	cloxacillin	68.00	260.40	148.60
order	Oceanospirillales	teat-sealant	50.60	189.60	65.00
order	Oceanospirillales	control	115.80	80.20	104.20
order	OPB54	cephalonium	0.00	7.00	0.00
order	OPB54	cloxacillin	0.00	0.00	0.00
order	OPB54	teat-sealant	0.00	0.00	0.00
order	OPB54	control	0.00	2.20	241.20
order	Opitutales	cephalonium	0.00	0.00	0.00
order	Opitutales	cloxacillin	0.00	0.00	0.00

order	Opitutales	teat-sealant	0.00	456.80	0.20
order	Opitutales	control	0.00	0.00	0.00
order	Oscillatoriales	cephalonium	6.00	43.40	0.00
order	Oscillatoriales	cloxacillin	6.40	30.80	0.00
order	Oscillatoriales	teat-sealant	0.00	35.40	0.00
order	Oscillatoriales	control	0.00	23.20	0.00
order	Pasteurellales	cephalonium	22.80	21.80	78.20
order	Pasteurellales	cloxacillin	9.80	13.60	50.40
order	Pasteurellales	teat-sealant	61.20	49.80	145.20
order	Pasteurellales	control	286.60	0.60	65.00
order	Pirellulales	cephalonium	63.20	27.80	126.40
order	Pirellulales	cloxacillin	0.40	21.00	86.80
order	Pirellulales	teat-sealant	0.00	0.40	332.40
order	Pirellulales	control	99.40	4.00	0.20
order	Planctomycetales	cephalonium	0.20	0.60	251.40
order	Planctomycetales	cloxacillin	0.00	0.20	17.20
order	Planctomycetales	teat-sealant	0.00	0.00	0.00
order	Planctomycetales	control	0.00	0.00	0.00
order	Pseudomonadales	cephalonium	14396.60	22826.00	28084.20
order	Pseudomonadales	cloxacillin	11074.40	15171.80	32424.80
order	Pseudomonadales	teat-sealant	16087.80	23012.00	29547.80
order	Pseudomonadales	control	17020.20	22770.60	25798.40
order	RB41	cephalonium	0.00	1.40	0.00
order	RB41	cloxacillin	9.80	0.00	0.00
order	RB41	teat-sealant	0.00	0.00	0.00
order	RB41	control	0.00	24.20	26.40
order	RF32	cephalonium	0.00	0.00	0.00
order	RF32	cloxacillin	11.60	0.00	0.00
order	RF32	teat-sealant	9.40	0.00	0.00
order	RF32	control	6.20	0.60	0.00
order	RF39	cephalonium	12.00	1.20	1.00
order	RF39	cloxacillin	24.00	0.00	0.00
order	RF39	teat-sealant	12.00	0.00	0.20
order	RF39	control	13.60	1.80	8.80
order	Rhizobiales	cephalonium	841.60	2342.00	2317.00
order	Rhizobiales	cloxacillin	1405.60	1048.40	3033.00
order	Rhizobiales	teat-sealant	894.60	2705.20	2657.00
order	Rhizobiales	control	1215.20	1427.00	1976.00
order	Rhodobacterales	cephalonium	212.00	1081.20	198.00
order	Rhodobacterales	cloxacillin	514.40	305.60	749.20
order	Rhodobacterales	teat-sealant	81.80	446.80	820.80
order	Rhodobacterales	control	316.20	196.80	388.80
order	Rhodocyclales	cephalonium	0.00	102.20	77.40
order	Rhodocyclales	cloxacillin	4.80	0.20	20.40
order	Rhodocyclales	teat-sealant	0.40	18.60	13.40

order	Rhodocyclales	control	7.60	1.00	0.00
order	Rhodospirillales	cephalonium	12.60	463.60	623.20
order	Rhodospirillales	cloxacillin	61.60	178.40	991.60
order	Rhodospirillales	teat-sealant	48.00	316.20	190.60
order	Rhodospirillales	control	46.00	153.60	591.20
order	Rickettsiales	cephalonium	33.80	170.00	85.80
order	Rickettsiales	cloxacillin	24.00	83.80	258.00
order	Rickettsiales	teat-sealant	7.40	113.60	84.20
order	Rickettsiales	control	36.00	62.20	224.40
order	[Saprospirales]	cephalonium	63.20	150.20	548.60
order	[Saprospirales]	cloxacillin	32.00	85.80	224.60
order	[Saprospirales]	teat-sealant	92.80	811.80	559.20
order	[Saprospirales]	control	360.60	548.60	552.40
order	SBla14	cephalonium	0.00	0.00	0.00
order	SBla14	cloxacillin	11.40	5.60	0.00
order	SBla14	teat-sealant	0.00	0.20	0.20
order	SBla14	control	1.00	2.40	0.00
order	SHA-98	cephalonium	0.00	180.00	0.20
order	SHA-98	cloxacillin	0.00	0.00	0.00
order	SHA-98	teat-sealant	0.00	0.00	0.00
order	SHA-98	control	101.20	0.20	0.00
order	Solibacterales	cephalonium	17.40	0.20	63.20
order	Solibacterales	cloxacillin	3.20	0.40	6.20
order	Solibacterales	teat-sealant	2.20	7.80	196.80
order	Solibacterales	control	10.00	64.60	49.80
order	Solirubrobacterales	cephalonium	0.20	0.20	90.80
order	Solirubrobacterales	cloxacillin	0.20	13.00	0.00
order	Solirubrobacterales	teat-sealant	10.80	0.80	13.40
order	Solirubrobacterales	control	0.00	0.20	25.80
order	Sphingobacteriales	cephalonium	421.60	2590.60	563.00
order	Sphingobacteriales	cloxacillin	689.20	1061.20	1146.40
order	Sphingobacteriales	teat-sealant	318.00	28.80	734.00
order	Sphingobacteriales	control	164.60	564.40	103.60
order	Sphingomonadales	cephalonium	324.20	1018.20	188.40
order	Sphingomonadales	cloxacillin	608.00	576.20	524.60
order	Sphingomonadales	teat-sealant	180.60	411.60	533.80
order	Sphingomonadales	control	166.60	341.40	541.20
order	Spirochaetales	cephalonium	185.60	1.20	543.80
order	Spirochaetales	cloxacillin	81.20	0.00	74.40
order	Spirochaetales	teat-sealant	166.20	0.20	0.20
order	Spirochaetales	control	133.80	0.40	0.00
order	Streptophyta	cephalonium	90.60	1879.80	5073.20
order	Streptophyta	cloxacillin	129.60	1643.00	13005.00
order	Streptophyta	teat-sealant	129.80	2082.60	6169.60
order	Streptophyta	control	79.80	2230.60	4465.80

order	Thermales	cephalonium	0.00	0.00	0.00
order	Thermales	cloxacillin	9.60	0.20	258.40
order	Thermales	teat-sealant	61.00	4.40	0.00
order	Thermales	control	0.00	0.00	0.00
order	Thermotogales	cephalonium	25.60	131.40	0.00
order	Thermotogales	cloxacillin	0.20	464.80	0.60
order	Thermotogales	teat-sealant	0.20	0.20	108.80
order	Thermotogales	control	0.40	202.80	0.40
order	Turicibacterales	cephalonium	329.20	57.60	2.80
order	Turicibacterales	cloxacillin	706.60	4.40	15.40
order	Turicibacterales	teat-sealant	216.00	30.20	2.00
order	Turicibacterales	control	1963.40	147.20	1.60
order	Verrucomicrobiales	cephalonium	99.20	334.80	866.40
order	Verrucomicrobiales	cloxacillin	434.00	193.40	268.80
order	Verrucomicrobiales	teat-sealant	235.80	777.60	152.40
order	Verrucomicrobiales	control	244.80	142.20	384.40
order	Vibrionales	cephalonium	4.60	0.00	0.00
order	Vibrionales	cloxacillin	1.60	0.00	0.00
order	Vibrionales	teat-sealant	2.40	0.00	0.20
order	Vibrionales	control	1.00	0.00	1.00
order	Victivallales	cephalonium	1.20	0.00	0.00
order	Victivallales	cloxacillin	10.00	0.00	0.00
order	Victivallales	teat-sealant	8.40	0.00	0.00
order	Victivallales	control	0.00	0.00	0.00
order	WCHB1-15	cephalonium	0.00	0.40	140.20
order	WCHB1-15	cloxacillin	0.00	0.00	0.00
order	WCHB1-15	teat-sealant	0.00	0.00	0.00
order	WCHB1-15	control	0.00	0.00	0.00
order	WCHB1-41	cephalonium	15.00	0.20	0.00
order	WCHB1-41	cloxacillin	46.60	0.00	0.00
order	WCHB1-41	teat-sealant	25.60	0.40	0.00
order	WCHB1-41	control	11.60	7.20	0.00
order	WD2101	cephalonium	0.00	0.00	33.40
order	WD2101	cloxacillin	0.00	0.00	0.00
order	WD2101	teat-sealant	0.00	0.00	0.00
order	WD2101	control	0.00	79.40	0.00
order	Xanthomonadales	cephalonium	367.60	2341.40	358.20
order	Xanthomonadales	cloxacillin	963.80	1185.80	1327.40
order	Xanthomonadales	teat-sealant	622.20	661.80	577.60
order	Xanthomonadales	control	443.60	757.20	358.40
order	YS2	cephalonium	40.20	70.80	0.00
order	YS2	cloxacillin	252.20	0.40	0.00
order	YS2	teat-sealant	148.80	0.60	0.20
order	YS2	control	34.80	29.80	0.00
family	Acetobacteraceae	cephalonium	9.00	0.40	19.20

family	Acetobacteraceae	cloxacillin	30.60	14.60	1.00
family	Acetobacteraceae	teat-sealant	1.40	0.00	0.00
family	Acetobacteraceae	control	1.80	26.20	5.00
family	Acholeplasmataceae	cephalonium	12.20	0.00	6.20
family	Acholeplasmataceae	cloxacillin	40.40	0.40	17.00
family	Acholeplasmataceae	teat-sealant	6.60	0.00	0.00
family	Acholeplasmataceae	control	9.40	0.20	0.00
family	[Acidaminobacteraceae]	cephalonium	39.60	193.40	0.00
family	[Acidaminobacteraceae]	cloxacillin	16.00	0.20	0.00
family	[Acidaminobacteraceae]	teat-sealant	5.60	18.60	17.20
family	[Acidaminobacteraceae]	control	8.60	0.00	1.80
family	ACK-M1	cephalonium	0.00	0.00	0.00
family	ACK-M1	cloxacillin	0.00	0.00	0.00
family	ACK-M1	teat-sealant	0.00	0.00	0.00
family	ACK-M1	control	0.00	0.20	173.40
family	Actinomycetaceae	cephalonium	98.00	128.40	131.60
family	Actinomycetaceae	cloxacillin	36.00	44.60	289.20
family	Actinomycetaceae	teat-sealant	57.20	19.00	129.80
family	Actinomycetaceae	control	212.20	184.40	35.00
family	Actinopolysporaceae	cephalonium	0.40	0.00	0.00
family	Actinopolysporaceae	cloxacillin	0.00	0.00	29.40
family	Actinopolysporaceae	teat-sealant	0.00	23.20	0.00
family	Actinopolysporaceae	control	112.40	0.20	0.00
family	Actinosynnemataceae	cephalonium	0.80	2.60	0.20
family	Actinosynnemataceae	cloxacillin	0.00	0.20	1.80
family	Actinosynnemataceae	teat-sealant	0.20	1.00	1.20
family	Actinosynnemataceae	control	0.80	0.80	0.00
family	Aerococcaceae	cephalonium	1430.00	2158.00	1849.40
family	Aerococcaceae	cloxacillin	2297.60	822.20	1567.80
family	Aerococcaceae	teat-sealant	1297.40	277.20	3072.40
family	Aerococcaceae	control	814.40	646.80	423.80
family	Aeromonadaceae	cephalonium	7.80	0.00	0.00
family	Aeromonadaceae	cloxacillin	0.60	2.40	13.00
family	Aeromonadaceae	teat-sealant	0.00	0.00	2.20
family	Aeromonadaceae	control	0.00	0.00	0.20
family	Alcaligenaceae	cephalonium	2251.00	3231.60	4780.80
family	Alcaligenaceae	cloxacillin	1507.20	2265.00	4010.60
family	Alcaligenaceae	teat-sealant	2311.80	3303.60	5802.40
family	Alcaligenaceae	control	2483.00	2513.40	3697.40
family	Alcanivoracaceae	cephalonium	32.40	129.40	89.80
family	Alcanivoracaceae	cloxacillin	24.80	9.00	115.00
family	Alcanivoracaceae	teat-sealant	47.80	133.20	64.60
family	Alcanivoracaceae	control	102.40	55.00	104.20
family	Alteromonadaceae	cephalonium	88.20	1021.80	57.80
family	Alteromonadaceae	cloxacillin	149.40	113.60	264.80

family	Alteromonadaceae	teat-sealant	16.20	1.00	94.40
family	Alteromonadaceae	control	60.60	53.60	33.60
family	Anaeroplasmataceae	cephalonium	0.00	0.00	0.00
family	Anaeroplasmataceae	cloxacillin	44.20	0.00	0.00
family	Anaeroplasmataceae	teat-sealant	11.40	0.00	0.00
family	Anaeroplasmataceae	control	12.00	0.20	0.00
family	Aurantimonadaceae	cephalonium	14.80	12.00	0.40
family	Aurantimonadaceae	cloxacillin	46.20	24.80	33.20
family	Aurantimonadaceae	teat-sealant	26.00	94.60	9.40
family	Aurantimonadaceae	control	23.80	44.00	14.80
family	Bacillaceae	cephalonium	254.40	807.80	117.20
family	Bacillaceae	cloxacillin	298.20	453.80	279.20
family	Bacillaceae	teat-sealant	171.80	7.60	565.40
family	Bacillaceae	control	307.00	75.40	74.80
family	Bacteroidaceae	cephalonium	1429.60	691.40	313.20
family	Bacteroidaceae	cloxacillin	1760.40	100.80	68.40
family	Bacteroidaceae	teat-sealant	753.20	1.20	365.20
family	Bacteroidaceae	control	1101.60	523.80	231.20
family	[Barnesiellaceae]	cephalonium	0.00	0.00	0.00
family	[Barnesiellaceae]	cloxacillin	8.60	0.00	0.00
family	[Barnesiellaceae]	teat-sealant	0.00	0.00	0.00
family	[Barnesiellaceae]	control	0.00	14.60	0.00
family	Bartonellaceae	cephalonium	0.80	3.20	1.80
family	Bartonellaceae	cloxacillin	2.40	2.40	2.60
family	Bartonellaceae	teat-sealant	4.00	152.80	3.00
family	Bartonellaceae	control	2.00	1.00	1.40
family	Beijerinckiaceae	cephalonium	2.60	5.20	29.60
family	Beijerinckiaceae	cloxacillin	6.40	3.80	10.80
family	Beijerinckiaceae	teat-sealant	37.80	25.60	14.40
family	Beijerinckiaceae	control	16.80	3.00	19.80
family	Beutenbergiaceae	cephalonium	1.60	30.80	1.20
family	Beutenbergiaceae	cloxacillin	10.80	11.60	8.20
family	Beutenbergiaceae	teat-sealant	4.20	0.00	6.20
family	Beutenbergiaceae	control	2.00	4.40	0.00
family	Bifidobacteriaceae	cephalonium	171.20	122.40	178.00
family	Bifidobacteriaceae	cloxacillin	802.40	122.40	68.80
family	Bifidobacteriaceae	teat-sealant	122.80	152.60	21.60
family	Bifidobacteriaceae	control	268.20	1136.60	0.20
family	Bogoriellaceae	cephalonium	6.60	40.00	7.20
family	Bogoriellaceae	cloxacillin	15.20	26.80	24.00
family	Bogoriellaceae	teat-sealant	14.00	0.00	60.60
family	Bogoriellaceae	control	21.60	1.40	0.00
family	Bradyrhizobiaceae	cephalonium	463.80	864.00	971.20
family	Bradyrhizobiaceae	cloxacillin	399.00	261.80	1169.40
family	Bradyrhizobiaceae	teat-sealant	456.80	1438.40	653.20

family	Bradyrhizobiaceae	control	770.60	350.60	848.00
family	Brevibacteriaceae	cephalonium	95.80	619.00	416.60
family	Brevibacteriaceae	cloxacillin	412.60	230.60	849.00
family	Brevibacteriaceae	teat-sealant	415.00	42.80	1552.60
family	Brevibacteriaceae	control	84.00	150.20	113.00
family	Brucellaceae	cephalonium	7.60	32.80	89.60
family	Brucellaceae	cloxacillin	4.40	7.60	17.00
family	Brucellaceae	teat-sealant	3.80	10.20	7.40
family	Brucellaceae	control	0.40	1.20	0.60
family	BS11	cephalonium	32.00	0.20	0.00
family	BS11	cloxacillin	40.40	0.00	0.00
family	BS11	teat-sealant	87.20	0.00	0.40
family	BS11	control	32.20	2.00	0.20
family	C111	cephalonium	5.60	0.00	0.00
family	C111	cloxacillin	3.80	0.00	0.00
family	C111	teat-sealant	0.00	61.40	40.40
family	C111	control	0.00	0.00	0.00
family	Caldicoprobacteraceae	cephalonium	0.00	0.20	7.00
family	Caldicoprobacteraceae	cloxacillin	0.00	0.00	0.00
family	Caldicoprobacteraceae	teat-sealant	0.00	0.00	0.00
family	Caldicoprobacteraceae	control	0.00	0.00	0.00
family	Caldilineaceae	cephalonium	0.20	189.60	0.00
family	Caldilineaceae	cloxacillin	0.00	0.00	258.20
family	Caldilineaceae	teat-sealant	0.20	0.20	156.80
family	Caldilineaceae	control	0.00	0.20	0.00
family	Campylobacteraceae	cephalonium	64.20	0.00	0.20
family	Campylobacteraceae	cloxacillin	107.00	25.80	169.40
family	Campylobacteraceae	teat-sealant	80.40	3.80	0.00
family	Campylobacteraceae	control	57.60	50.00	0.20
family	Cardiobacteriaceae	cephalonium	18.40	0.00	0.00
family	Cardiobacteriaceae	cloxacillin	0.40	0.20	198.00
family	Cardiobacteriaceae	teat-sealant	0.00	0.00	0.00
family	Cardiobacteriaceae	control	0.00	0.00	0.00
family	Carnobacteriaceae	cephalonium	198.80	247.00	562.20
family	Carnobacteriaceae	cloxacillin	248.40	25.80	421.20
family	Carnobacteriaceae	teat-sealant	104.20	20.60	266.60
family	Carnobacteriaceae	control	90.00	86.80	165.60
family	Caulobacteraceae	cephalonium	215.80	521.00	141.80
family	Caulobacteraceae	cloxacillin	120.80	198.80	108.20
family	Caulobacteraceae	teat-sealant	100.20	277.40	257.20
family	Caulobacteraceae	control	147.00	135.20	233.60
family	Cellulomonadaceae	cephalonium	105.40	261.60	84.20
family	Cellulomonadaceae	cloxacillin	128.40	76.40	118.00
family	Cellulomonadaceae	teat-sealant	43.60	52.20	549.00
family	Cellulomonadaceae	control	61.20	32.80	146.20

family	Chitinophagaceae	cephalonium	63.00	150.00	352.80
family	Chitinophagaceae	cloxacillin	31.60	85.80	219.80
family	Chitinophagaceae	teat-sealant	92.80	620.80	559.20
family	Chitinophagaceae	control	360.60	362.40	507.80
family	Christensenellaceae	cephalonium	5.40	23.80	0.00
family	Christensenellaceae	cloxacillin	41.20	3.20	0.00
family	Christensenellaceae	teat-sealant	1.80	27.00	0.00
family	Christensenellaceae	control	24.80	6.00	0.00
family	[Chromatiaceae]	cephalonium	0.00	0.00	13.00
family	[Chromatiaceae]	cloxacillin	6.20	0.00	0.00
family	[Chromatiaceae]	teat-sealant	1.20	1.20	0.00
family	[Chromatiaceae]	control	4.20	3.80	0.00
family	[Chthoniobacteraceae]	cephalonium	0.00	0.00	0.00
family	[Chthoniobacteraceae]	cloxacillin	21.00	0.00	88.80
family	[Chthoniobacteraceae]	teat-sealant	0.00	0.00	0.00
family	[Chthoniobacteraceae]	control	0.00	107.20	0.00
family	Clostridiaceae	cephalonium	1039.40	1284.00	342.20
family	Clostridiaceae	cloxacillin	3900.20	453.60	261.40
family	Clostridiaceae	teat-sealant	854.40	414.20	476.20
family	Clostridiaceae	control	1166.60	685.60	457.20
family	Comamonadaceae	cephalonium	265.40	1526.40	1111.60
family	Comamonadaceae	cloxacillin	880.40	514.20	920.00
family	Comamonadaceae	teat-sealant	222.80	1425.60	549.80
family	Comamonadaceae	control	290.20	560.80	1208.40
family	Conexibacteraceae	cephalonium	0.00	0.20	8.80
family	Conexibacteraceae	cloxacillin	0.00	11.40	0.00
family	Conexibacteraceae	teat-sealant	10.40	0.20	0.00
family	Conexibacteraceae	control	0.00	0.20	14.00
family	Coriobacteriaceae	cephalonium	46.40	367.60	0.20
family	Coriobacteriaceae	cloxacillin	146.40	26.40	417.20
family	Coriobacteriaceae	teat-sealant	59.20	0.20	60.80
family	Coriobacteriaceae	control	55.40	36.80	157.80
family	Corynebacteriaceae	cephalonium	2041.80	2544.00	1946.40
family	Corynebacteriaceae	cloxacillin	3878.60	1230.40	1432.40
family	Corynebacteriaceae	teat-sealant	1926.20	1073.00	2556.60
family	Corynebacteriaceae	control	1651.40	1155.40	850.20
family	Coxiellaceae	cephalonium	297.80	2.60	46.00
family	Coxiellaceae	cloxacillin	80.80	245.20	3.20
family	Coxiellaceae	teat-sealant	260.20	553.80	91.20
family	Coxiellaceae	control	3.00	642.40	1.00
family	Cryomorphaceae	cephalonium	12.20	101.80	4.00
family	Cryomorphaceae	cloxacillin	50.00	129.80	350.80
family	Cryomorphaceae	teat-sealant	35.80	50.00	737.00
family	Cryomorphaceae	control	1.00	314.00	600.40
family	Cryptosporangiaceae	cephalonium	0.60	4.20	0.20

family	Cryptosporangiaceae	cloxacillin	0.20	1.40	3.00
family	Cryptosporangiaceae	teat-sealant	0.00	1.20	1.60
family	Cryptosporangiaceae	control	0.80	1.00	0.20
family	Cyclobacteriaceae	cephalonium	18.40	223.80	1.00
family	Cyclobacteriaceae	cloxacillin	70.40	126.00	9.20
family	Cyclobacteriaceae	teat-sealant	9.40	7.80	80.40
family	Cyclobacteriaceae	control	12.60	170.40	156.80
family	Cytophagaceae	cephalonium	194.40	414.40	181.60
family	Cytophagaceae	cloxacillin	1819.00	105.60	596.00
family	Cytophagaceae	teat-sealant	33.80	154.40	71.20
family	Cytophagaceae	control	98.80	3.80	27.40
family	Deinococcaceae	cephalonium	62.00	0.00	0.00
family	Deinococcaceae	cloxacillin	233.40	0.40	0.20
family	Deinococcaceae	teat-sealant	140.40	1.80	0.20
family	Deinococcaceae	control	64.00	0.00	53.20
family	Dermabacteraceae	cephalonium	36.40	101.20	99.00
family	Dermabacteraceae	cloxacillin	95.60	20.00	308.80
family	Dermabacteraceae	teat-sealant	215.00	8.00	756.20
family	Dermabacteraceae	control	28.40	28.80	4.00
family	Dermacoccaceae	cephalonium	3.00	34.20	0.00
family	Dermacoccaceae	cloxacillin	2.20	2.20	59.80
family	Dermacoccaceae	teat-sealant	2.80	0.00	84.80
family	Dermacoccaceae	control	0.60	0.00	0.00
family	Dermatophilaceae	cephalonium	0.60	0.00	5.20
family	Dermatophilaceae	cloxacillin	0.00	0.00	0.00
family	Dermatophilaceae	teat-sealant	0.00	0.00	0.00
family	Dermatophilaceae	control	0.80	0.00	0.00
family	Desulfovibrionaceae	cephalonium	4.00	95.20	13.20
family	Desulfovibrionaceae	cloxacillin	33.40	0.00	3.00
family	Desulfovibrionaceae	teat-sealant	25.80	0.00	1.20
family	Desulfovibrionaceae	control	46.00	0.00	0.00
family	Dietziaceae	cephalonium	127.40	157.60	282.00
family	Dietziaceae	cloxacillin	365.00	91.20	227.00
family	Dietziaceae	teat-sealant	116.00	61.80	201.80
family	Dietziaceae	control	100.40	4.20	64.20
family	Ellin6075	cephalonium	0.00	1.40	0.00
family	Ellin6075	cloxacillin	9.80	0.00	0.00
family	Ellin6075	teat-sealant	0.00	0.00	0.00
family	Ellin6075	control	0.00	24.20	26.40
family	Elusimicrobiaceae	cephalonium	0.00	32.60	0.00
family	Elusimicrobiaceae	cloxacillin	1.00	0.00	0.00
family	Elusimicrobiaceae	teat-sealant	0.00	0.00	0.00
family	Elusimicrobiaceae	control	0.00	0.00	0.00
family	Enterobacteriaceae	cephalonium	71.20	217.00	80.80
family	Enterobacteriaceae	cloxacillin	119.00	111.00	136.00

family	Enterobacteriaceae	teat-sealant	688.80	86.60	94.80
family	Enterobacteriaceae	control	45.60	18906.80	377.60
family	Enterococcaceae	cephalonium	43.20	508.80	24.60
family	Enterococcaceae	cloxacillin	104.60	1.00	198.80
family	Enterococcaceae	teat-sealant	0.40	5.20	479.60
family	Enterococcaceae	control	7.80	291.40	0.60
family	Erysipelotrichaceae	cephalonium	49.20	66.00	157.80
family	Erysipelotrichaceae	cloxacillin	223.40	3.40	53.80
family	Erysipelotrichaceae	teat-sealant	21.60	0.00	0.00
family	Erysipelotrichaceae	control	31.60	14.40	263.20
family	Erythrobacteraceae	cephalonium	21.20	174.80	7.80
family	Erythrobacteraceae	cloxacillin	39.40	44.20	27.60
family	Erythrobacteraceae	teat-sealant	12.60	5.80	10.00
family	Erythrobacteraceae	control	8.20	35.40	6.40
family	Eubacteriaceae	cephalonium	2.80	18.80	0.00
family	Eubacteriaceae	cloxacillin	10.00	0.00	0.00
family	Eubacteriaceae	teat-sealant	0.00	0.00	0.00
family	Eubacteriaceae	control	78.80	8.40	0.20
family	[Exiguobacteraceae]	cephalonium	7.60	0.00	0.00
family	[Exiguobacteraceae]	cloxacillin	2.20	0.20	41.60
family	[Exiguobacteraceae]	teat-sealant	0.00	0.40	0.00
family	[Exiguobacteraceae]	control	28.60	0.20	0.20
family	F16	cephalonium	51.40	390.40	61.40
family	F16	cloxacillin	401.60	0.00	6.80
family	F16	teat-sealant	110.20	0.20	0.00
family	F16	control	15.20	0.60	0.00
family	[Fimbriimonadaceae]	cephalonium	0.00	0.00	0.00
family	[Fimbriimonadaceae]	cloxacillin	0.00	0.00	0.00
family	[Fimbriimonadaceae]	teat-sealant	0.00	0.40	76.80
family	[Fimbriimonadaceae]	control	0.00	0.00	0.00
family	Flammeovirgaceae	cephalonium	14.60	30.00	0.00
family	Flammeovirgaceae	cloxacillin	0.00	0.00	0.00
family	Flammeovirgaceae	teat-sealant	0.00	0.00	5.60
family	Flammeovirgaceae	control	0.00	0.00	0.00
family	Flavobacteriaceae	cephalonium	758.40	4094.00	964.40
family	Flavobacteriaceae	cloxacillin	941.40	1287.20	2333.40
family	Flavobacteriaceae	teat-sealant	317.80	66.00	1531.00
family	Flavobacteriaceae	control	436.20	596.60	294.80
family	Frankiaceae	cephalonium	22.80	141.40	8.80
family	Frankiaceae	cloxacillin	6.20	21.60	87.40
family	Frankiaceae	teat-sealant	15.20	85.60	133.00
family	Frankiaceae	control	30.20	19.00	8.20
family	Fusobacteriaceae	cephalonium	9.60	0.20	86.00
family	Fusobacteriaceae	cloxacillin	6.60	0.00	7.20
family	Fusobacteriaceae	teat-sealant	26.00	2.00	98.40

family	Fusobacteriaceae	control	0.40	50.40	7.60
family	Gaiellaceae	cephalonium	1.20	1.60	1.00
family	Gaiellaceae	cloxacillin	18.80	6.40	2.80
family	Gaiellaceae	teat-sealant	0.40	70.40	20.40
family	Gaiellaceae	control	5.00	13.60	18.20
family	Gemellaceae	cephalonium	4.80	0.00	0.00
family	Gemellaceae	cloxacillin	0.60	51.00	10.60
family	Gemellaceae	teat-sealant	0.00	0.00	0.00
family	Gemellaceae	control	0.00	0.00	0.00
family	Gemmataceae	cephalonium	98.00	471.80	86.00
family	Gemmataceae	cloxacillin	17.40	51.20	1238.60
family	Gemmataceae	teat-sealant	28.60	278.20	471.60
family	Gemmataceae	control	371.80	128.00	556.60
family	Geodermatophilaceae	cephalonium	0.00	0.00	1.00
family	Geodermatophilaceae	cloxacillin	17.00	88.00	37.40
family	Geodermatophilaceae	teat-sealant	0.00	0.40	4.60
family	Geodermatophilaceae	control	0.00	4.60	0.00
family	Gordoniaceae	cephalonium	2.40	0.00	2.80
family	Gordoniaceae	cloxacillin	2.00	0.00	1.20
family	Gordoniaceae	teat-sealant	1.80	0.00	4.80
family	Gordoniaceae	control	0.00	0.20	0.00
family	GZKB119	cephalonium	0.00	0.00	0.00
family	GZKB119	cloxacillin	0.00	101.80	0.40
family	GZKB119	teat-sealant	0.00	0.00	0.00
family	GZKB119	control	0.00	0.00	0.00
family	Halanaerobiaceae	cephalonium	0.00	1.60	0.00
family	Halanaerobiaceae	cloxacillin	0.00	0.00	0.00
family	Halanaerobiaceae	teat-sealant	0.00	0.00	0.00
family	Halanaerobiaceae	control	1.00	348.40	0.40
family	Halomonadaceae	cephalonium	31.00	433.00	27.60
family	Halomonadaceae	cloxacillin	43.20	251.40	33.60
family	Halomonadaceae	teat-sealant	2.80	56.40	0.40
family	Halomonadaceae	control	13.40	25.20	0.00
family	HTCC2188	cephalonium	0.60	3.00	0.00
family	HTCC2188	cloxacillin	0.00	0.00	0.00
family	HTCC2188	teat-sealant	0.00	0.00	0.00
family	HTCC2188	control	0.20	0.00	0.00
family	Hyphomicrobiaceae	cephalonium	35.80	231.00	74.40
family	Hyphomicrobiaceae	cloxacillin	233.20	163.40	265.60
family	Hyphomicrobiaceae	teat-sealant	69.80	157.00	135.20
family	Hyphomicrobiaceae	control	48.00	390.80	66.20
family	Idiomarinaceae	cephalonium	2.00	23.60	18.60
family	Idiomarinaceae	cloxacillin	13.00	8.60	0.40
family	Idiomarinaceae	teat-sealant	2.60	0.00	26.40
family	Idiomarinaceae	control	7.00	0.00	0.20

family	Intrasporangiaceae	cephalonium	161.00	54.80	18.60
family	Intrasporangiaceae	cloxacillin	152.40	44.80	118.00
family	Intrasporangiaceae	teat-sealant	179.20	7.20	1192.80
family	Intrasporangiaceae	control	116.20	55.00	5.40
family	Isosphaeraceae	cephalonium	2.20	826.40	1431.00
family	Isosphaeraceae	cloxacillin	12.00	647.00	551.60
family	Isosphaeraceae	teat-sealant	85.40	1147.40	463.80
family	Isosphaeraceae	control	1.00	707.80	650.60
family	Jonesiaceae	cephalonium	56.60	57.80	5.80
family	Jonesiaceae	cloxacillin	34.60	7.60	9.40
family	Jonesiaceae	teat-sealant	28.80	0.40	0.60
family	Jonesiaceae	control	19.20	4.40	0.20
family	Kineosporiaceae	cephalonium	4.20	12.00	0.00
family	Kineosporiaceae	cloxacillin	1.00	9.00	5.80
family	Kineosporiaceae	teat-sealant	2.80	0.60	0.00
family	Kineosporiaceae	control	0.00	0.00	0.00
family	Lachnospiraceae	cephalonium	1976.20	616.40	828.00
family	Lachnospiraceae	cloxacillin	4094.40	185.60	460.00
family	Lachnospiraceae	teat-sealant	1694.60	201.20	348.80
family	Lachnospiraceae	control	1730.80	308.60	164.60
family	Lactobacillaceae	cephalonium	2606.20	4172.40	7348.00
family	Lactobacillaceae	cloxacillin	2588.60	4037.20	5043.80
family	Lactobacillaceae	teat-sealant	3237.80	5660.80	8126.00
family	Lactobacillaceae	control	2735.00	4710.00	7590.40
family	Leptotrichiaceae	cephalonium	0.20	126.20	0.00
family	Leptotrichiaceae	cloxacillin	0.00	0.00	0.00
family	Leptotrichiaceae	teat-sealant	18.40	0.00	6.00
family	Leptotrichiaceae	control	0.00	0.00	0.00
family	Leuconostocaceae	cephalonium	0.20	243.80	516.00
family	Leuconostocaceae	cloxacillin	35.40	289.80	246.00
family	Leuconostocaceae	teat-sealant	0.20	0.00	242.20
family	Leuconostocaceae	control	0.40	75.60	0.40
family	Listeriaceae	cephalonium	0.20	0.00	0.00
family	Listeriaceae	cloxacillin	0.00	0.00	0.20
family	Listeriaceae	teat-sealant	90.40	0.00	0.00
family	Listeriaceae	control	0.00	0.20	120.60
family	Marinilabiaceae	cephalonium	4.00	0.00	0.00
family	Marinilabiaceae	cloxacillin	2.60	3.00	0.00
family	Marinilabiaceae	teat-sealant	0.00	0.00	0.00
family	Marinilabiaceae	control	0.20	0.00	0.00
family	Methanobacteriaceae	cephalonium	252.80	43.20	20.40
family	Methanobacteriaceae	cloxacillin	441.60	337.80	45.00
family	Methanobacteriaceae	teat-sealant	103.00	35.40	44.60
family	Methanobacteriaceae	control	130.20	43.80	23.40
family	Methanosarcinaceae	cephalonium	0.00	0.00	0.00

family	Methanosarcinaceae	cloxacillin	1.40	0.00	0.00
family	Methanosarcinaceae	teat-sealant	0.00	0.00	0.00
family	Methanosarcinaceae	control	2.00	0.00	0.00
family	Methylobacteriaceae	cephalonium	106.80	138.40	30.00
family	Methylobacteriaceae	cloxacillin	212.80	93.80	203.40
family	Methylobacteriaceae	teat-sealant	73.00	166.60	491.40
family	Methylobacteriaceae	control	56.40	53.60	265.60
family	Methylocystaceae	cephalonium	0.40	4.60	2.40
family	Methylocystaceae	cloxacillin	1.80	1.40	1.80
family	Methylocystaceae	teat-sealant	0.80	1.40	2.00
family	Methylocystaceae	control	1.80	6.00	0.40
family	Methylophilaceae	cephalonium	0.00	0.00	0.00
family	Methylophilaceae	cloxacillin	0.00	0.00	193.00
family	Methylophilaceae	teat-sealant	0.00	156.60	0.20
family	Methylophilaceae	control	0.00	0.60	475.80
family	Microbacteriaceae	cephalonium	275.60	1417.40	387.80
family	Microbacteriaceae	cloxacillin	729.60	670.40	430.60
family	Microbacteriaceae	teat-sealant	359.20	216.20	321.40
family	Microbacteriaceae	control	263.60	195.60	927.00
family	Micrococcaceae	cephalonium	814.60	2622.80	989.00
family	Micrococcaceae	cloxacillin	1100.00	731.20	1046.00
family	Micrococcaceae	teat-sealant	884.00	608.80	1119.80
family	Micrococcaceae	control	415.60	309.00	305.20
family	Micromonosporaceae	cephalonium	0.20	30.40	0.40
family	Micromonosporaceae	cloxacillin	2.40	5.80	0.80
family	Micromonosporaceae	teat-sealant	0.20	10.60	0.20
family	Micromonosporaceae	control	17.80	0.40	0.60
family	mitochondria	cephalonium	3.40	169.60	85.80
family	mitochondria	cloxacillin	24.00	81.80	133.60
family	mitochondria	teat-sealant	4.60	52.80	44.20
family	mitochondria	control	3.20	19.80	224.40
family	ML635J-40	cephalonium	0.00	0.00	0.00
family	ML635J-40	cloxacillin	8.80	0.00	0.20
family	ML635J-40	teat-sealant	0.00	0.00	0.00
family	ML635J-40	control	0.00	0.00	0.00
family	[Mogibacteriaceae]	cephalonium	193.40	124.40	18.40
family	[Mogibacteriaceae]	cloxacillin	275.80	7.80	40.20
family	[Mogibacteriaceae]	teat-sealant	57.40	0.60	88.00
family	[Mogibacteriaceae]	control	64.80	152.60	244.20
family	Moraxellaceae	cephalonium	1932.80	3976.40	5408.60
family	Moraxellaceae	cloxacillin	2909.00	2222.80	9291.80
family	Moraxellaceae	teat-sealant	1606.80	1695.80	4959.80
family	Moraxellaceae	control	2532.60	2338.80	4779.20
family	Mycobacteriaceae	cephalonium	7.20	2.20	4.40
family	Mycobacteriaceae	cloxacillin	0.00	0.00	59.40

family	Mycobacteriaceae	teat-sealant	1.80	26.00	30.20
family	Mycobacteriaceae	control	0.00	26.00	0.00
family	Nakamurellaceae	cephalonium	0.00	0.00	1.00
family	Nakamurellaceae	cloxacillin	3.20	0.00	0.00
family	Nakamurellaceae	teat-sealant	0.00	0.00	0.00
family	Nakamurellaceae	control	0.00	0.00	0.00
family	Neisseriaceae	cephalonium	31.80	234.80	31.00
family	Neisseriaceae	cloxacillin	72.60	51.60	297.80
family	Neisseriaceae	teat-sealant	126.20	424.00	127.20
family	Neisseriaceae	control	176.00	58.60	68.20
family	Nocardiaceae	cephalonium	22.80	9.40	8.60
family	Nocardiaceae	cloxacillin	10.40	8.80	64.20
family	Nocardiaceae	teat-sealant	9.80	1.20	13.00
family	Nocardiaceae	control	2.40	10.40	1.20
family	Nocardioidaceae	cephalonium	87.60	285.00	18.60
family	Nocardioidaceae	cloxacillin	178.80	26.00	29.20
family	Nocardioidaceae	teat-sealant	14.40	11.00	59.20
family	Nocardioidaceae	control	90.00	102.60	4.00
family	Nocardiopsaceae	cephalonium	0.80	2.00	0.00
family	Nocardiopsaceae	cloxacillin	13.80	0.00	0.00
family	Nocardiopsaceae	teat-sealant	0.00	0.00	2.80
family	Nocardiopsaceae	control	0.00	0.00	9.40
family	[Odoribacteraceae]	cephalonium	0.00	0.00	0.00
family	[Odoribacteraceae]	cloxacillin	1.20	18.60	0.00
family	[Odoribacteraceae]	teat-sealant	0.00	0.00	0.00
family	[Odoribacteraceae]	control	13.60	0.20	0.00
family	Opitutaceae	cephalonium	0.00	0.00	0.00
family	Opitutaceae	cloxacillin	0.00	0.00	0.00
family	Opitutaceae	teat-sealant	0.00	456.80	0.20
family	Opitutaceae	control	0.00	0.00	0.00
family	Oxalobacteraceae	cephalonium	60.80	116.40	448.60
family	Oxalobacteraceae	cloxacillin	13.40	40.20	166.20
family	Oxalobacteraceae	teat-sealant	99.60	145.80	28.80
family	Oxalobacteraceae	control	25.60	37.00	225.20
family	p-2534-18B5	cephalonium	7.40	0.00	0.00
family	p-2534-18B5	cloxacillin	13.00	0.00	0.40
family	p-2534-18B5	teat-sealant	16.40	0.20	0.00
family	p-2534-18B5	control	26.40	5.40	0.20
family	Paenibacillaceae	cephalonium	16.20	44.60	0.00
family	Paenibacillaceae	cloxacillin	11.80	27.20	28.20
family	Paenibacillaceae	teat-sealant	1.20	0.00	2.60
family	Paenibacillaceae	control	15.80	11.00	5.20
family	[Paraprevotellaceae]	cephalonium	241.20	50.40	159.20
family	[Paraprevotellaceae]	cloxacillin	1241.40	173.60	25.80
family	[Paraprevotellaceae]	teat-sealant	85.00	0.20	11.40

family	[Paraprevotellaceae]	control	373.20	23.20	25.00
family	Pasteurellaceae	cephalonium	22.80	21.80	78.20
family	Pasteurellaceae	cloxacillin	9.80	13.60	50.40
family	Pasteurellaceae	teat-sealant	61.20	49.80	145.20
family	Pasteurellaceae	control	286.60	0.60	65.00
family	Peptococcaceae	cephalonium	39.20	23.00	0.00
family	Peptococcaceae	cloxacillin	133.00	0.20	0.20
family	Peptococcaceae	teat-sealant	30.80	0.40	19.80
family	Peptococcaceae	control	100.40	7.00	0.00
family	Peptostreptococcaceae	cephalonium	511.40	127.00	263.80
family	Peptostreptococcaceae	cloxacillin	809.00	80.00	49.80
family	Peptostreptococcaceae	teat-sealant	367.80	75.80	200.80
family	Peptostreptococcaceae	control	155.20	82.20	62.40
family	Phormidiaceae	cephalonium	6.00	43.40	0.00
family	Phormidiaceae	cloxacillin	6.40	30.80	0.00
family	Phormidiaceae	teat-sealant	0.00	35.40	0.00
family	Phormidiaceae	control	0.00	23.20	0.00
family	Phyllobacteriaceae	cephalonium	76.80	354.60	226.80
family	Phyllobacteriaceae	cloxacillin	173.00	158.80	231.40
family	Phyllobacteriaceae	teat-sealant	52.60	143.40	210.80
family	Phyllobacteriaceae	control	147.00	48.60	218.20
family	Pirellulaceae	cephalonium	63.20	27.80	126.40
family	Pirellulaceae	cloxacillin	0.40	21.00	86.80
family	Pirellulaceae	teat-sealant	0.00	0.40	332.40
family	Pirellulaceae	control	99.40	4.00	0.20
family	Planctomycetaceae	cephalonium	0.20	0.60	251.40
family	Planctomycetaceae	cloxacillin	0.00	0.20	17.20
family	Planctomycetaceae	teat-sealant	0.00	0.00	0.00
family	Planctomycetaceae	control	0.00	0.00	0.00
family	Planococcaceae	cephalonium	1545.60	1663.20	965.80
family	Planococcaceae	cloxacillin	2844.00	631.80	1028.80
family	Planococcaceae	teat-sealant	983.80	127.80	972.20
family	Planococcaceae	control	866.40	156.60	172.40
family	Porphyromonadaceae	cephalonium	32.00	14.20	111.00
family	Porphyromonadaceae	cloxacillin	53.00	23.60	399.60
family	Porphyromonadaceae	teat-sealant	45.40	4.00	20.40
family	Porphyromonadaceae	control	60.40	9.40	0.60
family	Prevotellaceae	cephalonium	59.80	95.60	154.60
family	Prevotellaceae	cloxacillin	136.80	20.40	51.20
family	Prevotellaceae	teat-sealant	13.00	3.20	37.60
family	Prevotellaceae	control	204.20	17.80	0.80
family	Promicromonosporaceae	cephalonium	3.80	0.80	1.60
family	Promicromonosporaceae	cloxacillin	38.20	1.20	24.00
family	Promicromonosporaceae	teat-sealant	0.00	0.20	0.60
family	Promicromonosporaceae	control	8.60	0.20	158.80

family	Propionibacteriaceae	cephalonium	4501.20	8336.60	4381.60
family	Propionibacteriaceae	cloxacillin	3248.00	5381.20	6265.60
family	Propionibacteriaceae	teat-sealant	5374.00	9244.80	8270.20
family	Propionibacteriaceae	control	6953.20	6848.60	5833.00
family	Pseudoalteromonadaceae	cephalonium	0.00	0.00	0.00
family	Pseudoalteromonadaceae	cloxacillin	0.00	0.00	0.00
family	Pseudoalteromonadaceae	teat-sealant	2.40	0.00	0.20
family	Pseudoalteromonadaceae	control	1.00	0.00	1.00
family	Pseudomonadaceae	cephalonium	12463.80	18849.60	22675.60
family	Pseudomonadaceae	cloxacillin	8165.40	12949.00	23133.00
family	Pseudomonadaceae	teat-sealant	14481.00	21316.20	24588.00
family	Pseudomonadaceae	control	14487.60	20431.80	21019.20
family	Pseudonocardiaceae	cephalonium	61.40	125.80	47.80
family	Pseudonocardiaceae	cloxacillin	35.60	0.20	107.80
family	Pseudonocardiaceae	teat-sealant	11.20	515.60	259.00
family	Pseudonocardiaceae	control	30.60	1.60	51.40
family	Rarobacteraceae	cephalonium	0.00	0.60	0.40
family	Rarobacteraceae	cloxacillin	0.20	0.40	0.20
family	Rarobacteraceae	teat-sealant	0.00	0.00	0.80
family	Rarobacteraceae	control	0.00	0.40	0.20
family	RF16	cephalonium	194.60	20.00	58.00
family	RF16	cloxacillin	261.60	24.60	8.40
family	RF16	teat-sealant	89.60	0.20	0.60
family	RF16	control	195.80	27.40	41.80
family	RFP12	cephalonium	15.00	0.20	0.00
family	RFP12	cloxacillin	46.60	0.00	0.00
family	RFP12	teat-sealant	25.60	0.40	0.00
family	RFP12	control	11.60	7.20	0.00
family	Rhizobiaceae	cephalonium	76.00	215.40	139.80
family	Rhizobiaceae	cloxacillin	225.80	73.20	115.80
family	Rhizobiaceae	teat-sealant	114.20	162.40	130.40
family	Rhizobiaceae	control	37.20	86.40	100.60
family	Rhodobacteraceae	cephalonium	212.00	1081.20	198.00
family	Rhodobacteraceae	cloxacillin	514.40	305.60	749.20
family	Rhodobacteraceae	teat-sealant	81.80	446.80	820.80
family	Rhodobacteraceae	control	316.20	196.80	388.80
family	Rhodocyclaceae	cephalonium	0.00	102.20	77.40
family	Rhodocyclaceae	cloxacillin	4.80	0.20	20.40
family	Rhodocyclaceae	teat-sealant	0.40	18.60	13.40
family	Rhodocyclaceae	control	7.60	1.00	0.00
family	Rhodospirillaceae	cephalonium	3.60	463.20	604.00
family	Rhodospirillaceae	cloxacillin	31.00	163.80	990.60
family	Rhodospirillaceae	teat-sealant	46.60	316.20	190.60
family	Rhodospirillaceae	control	44.20	127.40	586.20
family	Rikenellaceae	cephalonium	372.20	27.80	201.80

family	Rikenellaceae	cloxacillin	501.20	41.60	116.80
family	Rikenellaceae	teat-sealant	267.00	0.60	0.80
family	Rikenellaceae	control	259.40	177.00	21.60
family	Ruaniaceae	cephalonium	1.20	10.00	0.00
family	Ruaniaceae	cloxacillin	4.80	7.00	7.00
family	Ruaniaceae	teat-sealant	2.20	0.40	12.80
family	Ruaniaceae	control	7.20	2.00	0.00
family	Ruminococcaceae	cephalonium	4050.60	1202.20	1336.80
family	Ruminococcaceae	cloxacillin	8050.00	396.00	505.80
family	Ruminococcaceae	teat-sealant	2613.00	318.60	597.00
family	Ruminococcaceae	control	3767.60	1610.40	494.20
family	S24-7	cephalonium	121.60	82.20	0.40
family	S24-7	cloxacillin	412.40	13.80	14.00
family	S24-7	teat-sealant	69.00	0.00	0.00
family	S24-7	control	156.80	64.80	0.80
family	Sanguibacteraceae	cephalonium	12.80	41.20	13.00
family	Sanguibacteraceae	cloxacillin	36.60	11.00	16.40
family	Sanguibacteraceae	teat-sealant	13.20	20.20	65.80
family	Sanguibacteraceae	control	23.40	3.80	46.40
family	Saprospiraceae	cephalonium	0.00	0.20	21.40
family	Saprospiraceae	cloxacillin	0.40	0.00	4.80
family	Saprospiraceae	teat-sealant	0.00	0.00	0.00
family	Saprospiraceae	control	0.00	0.00	0.00
family	Shewanellaceae	cephalonium	0.40	0.40	0.40
family	Shewanellaceae	cloxacillin	0.40	7.20	2.40
family	Shewanellaceae	teat-sealant	0.60	13.20	0.60
family	Shewanellaceae	control	0.40	145.60	1.00
family	Sinobacteraceae	cephalonium	0.00	0.00	0.00
family	Sinobacteraceae	cloxacillin	0.40	0.00	0.00
family	Sinobacteraceae	teat-sealant	0.20	104.40	4.00
family	Sinobacteraceae	control	40.40	0.80	1.40
family	Solibacteraceae	cephalonium	0.00	0.00	0.20
family	Solibacteraceae	cloxacillin	0.20	0.00	0.20
family	Solibacteraceae	teat-sealant	0.00	0.00	2.20
family	Solibacteraceae	control	0.00	0.00	0.40
family	Solirubrobacteraceae	cephalonium	0.00	0.00	0.60
family	Solirubrobacteraceae	cloxacillin	0.00	0.80	0.00
family	Solirubrobacteraceae	teat-sealant	0.40	0.20	0.00
family	Solirubrobacteraceae	control	0.00	0.00	11.60
family	Sphingobacteriaceae	cephalonium	421.60	2569.40	554.80
family	Sphingobacteriaceae	cloxacillin	689.00	949.20	1113.40
family	Sphingobacteriaceae	teat-sealant	318.00	21.60	712.40
family	Sphingobacteriaceae	control	164.60	564.40	103.60
family	Sphingomonadaceae	cephalonium	303.00	843.00	180.40
family	Sphingomonadaceae	cloxacillin	568.40	515.00	495.40

family	Sphingomonadaceae	teat-sealant	168.00	405.20	342.80
family	Sphingomonadaceae	control	158.00	305.80	382.80
family	Spirochaetaceae	cephalonium	185.60	1.20	543.80
family	Spirochaetaceae	cloxacillin	81.20	0.00	74.40
family	Spirochaetaceae	teat-sealant	166.20	0.20	0.20
family	Spirochaetaceae	control	133.80	0.40	0.00
family	Sporichthyaceae	cephalonium	0.00	0.00	0.00
family	Sporichthyaceae	cloxacillin	0.00	0.00	1.40
family	Sporichthyaceae	teat-sealant	0.00	0.00	0.00
family	Sporichthyaceae	control	9.20	0.20	0.00
family	Staphylococcaceae	cephalonium	1816.80	1571.20	1783.80
family	Staphylococcaceae	cloxacillin	2211.80	1032.20	2293.00
family	Staphylococcaceae	teat-sealant	1967.00	614.40	2838.40
family	Staphylococcaceae	control	1660.40	1258.20	2820.20
family	Streptococcaceae	cephalonium	7071.80	14625.60	18825.40
family	Streptococcaceae	cloxacillin	5994.40	16302.20	20098.00
family	Streptococcaceae	teat-sealant	10113.80	13143.80	18716.20
family	Streptococcaceae	control	11244.40	15280.00	15659.80
family	Streptomycetaceae	cephalonium	176.40	585.40	167.80
family	Streptomycetaceae	cloxacillin	346.80	168.00	220.80
family	Streptomycetaceae	teat-sealant	146.20	212.20	498.80
family	Streptomycetaceae	control	163.80	46.00	164.20
family	Succinivibrionaceae	cephalonium	166.20	0.00	72.20
family	Succinivibrionaceae	cloxacillin	493.20	27.40	20.60
family	Succinivibrionaceae	teat-sealant	150.60	0.20	20.40
family	Succinivibrionaceae	control	482.00	0.60	0.40
family	Thermaceae	cephalonium	0.00	0.00	0.00
family	Thermaceae	cloxacillin	9.60	0.20	258.40
family	Thermaceae	teat-sealant	61.00	4.40	0.00
family	Thermaceae	control	0.00	0.00	0.00
family	Thermoactinomycetaceae	cephalonium	0.00	1.60	4.20
family	Thermoactinomycetaceae	cloxacillin	2.60	0.00	0.00
family	Thermoactinomycetaceae	teat-sealant	10.60	0.00	76.40
family	Thermoactinomycetaceae	control	4.00	0.00	0.00
family	Thermotogaceae	cephalonium	25.60	131.40	0.00
family	Thermotogaceae	cloxacillin	0.20	464.80	0.60
family	Thermotogaceae	teat-sealant	0.20	0.20	108.80
family	Thermotogaceae	control	0.40	202.80	0.40
family	[Tissierellaceae]	cephalonium	80.00	335.40	100.40
family	[Tissierellaceae]	cloxacillin	182.00	60.20	138.60
family	[Tissierellaceae]	teat-sealant	173.80	49.80	459.80
family	[Tissierellaceae]	control	89.00	0.60	47.00
family	Trueperaceae	cephalonium	9.60	129.80	0.00
family	Trueperaceae	cloxacillin	43.80	2.80	26.60
family	Trueperaceae	teat-sealant	2.60	0.00	0.00

family	Trueperaceae	control	0.00	22.80	0.00
family	Turicibacteraceae	cephalonium	329.20	57.60	2.80
family	Turicibacteraceae	cloxacillin	706.60	4.40	15.40
family	Turicibacteraceae	teat-sealant	216.00	30.20	2.00
family	Turicibacteraceae	control	1963.40	147.20	1.60
family	Veillonellaceae	cephalonium	702.80	428.40	411.00
family	Veillonellaceae	cloxacillin	1922.00	355.40	408.60
family	Veillonellaceae	teat-sealant	758.60	109.20	171.80
family	Veillonellaceae	control	1230.60	1171.00	535.60
family	Verrucomicrobiaceae	cephalonium	99.20	334.80	866.40
family	Verrucomicrobiaceae	cloxacillin	434.00	193.40	268.80
family	Verrucomicrobiaceae	teat-sealant	235.80	777.60	152.40
family	Verrucomicrobiaceae	control	244.80	142.20	384.40
family	Vibrionaceae	cephalonium	4.60	0.00	0.00
family	Vibrionaceae	cloxacillin	1.60	0.00	0.00
family	Vibrionaceae	teat-sealant	0.00	0.00	0.00
family	Vibrionaceae	control	0.00	0.00	0.00
family	Victivallaceae	cephalonium	1.20	0.00	0.00
family	Victivallaceae	cloxacillin	10.00	0.00	0.00
family	Victivallaceae	teat-sealant	8.40	0.00	0.00
family	Victivallaceae	control	0.00	0.00	0.00
family	[Weeksellaceae]	cephalonium	731.60	3063.60	1119.40
family	[Weeksellaceae]	cloxacillin	1033.20	693.40	2052.00
family	[Weeksellaceae]	teat-sealant	422.40	168.20	761.20
family	[Weeksellaceae]	control	468.40	442.80	421.60
family	Williamsiaceae	cephalonium	37.00	0.00	0.20
family	Williamsiaceae	cloxacillin	0.60	0.00	0.00
family	Williamsiaceae	teat-sealant	0.20	0.00	0.60
family	Williamsiaceae	control	0.00	0.00	0.00
family	Xanthobacteraceae	cephalonium	0.00	0.40	0.00
family	Xanthobacteraceae	cloxacillin	0.00	0.00	0.00
family	Xanthobacteraceae	teat-sealant	0.00	11.60	1.60
family	Xanthobacteraceae	control	0.40	14.40	0.20
family	Xanthomonadaceae	cephalonium	367.60	2341.40	358.20
family	Xanthomonadaceae	cloxacillin	963.40	1185.80	1327.40
family	Xanthomonadaceae	teat-sealant	622.00	557.40	573.60
family	Xanthomonadaceae	control	403.20	756.40	357.00
family	Xenococcaceae	cephalonium	0.00	83.60	30.00
family	Xenococcaceae	cloxacillin	0.00	0.00	0.00
family	Xenococcaceae	teat-sealant	0.00	0.00	0.00
family	Xenococcaceae	control	0.00	0.00	0.00
family	Yaniellaceae	cephalonium	73.80	248.40	66.00
family	Yaniellaceae	cloxacillin	280.20	153.00	145.40
family	Yaniellaceae	teat-sealant	32.40	0.40	42.00
family	Yaniellaceae	control	52.40	10.20	3.20

genus	5-7N15	cephalonium	1250.40	336.00	282.80
genus	5-7N15	cloxacillin	1522.20	56.00	64.80
genus	5-7N15	teat-sealant	581.20	0.80	359.20
genus	5-7N15	control	985.60	508.00	173.80
genus	Abiotrophia	cephalonium	0.00	0.00	0.00
genus	Abiotrophia	cloxacillin	0.00	0.00	0.00
genus	Abiotrophia	teat-sealant	4.00	0.20	0.00
genus	Abiotrophia	control	0.00	0.00	0.00
genus	Acholeplasma	cephalonium	12.20	0.00	6.20
genus	Acholeplasma	cloxacillin	40.40	0.40	17.00
genus	Acholeplasma	teat-sealant	6.60	0.00	0.00
genus	Acholeplasma	control	9.40	0.20	0.00
genus	Achromobacter	cephalonium	100.40	131.60	196.80
genus	Achromobacter	cloxacillin	72.80	147.80	185.80
genus	Achromobacter	teat-sealant	139.60	219.80	218.80
genus	Achromobacter	control	119.40	139.20	188.20
genus	Acidovorax	cephalonium	9.20	5.00	2.60
genus	Acidovorax	cloxacillin	17.00	1.40	18.00
genus	Acidovorax	teat-sealant	1.20	0.20	4.80
genus	Acidovorax	control	2.20	0.00	1.80
genus	Acinetobacter	cephalonium	1412.20	3053.40	2595.00
genus	Acinetobacter	cloxacillin	2150.80	1295.00	6365.00
genus	Acinetobacter	teat-sealant	937.60	705.80	1980.60
genus	Acinetobacter	control	1375.60	1167.60	670.60
genus	Actinobacillus	cephalonium	0.00	0.00	0.00
genus	Actinobacillus	cloxacillin	0.00	0.20	0.00
genus	Actinobacillus	teat-sealant	0.40	0.00	0.00
genus	Actinobacillus	control	0.00	0.00	1.60
genus	Actinokineospora	cephalonium	0.80	2.60	0.20
genus	Actinokineospora	cloxacillin	0.00	0.20	1.80
genus	Actinokineospora	teat-sealant	0.20	1.00	1.20
genus	Actinokineospora	control	0.80	0.80	0.00
genus	Actinomyces	cephalonium	15.00	71.00	46.40
genus	Actinomyces	cloxacillin	2.00	1.40	135.80
genus	Actinomyces	teat-sealant	35.00	18.80	14.40
genus	Actinomyces	control	172.80	100.60	35.00
genus	Actinomycetospora	cephalonium	18.80	3.40	0.00
genus	Actinomycetospora	cloxacillin	0.00	0.00	0.00
genus	Actinomycetospora	teat-sealant	0.00	0.00	0.80
genus	Actinomycetospora	control	0.00	0.40	0.00
genus	Actinoplanes	cephalonium	0.00	22.60	0.00
genus	Actinoplanes	cloxacillin	0.80	3.20	0.00
genus	Actinoplanes	teat-sealant	0.20	9.60	0.00
genus	Actinoplanes	control	4.60	0.40	0.40
genus	Actinotalea	cephalonium	0.00	5.00	0.00

genus	Actinotalea	cloxacillin	0.00	1.00	1.20
genus	Actinotalea	teat-sealant	0.00	0.00	0.00
genus	Actinotalea	control	0.00	6.80	0.00
genus	Aequorivita	cephalonium	0.00	1.00	0.00
genus	Aequorivita	cloxacillin	0.00	3.00	0.00
genus	Aequorivita	teat-sealant	0.00	0.00	0.00
genus	Aequorivita	control	0.00	0.00	0.00
genus	Aerococcus	cephalonium	149.80	358.00	345.00
genus	Aerococcus	cloxacillin	390.00	156.80	788.80
genus	Aerococcus	teat-sealant	259.00	27.40	241.80
genus	Aerococcus	control	47.40	53.60	23.80
genus	Aeromicrobium	cephalonium	26.60	63.40	2.20
genus	Aeromicrobium	cloxacillin	13.00	1.00	5.00
genus	Aeromicrobium	teat-sealant	0.60	0.20	28.00
genus	Aeromicrobium	control	29.60	5.20	0.00
genus	Afipia	cephalonium	0.00	0.40	0.80
genus	Afipia	cloxacillin	0.00	0.00	0.00
genus	Afipia	teat-sealant	0.20	0.40	0.20
genus	Afipia	control	0.00	0.00	0.00
genus	Aggregatibacter	cephalonium	22.80	0.00	0.00
genus	Aggregatibacter	cloxacillin	5.20	0.00	24.20
genus	Aggregatibacter	teat-sealant	3.20	47.40	0.00
genus	Aggregatibacter	control	122.20	0.00	0.40
genus	Agrobacterium	cephalonium	48.80	128.20	92.80
genus	Agrobacterium	cloxacillin	144.60	28.40	88.80
genus	Agrobacterium	teat-sealant	61.80	119.40	85.00
genus	Agrobacterium	control	26.80	59.00	49.40
genus	Agrococcus	cephalonium	1.40	97.60	0.20
genus	Agrococcus	cloxacillin	1.40	16.20	22.60
genus	Agrococcus	teat-sealant	9.80	0.20	0.00
genus	Agrococcus	control	3.60	15.20	4.40
genus	Agromyces	cephalonium	0.00	0.00	1.20
genus	Agromyces	cloxacillin	0.00	14.60	1.60
genus	Agromyces	teat-sealant	0.80	3.80	0.00
genus	Agromyces	control	0.00	0.00	0.00
genus	Akkermansia	cephalonium	98.80	70.20	0.00
genus	Akkermansia	cloxacillin	433.80	65.00	128.60
genus	Akkermansia	teat-sealant	235.00	0.00	0.20
genus	Akkermansia	control	244.60	90.40	2.40
genus	Alcaligenes	cephalonium	22.40	66.40	54.00
genus	Alcaligenes	cloxacillin	11.80	40.00	47.40
genus	Alcaligenes	teat-sealant	19.80	1.20	47.20
genus	Alcaligenes	control	14.40	3.40	10.20
genus	Alcanivorax	cephalonium	32.40	129.40	89.80
genus	Alcanivorax	cloxacillin	24.80	9.00	115.00

genus	Alcanivorax	teat-sealant	47.80	133.20	64.60
genus	Alcanivorax	control	102.40	55.00	104.20
genus	Alkalibacterium	cephalonium	68.80	59.60	74.60
genus	Alkalibacterium	cloxacillin	71.80	20.60	3.00
genus	Alkalibacterium	teat-sealant	34.40	4.00	21.60
genus	Alkalibacterium	control	54.60	33.00	11.40
genus	Alkaliphilus	cephalonium	0.00	0.00	0.00
genus	Alkaliphilus	cloxacillin	1.00	0.00	0.00
genus	Alkaliphilus	teat-sealant	0.20	0.00	0.00
genus	Alkaliphilus	control	1.00	0.00	1.00
genus	Alkanindiges	cephalonium	0.80	3.60	1.40
genus	Alkanindiges	cloxacillin	0.20	1.40	0.00
genus	Alkanindiges	teat-sealant	0.40	0.00	0.40
genus	Alkanindiges	control	2.20	1.00	1.00
genus	Alloiococcus	cephalonium	99.80	13.60	4.00
genus	Alloiococcus	cloxacillin	135.80	18.00	19.20
genus	Alloiococcus	teat-sealant	23.40	5.40	4.80
genus	Alloiococcus	control	56.00	3.40	0.80
genus	Amaricoccus	cephalonium	0.00	0.60	0.00
genus	Amaricoccus	cloxacillin	0.00	0.00	3.40
genus	Amaricoccus	teat-sealant	0.00	0.00	57.00
genus	Amaricoccus	control	0.20	0.00	0.00
genus	Aminobacter	cephalonium	4.00	37.40	73.20
genus	Aminobacter	cloxacillin	28.00	7.80	30.20
genus	Aminobacter	teat-sealant	2.20	7.40	29.20
genus	Aminobacter	control	5.60	1.80	5.20
genus	Anaerococcus	cephalonium	17.00	335.20	93.20
genus	Anaerococcus	cloxacillin	8.00	55.80	44.40
genus	Anaerococcus	teat-sealant	84.60	1.00	15.00
genus	Anaerococcus	control	1.80	0.20	45.60
genus	Anaerofustis	cephalonium	0.00	18.80	0.00
genus	Anaerofustis	cloxacillin	0.00	0.00	0.00
genus	Anaerofustis	teat-sealant	0.00	0.00	0.00
genus	Anaerofustis	control	78.80	8.40	0.20
genus	Anaeroplasma	cephalonium	0.00	0.00	0.00
genus	Anaeroplasma	cloxacillin	0.40	0.00	0.00
genus	Anaeroplasma	teat-sealant	0.00	0.00	0.00
genus	Anaeroplasma	control	3.00	0.00	0.00
genus	Anaerospora	cephalonium	44.00	295.80	1.60
genus	Anaerospora	cloxacillin	126.80	72.00	234.60
genus	Anaerospora	teat-sealant	7.80	9.40	338.60
genus	Anaerospora	control	39.20	21.60	75.00
genus	Anaerostipes	cephalonium	100.60	7.60	60.20
genus	Anaerostipes	cloxacillin	181.80	10.80	32.00
genus	Anaerostipes	teat-sealant	29.40	0.60	14.80

genus	Anaerostipes	control	57.60	0.40	0.00
genus	Anaerovibrio	cephalonium	16.00	0.00	7.80
genus	Anaerovibrio	cloxacillin	64.20	0.20	100.00
genus	Anaerovibrio	teat-sealant	0.00	0.00	0.00
genus	Anaerovibrio	control	32.00	0.20	0.20
genus	Ancylobacter	cephalonium	0.00	0.00	0.00
genus	Ancylobacter	cloxacillin	0.00	0.00	0.00
genus	Ancylobacter	teat-sealant	0.00	4.20	0.00
genus	Ancylobacter	control	0.00	0.00	0.00
genus	Arcanobacterium	cephalonium	7.20	0.00	4.20
genus	Arcanobacterium	cloxacillin	0.80	0.00	0.00
genus	Arcanobacterium	teat-sealant	1.20	0.00	6.20
genus	Arcanobacterium	control	0.00	0.00	0.00
genus	Arcobacter	cephalonium	46.60	0.00	0.20
genus	Arcobacter	cloxacillin	41.80	15.00	169.40
genus	Arcobacter	teat-sealant	68.60	3.80	0.00
genus	Arcobacter	control	32.80	0.40	0.00
genus	Arthrobacter	cephalonium	452.60	2107.60	757.80
genus	Arthrobacter	cloxacillin	436.40	526.60	847.20
genus	Arthrobacter	teat-sealant	351.80	380.80	849.40
genus	Arthrobacter	control	176.60	163.20	135.00
genus	Arthrospira	cephalonium	6.00	42.60	0.00
genus	Arthrospira	cloxacillin	6.40	29.60	0.00
genus	Arthrospira	teat-sealant	0.00	35.00	0.00
genus	Arthrospira	control	0.00	23.20	0.00
genus	Atopobium	cephalonium	3.80	3.60	0.00
genus	Atopobium	cloxacillin	28.40	0.20	3.20
genus	Atopobium	teat-sealant	0.00	0.00	9.80
genus	Atopobium	control	2.40	0.80	1.00
genus	Aurantimonas	cephalonium	0.00	0.00	0.00
genus	Aurantimonas	cloxacillin	1.80	0.00	0.00
genus	Aurantimonas	teat-sealant	0.00	0.00	0.00
genus	Aurantimonas	control	1.40	0.80	1.00
genus	Azospirillum	cephalonium	0.00	0.00	0.00
genus	Azospirillum	cloxacillin	0.00	0.00	21.00
genus	Azospirillum	teat-sealant	0.00	5.40	57.20
genus	Azospirillum	control	0.00	42.80	0.00
genus	B-42	cephalonium	9.60	129.80	0.00
genus	B-42	cloxacillin	43.80	2.80	26.60
genus	B-42	teat-sealant	2.60	0.00	0.00
genus	B-42	control	0.00	22.80	0.00
genus	Bacillus	cephalonium	55.20	148.40	30.60
genus	Bacillus	cloxacillin	70.80	72.60	80.60
genus	Bacillus	teat-sealant	28.00	6.20	95.60
genus	Bacillus	control	61.00	42.00	9.20

genus	Bacteroides	cephalonium	96.40	352.20	30.40
genus	Bacteroides	cloxacillin	87.80	4.00	3.60
genus	Bacteroides	teat-sealant	69.60	0.40	6.00
genus	Bacteroides	control	35.00	0.00	6.00
genus	Balneimonas	cephalonium	0.00	0.00	0.00
genus	Balneimonas	cloxacillin	0.00	0.60	0.00
genus	Balneimonas	teat-sealant	0.00	0.00	1.20
genus	Balneimonas	control	0.00	1.00	1.80
genus	Bartonella	cephalonium	0.00	0.00	0.00
genus	Bartonella	cloxacillin	0.00	0.00	0.00
genus	Bartonella	teat-sealant	0.00	145.80	0.20
genus	Bartonella	control	0.00	0.00	0.00
genus	BD2-13	cephalonium	9.00	111.20	5.20
genus	BD2-13	cloxacillin	23.00	27.80	172.40
genus	BD2-13	teat-sealant	3.40	0.20	0.00
genus	BD2-13	control	0.00	4.80	0.00
genus	Beijerinckia	cephalonium	1.60	0.00	1.00
genus	Beijerinckia	cloxacillin	0.40	0.00	4.60
genus	Beijerinckia	teat-sealant	36.00	17.20	2.00
genus	Beijerinckia	control	0.60	3.00	19.40
genus	Bibersteinia	cephalonium	0.00	0.00	78.20
genus	Bibersteinia	cloxacillin	0.00	0.00	0.00
genus	Bibersteinia	teat-sealant	0.00	0.00	0.20
genus	Bibersteinia	control	0.00	0.00	0.00
genus	Bifidobacterium	cephalonium	144.40	122.40	170.40
genus	Bifidobacterium	cloxacillin	704.40	122.40	68.60
genus	Bifidobacterium	teat-sealant	86.40	152.60	21.60
genus	Bifidobacterium	control	268.20	1136.60	0.20
genus	Blastobacter	cephalonium	8.80	57.40	0.60
genus	Blastobacter	cloxacillin	1.00	2.40	0.20
genus	Blastobacter	teat-sealant	9.00	0.40	37.00
genus	Blastobacter	control	5.20	5.40	23.80
genus	Blastococcus	cephalonium	0.00	0.00	0.00
genus	Blastococcus	cloxacillin	0.20	4.40	21.60
genus	Blastococcus	teat-sealant	0.00	0.00	0.00
genus	Blastococcus	control	0.00	0.00	0.00
genus	Blastomonas	cephalonium	0.00	0.40	0.00
genus	Blastomonas	cloxacillin	0.00	0.60	0.20
genus	Blastomonas	teat-sealant	0.00	0.00	0.20
genus	Blastomonas	control	0.00	0.40	0.20
genus	Blautia	cephalonium	25.40	0.60	3.00
genus	Blautia	cloxacillin	29.60	0.20	8.00
genus	Blautia	teat-sealant	3.00	0.60	18.40
genus	Blautia	control	4.00	4.80	3.40
genus	Bosea	cephalonium	0.60	1.20	1.20

genus	Bosea	cloxacillin	0.20	0.00	59.20
genus	Bosea	teat-sealant	0.00	0.60	0.40
genus	Bosea	control	0.60	0.40	0.40
genus	Brachybacterium	cephalonium	35.60	99.40	95.60
genus	Brachybacterium	cloxacillin	95.60	20.00	305.60
genus	Brachybacterium	teat-sealant	214.80	8.00	754.20
genus	Brachybacterium	control	28.20	28.40	4.00
genus	Bradyrhizobium	cephalonium	421.20	825.80	848.00
genus	Bradyrhizobium	cloxacillin	390.40	202.40	688.80
genus	Bradyrhizobium	teat-sealant	440.00	1401.40	588.20
genus	Bradyrhizobium	control	686.20	332.40	741.40
genus	Brevibacterium	cephalonium	95.80	619.00	416.60
genus	Brevibacterium	cloxacillin	412.60	230.60	849.00
genus	Brevibacterium	teat-sealant	415.00	42.80	1552.60
genus	Brevibacterium	control	84.00	150.20	113.00
genus	Brevundimonas	cephalonium	71.00	315.60	0.40
genus	Brevundimonas	cloxacillin	78.40	28.80	36.00
genus	Brevundimonas	teat-sealant	26.80	0.00	7.20
genus	Brevundimonas	control	16.20	20.60	0.80
genus	Brochothrix	cephalonium	0.20	0.00	0.00
genus	Brochothrix	cloxacillin	0.00	0.00	0.20
genus	Brochothrix	teat-sealant	90.40	0.00	0.00
genus	Brochothrix	control	0.00	0.20	120.60
genus	Brumimicrobium	cephalonium	8.00	59.40	0.20
genus	Brumimicrobium	cloxacillin	14.40	19.40	6.00
genus	Brumimicrobium	teat-sealant	1.60	0.00	0.00
genus	Brumimicrobium	control	0.00	3.60	0.00
genus	Bulleidia	cephalonium	3.20	0.20	0.00
genus	Bulleidia	cloxacillin	4.00	0.00	0.00
genus	Bulleidia	teat-sealant	2.40	0.00	0.00
genus	Bulleidia	control	0.20	0.00	251.80
genus	Butyrivibrio	cephalonium	377.00	182.80	251.40
genus	Butyrivibrio	cloxacillin	932.40	73.40	275.00
genus	Butyrivibrio	teat-sealant	277.40	93.40	24.40
genus	Butyrivibrio	control	207.20	84.00	91.40
genus	C39	cephalonium	0.00	89.00	0.00
genus	C39	cloxacillin	0.00	0.20	0.00
genus	C39	teat-sealant	0.00	0.00	0.00
genus	C39	control	0.00	0.00	0.00
genus	Caldicoprobacter	cephalonium	0.00	0.20	7.00
genus	Caldicoprobacter	cloxacillin	0.00	0.00	0.00
genus	Caldicoprobacter	teat-sealant	0.00	0.00	0.00
genus	Caldicoprobacter	control	0.00	0.00	0.00
genus	Caldilinea	cephalonium	0.20	189.60	0.00
genus	Caldilinea	cloxacillin	0.00	0.00	258.20

genus	Caldilinea	teat-sealant	0.20	0.20	156.80
genus	Caldilinea	control	0.00	0.20	0.00
genus	Campylobacter	cephalonium	17.60	0.00	0.00
genus	Campylobacter	cloxacillin	65.20	10.80	0.00
genus	Campylobacter	teat-sealant	11.80	0.00	0.00
genus	Campylobacter	control	24.80	49.60	0.20
genus	Candidatus Aquiluna	cephalonium	0.20	6.20	0.20
genus	Candidatus Aquiluna	cloxacillin	2.00	2.40	1.00
genus	Candidatus Aquiluna	teat-sealant	0.80	0.40	0.40
genus	Candidatus Aquiluna	control	1.40	1.20	10.20
genus	Candidatus Portiera	cephalonium	0.00	0.00	0.00
genus	Candidatus Portiera	cloxacillin	25.20	41.20	19.20
genus	Candidatus Portiera	teat-sealant	0.00	0.00	0.00
genus	Candidatus Portiera	control	0.00	0.00	0.00
genus	Candidatus Rhodoluna	cephalonium	6.00	107.80	2.00
genus	Candidatus Rhodoluna	cloxacillin	22.40	19.00	5.80
genus	Candidatus Rhodoluna	teat-sealant	7.60	1.40	10.40
genus	Candidatus Rhodoluna	control	13.40	4.20	283.60
genus	Candidatus Solibacter	cephalonium	0.00	0.00	0.20
genus	Candidatus Solibacter	cloxacillin	0.20	0.00	0.20
genus	Candidatus Solibacter	teat-sealant	0.00	0.00	2.20
genus	Candidatus Solibacter	control	0.00	0.00	0.40
genus	Cardiobacterium	cephalonium	18.40	0.00	0.00
genus	Cardiobacterium	cloxacillin	0.40	0.20	198.00
genus	Cardiobacterium	teat-sealant	0.00	0.00	0.00
genus	Cardiobacterium	control	0.00	0.00	0.00
genus	Carica	cephalonium	0.00	0.40	1.20
genus	Carica	cloxacillin	0.00	0.00	1.40
genus	Carica	teat-sealant	0.00	0.00	1.00
genus	Carica	control	0.00	0.20	3.40
genus	Carnobacterium	cephalonium	0.60	0.40	0.00
genus	Carnobacterium	cloxacillin	0.20	5.00	0.60
genus	Carnobacterium	teat-sealant	0.80	0.60	0.00
genus	Carnobacterium	control	0.40	31.00	5.00
genus	Caulobacter	cephalonium	3.60	18.40	7.20
genus	Caulobacter	cloxacillin	4.00	5.80	0.40
genus	Caulobacter	teat-sealant	4.20	4.40	13.40
genus	Caulobacter	control	2.00	2.20	9.20
genus	Cellulomonas	cephalonium	4.20	2.00	0.80
genus	Cellulomonas	cloxacillin	9.20	2.40	1.00
genus	Cellulomonas	teat-sealant	0.80	1.20	21.20
genus	Cellulomonas	control	1.60	1.20	3.20
genus	Cellvibrio	cephalonium	73.40	891.60	52.60
genus	Cellvibrio	cloxacillin	125.40	74.60	90.40
genus	Cellvibrio	teat-sealant	12.80	0.80	81.60

genus	Cellvibrio	control	59.20	12.20	33.60
genus	CF231	cephalonium	122.60	31.60	97.60
genus	CF231	cloxacillin	1076.40	129.00	23.80
genus	CF231	teat-sealant	34.60	0.20	4.40
genus	CF231	control	148.60	0.80	15.40
genus	Chelativorans	cephalonium	0.00	0.00	0.60
genus	Chelativorans	cloxacillin	0.20	0.00	0.40
genus	Chelativorans	teat-sealant	0.00	0.20	0.20
genus	Chelativorans	control	0.40	0.20	0.40
genus	Chroococcidiopsis	cephalonium	0.00	0.20	29.60
genus	Chroococcidiopsis	cloxacillin	0.00	0.00	0.00
genus	Chroococcidiopsis	teat-sealant	0.00	0.00	0.00
genus	Chroococcidiopsis	control	0.00	0.00	0.00
genus	Chryseobacterium	cephalonium	111.20	26.00	115.60
genus	Chryseobacterium	cloxacillin	206.40	48.40	107.60
genus	Chryseobacterium	teat-sealant	61.60	75.00	40.20
genus	Chryseobacterium	control	96.60	1.00	63.20
genus	Chthoniobacter	cephalonium	0.00	0.00	0.00
genus	Chthoniobacter	cloxacillin	0.40	0.00	88.80
genus	Chthoniobacter	teat-sealant	0.00	0.00	0.00
genus	Chthoniobacter	control	0.00	0.00	0.00
genus	Citricoccus	cephalonium	18.60	33.00	15.80
genus	Citricoccus	cloxacillin	51.80	6.60	3.20
genus	Citricoccus	teat-sealant	13.20	0.00	0.80
genus	Citricoccus	control	1.60	0.20	0.00
genus	Cloacibacterium	cephalonium	0.00	0.80	0.00
genus	Cloacibacterium	cloxacillin	0.00	1.00	7.60
genus	Cloacibacterium	teat-sealant	0.00	44.00	3.20
genus	Cloacibacterium	control	2.80	35.00	32.00
genus	Clostridium	cephalonium	115.30	191.10	41.90
genus	Clostridium	cloxacillin	324.30	16.80	18.90
genus	Clostridium	teat-sealant	75.70	8.20	43.20
genus	Clostridium	control	121.30	39.50	60.00
genus	[Clostridium]	cephalonium	107.60	29.00	67.60
genus	[Clostridium]	cloxacillin	263.00	28.00	21.40
genus	[Clostridium]	teat-sealant	82.60	22.40	39.80
genus	[Clostridium]	control	31.60	9.00	19.60
genus	Collinsella	cephalonium	0.00	16.40	0.00
genus	Collinsella	cloxacillin	0.00	0.00	1.40
genus	Collinsella	teat-sealant	10.20	0.20	0.00
genus	Collinsella	control	2.20	0.00	0.00
genus	Comamonas	cephalonium	105.60	968.00	414.40
genus	Comamonas	cloxacillin	624.00	96.40	395.80
genus	Comamonas	teat-sealant	120.60	30.20	53.00
genus	Comamonas	control	194.60	134.60	33.20

genus	Coprobacillus	cephalonium	0.00	0.00	0.00
genus	Coprobacillus	cloxacillin	0.40	0.00	0.00
genus	Coprobacillus	teat-sealant	1.40	0.00	0.00
genus	Coprobacillus	control	2.40	0.00	0.00
genus	Coprococcus	cephalonium	160.20	33.60	78.80
genus	Coprococcus	cloxacillin	287.40	12.60	7.80
genus	Coprococcus	teat-sealant	49.20	20.80	63.00
genus	Coprococcus	control	116.20	46.40	10.80
genus	Corynebacterium	cephalonium	2041.80	2544.00	1946.40
genus	Corynebacterium	cloxacillin	3878.60	1230.40	1432.40
genus	Corynebacterium	teat-sealant	1926.20	1073.00	2556.60
genus	Corynebacterium	control	1651.40	1155.40	850.20
genus	Coxiella	cephalonium	297.80	2.60	46.00
genus	Coxiella	cloxacillin	80.80	245.20	3.20
genus	Coxiella	teat-sealant	260.20	553.80	91.20
genus	Coxiella	control	3.00	642.40	1.00
genus	Cronobacter	cephalonium	0.80	0.40	2.80
genus	Cronobacter	cloxacillin	0.00	0.00	1.20
genus	Cronobacter	teat-sealant	0.00	0.00	4.40
genus	Cronobacter	control	0.00	0.20	73.20
genus	Cryocola	cephalonium	0.80	0.20	0.60
genus	Cryocola	cloxacillin	3.40	0.40	0.60
genus	Cryocola	teat-sealant	1.20	0.80	0.20
genus	Cryocola	control	1.40	0.20	0.20
genus	Cryptosporangium	cephalonium	0.60	4.20	0.20
genus	Cryptosporangium	cloxacillin	0.20	1.40	3.00
genus	Cryptosporangium	teat-sealant	0.00	1.20	1.60
genus	Cryptosporangium	control	0.80	1.00	0.20
genus	Curtobacterium	cephalonium	0.00	0.60	0.00
genus	Curtobacterium	cloxacillin	0.80	0.00	0.40
genus	Curtobacterium	teat-sealant	0.80	3.80	0.00
genus	Curtobacterium	control	0.00	0.20	0.00
genus	DA101	cephalonium	0.00	0.00	0.00
genus	DA101	cloxacillin	20.60	0.00	0.00
genus	DA101	teat-sealant	0.00	0.00	0.00
genus	DA101	control	0.00	107.20	0.00
genus	Dechloromonas	cephalonium	0.00	8.80	0.00
genus	Dechloromonas	cloxacillin	0.00	0.00	0.00
genus	Dechloromonas	teat-sealant	0.00	0.00	4.20
genus	Dechloromonas	control	0.80	0.00	0.00
genus	Deinococcus	cephalonium	62.00	0.00	0.00
genus	Deinococcus	cloxacillin	233.40	0.40	0.20
genus	Deinococcus	teat-sealant	140.40	1.80	0.20
genus	Deinococcus	control	64.00	0.00	53.20
genus	Delftia	cephalonium	0.00	0.00	10.40

genus	Delftia	cloxacillin	0.00	0.80	90.80
genus	Delftia	teat-sealant	5.80	6.40	10.00
genus	Delftia	control	0.00	0.00	0.00
genus	Demequina	cephalonium	63.40	62.00	14.40
genus	Demequina	cloxacillin	29.20	13.60	35.40
genus	Demequina	teat-sealant	13.20	3.40	321.40
genus	Demequina	control	18.20	6.40	0.00
genus	Denitrobacter	cephalonium	0.20	1.00	1.80
genus	Denitrobacter	cloxacillin	0.00	0.00	0.40
genus	Denitrobacter	teat-sealant	0.40	0.60	0.00
genus	Denitrobacter	control	26.80	2.20	20.40
genus	Dermabacter	cephalonium	0.00	1.60	3.40
genus	Dermabacter	cloxacillin	0.00	0.00	3.00
genus	Dermabacter	teat-sealant	0.20	0.00	1.40
genus	Dermabacter	control	0.20	0.20	0.00
genus	Dermacoccus	cephalonium	3.00	34.20	0.00
genus	Dermacoccus	cloxacillin	2.20	2.20	59.80
genus	Dermacoccus	teat-sealant	2.80	0.00	84.80
genus	Dermacoccus	control	0.60	0.00	0.00
genus	Desemzia	cephalonium	8.80	2.60	0.00
genus	Desemzia	cloxacillin	8.40	0.00	11.20
genus	Desemzia	teat-sealant	36.00	0.00	0.00
genus	Desemzia	control	6.40	0.00	93.00
genus	Devosia	cephalonium	34.40	183.40	60.60
genus	Devosia	cloxacillin	222.20	153.00	246.60
genus	Devosia	teat-sealant	66.00	73.40	75.00
genus	Devosia	control	11.60	381.60	5.20
genus	Diaphorobacter	cephalonium	0.40	0.00	2.80
genus	Diaphorobacter	cloxacillin	0.60	0.00	0.00
genus	Diaphorobacter	teat-sealant	0.00	0.00	0.00
genus	Diaphorobacter	control	0.20	0.20	12.00
genus	Dietzia	cephalonium	79.40	84.00	170.80
genus	Dietzia	cloxacillin	258.20	34.40	91.80
genus	Dietzia	teat-sealant	60.60	58.00	55.40
genus	Dietzia	control	84.40	2.00	55.20
genus	Dorea	cephalonium	225.60	55.00	13.40
genus	Dorea	cloxacillin	560.80	6.20	11.80
genus	Dorea	teat-sealant	127.80	0.40	53.60
genus	Dorea	control	157.00	44.00	22.40
genus	Dyadobacter	cephalonium	0.00	5.20	0.00
genus	Dyadobacter	cloxacillin	1.00	14.40	32.20
genus	Dyadobacter	teat-sealant	11.60	0.00	0.00
genus	Dyadobacter	control	22.00	3.60	14.00
genus	Echinicola	cephalonium	0.20	8.40	0.00
genus	Echinicola	cloxacillin	1.60	2.60	0.00

genus	Echinicola	teat-sealant	0.00	0.00	0.40
genus	Echinicola	control	0.00	0.00	0.00
genus	Elizabethkingia	cephalonium	0.60	0.00	0.00
genus	Elizabethkingia	cloxacillin	0.40	0.00	0.00
genus	Elizabethkingia	teat-sealant	0.00	0.00	0.00
genus	Elizabethkingia	control	4.80	0.00	0.00
genus	Elstera	cephalonium	0.00	93.60	0.00
genus	Elstera	cloxacillin	0.00	0.00	0.20
genus	Elstera	teat-sealant	0.00	0.00	0.00
genus	Elstera	control	0.00	0.00	0.00
genus	Emticicia	cephalonium	0.00	329.20	138.80
genus	Emticicia	cloxacillin	0.40	0.20	536.80
genus	Emticicia	teat-sealant	0.20	0.00	68.00
genus	Emticicia	control	0.00	0.00	0.00
genus	Enhydrobacter	cephalonium	453.60	847.40	2555.20
genus	Enhydrobacter	cloxacillin	534.40	842.20	2449.00
genus	Enhydrobacter	teat-sealant	533.80	926.00	2532.80
genus	Enhydrobacter	control	1061.80	1054.80	3851.20
genus	Enterobacter	cephalonium	0.00	20.00	0.00
genus	Enterobacter	cloxacillin	1.00	0.80	6.60
genus	Enterobacter	teat-sealant	0.00	0.00	1.40
genus	Enterobacter	control	1.20	3.60	105.00
genus	Enterococcus	cephalonium	9.80	145.40	11.40
genus	Enterococcus	cloxacillin	5.00	0.40	39.40
genus	Enterococcus	teat-sealant	0.00	1.60	132.20
genus	Enterococcus	control	3.00	229.80	0.60
genus	Epulopiscium	cephalonium	149.00	19.80	0.80
genus	Epulopiscium	cloxacillin	108.60	6.00	3.20
genus	Epulopiscium	teat-sealant	68.60	12.80	0.00
genus	Epulopiscium	control	38.00	46.40	0.00
genus	Erwinia	cephalonium	9.00	12.20	0.40
genus	Erwinia	cloxacillin	3.60	9.00	10.60
genus	Erwinia	teat-sealant	1.20	0.20	42.20
genus	Erwinia	control	1.60	10.00	0.00
genus	Erysipelothrix	cephalonium	0.20	11.60	153.00
genus	Erysipelothrix	cloxacillin	0.00	3.20	11.20
genus	Erysipelothrix	teat-sealant	0.00	0.00	0.00
genus	Erysipelothrix	control	0.80	0.00	0.40
genus	Erythrobacter	cephalonium	7.80	74.20	1.40
genus	Erythrobacter	cloxacillin	11.60	23.60	4.20
genus	Erythrobacter	teat-sealant	1.40	3.60	2.00
genus	Erythrobacter	control	2.80	10.20	3.00
genus	Erythromicrobium	cephalonium	4.00	0.00	0.00
genus	Erythromicrobium	cloxacillin	0.20	0.40	6.40
genus	Erythromicrobium	teat-sealant	0.40	0.20	1.60

genus	Erythromicrobium	control	0.60	0.00	0.00
genus	[Eubacterium]	cephalonium	18.00	0.40	0.60
genus	[Eubacterium]	cloxacillin	40.60	0.00	0.80
genus	[Eubacterium]	teat-sealant	6.40	0.00	0.00
genus	[Eubacterium]	control	0.60	0.00	11.00
genus	Exiguobacterium	cephalonium	7.60	0.00	0.00
genus	Exiguobacterium	cloxacillin	2.20	0.20	41.60
genus	Exiguobacterium	teat-sealant	0.00	0.40	0.00
genus	Exiguobacterium	control	28.60	0.20	0.20
genus	Facklamia	cephalonium	329.00	485.00	370.00
genus	Facklamia	cloxacillin	730.60	180.80	104.60
genus	Facklamia	teat-sealant	231.80	163.20	445.20
genus	Facklamia	control	138.00	147.40	169.80
genus	Faecalibacterium	cephalonium	0.00	0.20	0.00
genus	Faecalibacterium	cloxacillin	1.20	0.00	0.00
genus	Faecalibacterium	teat-sealant	0.00	0.60	0.00
genus	Faecalibacterium	control	1.20	0.40	0.00
genus	Fimbriimonas	cephalonium	0.00	0.00	0.00
genus	Fimbriimonas	cloxacillin	0.00	0.00	0.00
genus	Fimbriimonas	teat-sealant	0.00	0.40	76.80
genus	Fimbriimonas	control	0.00	0.00	0.00
genus	Finegoldia	cephalonium	4.80	0.00	0.20
genus	Finegoldia	cloxacillin	0.00	0.00	0.60
genus	Finegoldia	teat-sealant	0.00	0.00	7.00
genus	Finegoldia	control	14.20	0.20	0.00
genus	Flavisolibacter	cephalonium	0.00	0.00	0.00
genus	Flavisolibacter	cloxacillin	1.80	0.00	21.20
genus	Flavisolibacter	teat-sealant	0.00	0.40	0.00
genus	Flavisolibacter	control	124.60	0.20	2.00
genus	Flavobacterium	cephalonium	0.40	801.60	57.20
genus	Flavobacterium	cloxacillin	1.20	32.00	266.40
genus	Flavobacterium	teat-sealant	7.80	0.60	17.80
genus	Flavobacterium	control	2.20	0.20	167.20
genus	Fluviicola	cephalonium	4.20	37.80	3.80
genus	Fluviicola	cloxacillin	33.20	110.40	344.80
genus	Fluviicola	teat-sealant	34.20	50.00	737.00
genus	Fluviicola	control	1.00	310.40	600.40
genus	Fructobacillus	cephalonium	0.20	0.00	368.40
genus	Fructobacillus	cloxacillin	0.00	0.20	104.60
genus	Fructobacillus	teat-sealant	0.00	0.00	0.00
genus	Fructobacillus	control	0.00	0.00	0.20
genus	Fusibacter	cephalonium	0.20	0.20	0.00
genus	Fusibacter	cloxacillin	8.40	0.20	0.00
genus	Fusibacter	teat-sealant	0.20	0.00	0.20
genus	Fusibacter	control	4.20	0.00	1.80

genus	Fusobacterium	cephalonium	9.60	0.20	86.00
genus	Fusobacterium	cloxacillin	6.60	0.00	7.20
genus	Fusobacterium	teat-sealant	26.00	2.00	98.40
genus	Fusobacterium	control	0.40	50.40	7.60
genus	Gallicola	cephalonium	2.40	0.00	0.00
genus	Gallicola	cloxacillin	19.40	0.00	0.00
genus	Gallicola	teat-sealant	0.00	0.00	0.00
genus	Gallicola	control	0.20	0.00	0.00
genus	Gemmata	cephalonium	97.20	117.80	85.20
genus	Gemmata	cloxacillin	15.80	50.80	273.40
genus	Gemmata	teat-sealant	28.60	166.80	68.60
genus	Gemmata	control	370.80	127.40	3.60
genus	Geodermatophilus	cephalonium	0.00	0.00	0.00
genus	Geodermatophilus	cloxacillin	0.00	5.60	0.40
genus	Geodermatophilus	teat-sealant	0.00	0.00	0.00
genus	Geodermatophilus	control	0.00	0.00	0.00
genus	Georgenia	cephalonium	6.60	40.00	7.20
genus	Georgenia	cloxacillin	15.20	26.80	24.00
genus	Georgenia	teat-sealant	14.00	0.00	60.60
genus	Georgenia	control	21.60	1.40	0.00
genus	Gordonia	cephalonium	2.40	0.00	2.80
genus	Gordonia	cloxacillin	2.00	0.00	1.20
genus	Gordonia	teat-sealant	1.80	0.00	4.80
genus	Gordonia	control	0.00	0.20	0.00
genus	Gracilibacillus	cephalonium	1.80	13.20	0.00
genus	Gracilibacillus	cloxacillin	0.00	0.60	23.60
genus	Gracilibacillus	teat-sealant	0.00	0.00	0.00
genus	Gracilibacillus	control	0.00	8.00	0.00
genus	Granulicatella	cephalonium	0.00	1.80	0.00
genus	Granulicatella	cloxacillin	0.00	6.00	108.20
genus	Granulicatella	teat-sealant	0.40	0.00	5.80
genus	Granulicatella	control	22.60	0.00	17.80
genus	Grevillea	cephalonium	0.00	0.20	0.00
genus	Grevillea	cloxacillin	0.00	0.00	0.60
genus	Grevillea	teat-sealant	0.00	0.40	0.40
genus	Grevillea	control	0.00	0.00	0.40
genus	Guggenheimella	cephalonium	39.40	193.20	0.00
genus	Guggenheimella	cloxacillin	7.60	0.00	0.00
genus	Guggenheimella	teat-sealant	5.40	18.60	17.00
genus	Guggenheimella	control	4.40	0.00	0.00
genus	GW-34	cephalonium	9.40	0.00	0.00
genus	GW-34	cloxacillin	32.80	0.00	0.00
genus	GW-34	teat-sealant	40.40	0.00	3.00
genus	GW-34	control	22.20	0.00	0.20
genus	Haemophilus	cephalonium	0.00	21.80	0.00

genus	Haemophilus	cloxacillin	4.60	13.40	24.60
genus	Haemophilus	teat-sealant	57.40	2.40	140.80
genus	Haemophilus	control	164.40	0.60	60.20
genus	Hafnia	cephalonium	0.20	41.20	5.20
genus	Hafnia	cloxacillin	0.00	0.00	0.00
genus	Hafnia	teat-sealant	2.60	0.00	0.00
genus	Hafnia	control	0.00	0.20	0.40
genus	Halomonas	cephalonium	31.00	433.00	27.60
genus	Halomonas	cloxacillin	18.00	210.20	14.40
genus	Halomonas	teat-sealant	2.80	56.40	0.40
genus	Halomonas	control	13.40	25.20	0.00
genus	Helcococcus	cephalonium	17.00	0.00	0.00
genus	Helcococcus	cloxacillin	7.00	0.00	0.00
genus	Helcococcus	teat-sealant	0.00	0.00	0.00
genus	Helcococcus	control	35.00	0.20	0.00
genus	Herminiimonas	cephalonium	0.80	0.60	1.80
genus	Herminiimonas	cloxacillin	0.00	0.00	1.60
genus	Herminiimonas	teat-sealant	1.40	2.40	0.60
genus	Herminiimonas	control	0.40	0.40	0.00
genus	HTCC	cephalonium	0.60	3.00	0.00
genus	HTCC	cloxacillin	0.00	0.00	0.00
genus	HTCC	teat-sealant	0.00	0.00	0.00
genus	HTCC	control	0.20	0.00	0.00
genus	Hydrogenophaga	cephalonium	14.00	0.80	92.40
genus	Hydrogenophaga	cloxacillin	0.00	0.00	69.60
genus	Hydrogenophaga	teat-sealant	0.00	0.80	0.00
genus	Hydrogenophaga	control	0.00	0.80	89.60
genus	Hymenobacter	cephalonium	180.00	80.00	42.80
genus	Hymenobacter	cloxacillin	1720.40	83.80	0.80
genus	Hymenobacter	teat-sealant	19.00	82.20	3.20
genus	Hymenobacter	control	75.00	0.20	13.40
genus	Hyphomicrobium	cephalonium	0.00	21.20	13.20
genus	Hyphomicrobium	cloxacillin	0.00	0.00	15.20
genus	Hyphomicrobium	teat-sealant	1.40	5.40	16.40
genus	Hyphomicrobium	control	6.60	7.40	9.40
genus	Ignatzschineria	cephalonium	0.60	2.80	0.00
genus	Ignatzschineria	cloxacillin	0.00	0.00	0.00
genus	Ignatzschineria	teat-sealant	0.00	0.00	0.20
genus	Ignatzschineria	control	0.00	0.00	11.20
genus	Janibacter	cephalonium	22.60	27.00	4.60
genus	Janibacter	cloxacillin	23.60	24.80	40.40
genus	Janibacter	teat-sealant	44.40	0.60	210.00
genus	Janibacter	control	16.20	4.60	0.20
genus	Janthinobacterium	cephalonium	0.60	0.40	65.20
genus	Janthinobacterium	cloxacillin	0.80	10.60	4.00

genus	Janthinobacterium	teat-sealant	2.20	11.40	2.40
genus	Janthinobacterium	control	0.60	0.00	51.60
genus	Jeotgalicoccus	cephalonium	308.00	319.80	256.40
genus	Jeotgalicoccus	cloxacillin	533.00	102.00	172.40
genus	Jeotgalicoccus	teat-sealant	111.80	33.80	600.40
genus	Jeotgalicoccus	control	271.40	246.60	124.60
genus	Jonesia	cephalonium	4.60	33.80	0.60
genus	Jonesia	cloxacillin	11.80	0.00	0.00
genus	Jonesia	teat-sealant	0.00	0.00	0.00
genus	Jonesia	control	5.00	0.00	0.20
genus	Kaistibacter	cephalonium	2.00	8.40	0.40
genus	Kaistibacter	cloxacillin	0.00	1.40	6.80
genus	Kaistibacter	teat-sealant	0.60	6.00	3.00
genus	Kaistibacter	control	2.20	2.20	0.80
genus	Kaistobacter	cephalonium	33.00	40.80	18.40
genus	Kaistobacter	cloxacillin	77.40	25.40	17.80
genus	Kaistobacter	teat-sealant	10.80	10.60	0.60
genus	Kaistobacter	control	6.00	16.60	17.40
genus	Kerstersia	cephalonium	0.00	0.40	0.00
genus	Kerstersia	cloxacillin	0.00	0.00	0.00
genus	Kerstersia	teat-sealant	0.00	0.00	0.00
genus	Kerstersia	control	0.80	0.20	0.60
genus	Kineococcus	cephalonium	0.60	12.00	0.00
genus	Kineococcus	cloxacillin	0.00	9.00	5.80
genus	Kineococcus	teat-sealant	2.80	0.00	0.00
genus	Kineococcus	control	0.00	0.00	0.00
genus	Kineosphaera	cephalonium	0.60	0.00	5.20
genus	Kineosphaera	cloxacillin	0.00	0.00	0.00
genus	Kineosphaera	teat-sealant	0.00	0.00	0.00
genus	Kineosphaera	control	0.80	0.00	0.00
genus	Kingella	cephalonium	0.00	3.20	0.00
genus	Kingella	cloxacillin	0.00	0.00	0.00
genus	Kingella	teat-sealant	0.00	71.60	0.00
genus	Kingella	control	11.00	16.80	15.80
genus	Klebsiella	cephalonium	0.00	13.80	28.40
genus	Klebsiella	cloxacillin	0.00	57.60	5.20
genus	Klebsiella	teat-sealant	0.00	34.00	1.60
genus	Klebsiella	control	3.60	15955.00	13.20
genus	Knoellia	cephalonium	0.60	0.40	0.40
genus	Knoellia	cloxacillin	2.20	0.00	0.40
genus	Knoellia	teat-sealant	2.00	0.00	3.00
genus	Knoellia	control	0.80	0.00	0.20
genus	Kocuria	cephalonium	29.00	133.40	0.80
genus	Kocuria	cloxacillin	81.20	20.80	39.00
genus	Kocuria	teat-sealant	46.80	100.20	46.00

genus	Kocuria	control	18.60	5.60	61.60
genus	Kurthia	cephalonium	0.20	0.00	0.00
genus	Kurthia	cloxacillin	1.80	0.00	0.00
genus	Kurthia	teat-sealant	0.60	0.00	0.00
genus	Kurthia	control	0.20	0.00	0.00
genus	Kytococcus	cephalonium	0.00	0.00	0.00
genus	Kytococcus	cloxacillin	0.00	0.00	0.40
genus	Kytococcus	teat-sealant	0.00	0.00	3.00
genus	Kytococcus	control	1.40	0.00	0.00
genus	L7A_E11	cephalonium	0.00	0.00	0.00
genus	L7A_E11	cloxacillin	26.20	0.20	0.00
genus	L7A_E11	teat-sealant	0.00	0.00	0.00
genus	L7A_E11	control	0.00	0.00	0.00
genus	Labrys	cephalonium	0.00	0.00	0.00
genus	Labrys	cloxacillin	0.00	0.00	0.00
genus	Labrys	teat-sealant	0.00	4.20	0.20
genus	Labrys	control	0.00	14.40	0.00
genus	Lachnospira	cephalonium	0.80	8.00	0.00
genus	Lachnospira	cloxacillin	5.00	0.00	0.00
genus	Lachnospira	teat-sealant	0.00	0.00	0.00
genus	Lachnospira	control	2.00	0.00	0.00
genus	Lactobacillus	cephalonium	2501.60	4017.40	6981.80
genus	Lactobacillus	cloxacillin	2450.40	3737.20	4272.00
genus	Lactobacillus	teat-sealant	3150.80	4929.20	7995.80
genus	Lactobacillus	control	2520.80	4574.40	7258.00
genus	Lactococcus	cephalonium	86.20	120.00	488.20
genus	Lactococcus	cloxacillin	44.80	546.40	476.00
genus	Lactococcus	teat-sealant	16.20	417.20	296.80
genus	Lactococcus	control	490.80	179.00	365.80
genus	Leptothrix	cephalonium	0.20	0.20	0.00
genus	Leptothrix	cloxacillin	0.00	0.20	0.20
genus	Leptothrix	teat-sealant	0.00	0.60	0.20
genus	Leptothrix	control	0.00	0.40	0.00
genus	Leptotrichia	cephalonium	0.20	126.20	0.00
genus	Leptotrichia	cloxacillin	0.00	0.00	0.00
genus	Leptotrichia	teat-sealant	18.40	0.00	6.00
genus	Leptotrichia	control	0.00	0.00	0.00
genus	Leucobacter	cephalonium	21.60	138.80	14.20
genus	Leucobacter	cloxacillin	45.20	41.60	22.20
genus	Leucobacter	teat-sealant	57.80	3.00	13.40
genus	Leucobacter	control	49.00	21.60	2.00
genus	Leuconostoc	cephalonium	0.00	243.80	147.60
genus	Leuconostoc	cloxacillin	30.80	131.40	141.20
genus	Leuconostoc	teat-sealant	0.20	0.00	233.60
genus	Leuconostoc	control	0.40	28.00	0.20

genus	Limnohabitans	cephalonium	0.00	0.00	35.20
genus	Limnohabitans	cloxacillin	1.60	0.00	1.40
genus	Limnohabitans	teat-sealant	0.00	0.00	0.00
genus	Limnohabitans	control	0.20	8.80	69.20
genus	Luteimicrobium	cephalonium	3.80	0.80	1.60
genus	Luteimicrobium	cloxacillin	36.80	1.20	24.00
genus	Luteimicrobium	teat-sealant	0.00	0.20	0.40
genus	Luteimicrobium	control	7.00	0.20	135.80
genus	Luteimonas	cephalonium	88.60	443.00	39.40
genus	Luteimonas	cloxacillin	117.40	140.80	322.20
genus	Luteimonas	teat-sealant	71.80	0.40	96.60
genus	Luteimonas	control	59.80	66.80	0.20
genus	Luteococcus	cephalonium	3.00	0.00	0.00
genus	Luteococcus	cloxacillin	0.00	0.00	0.00
genus	Luteococcus	teat-sealant	0.00	0.00	4.60
genus	Luteococcus	control	0.40	0.00	0.00
genus	Luteolibacter	cephalonium	0.00	6.40	0.00
genus	Luteolibacter	cloxacillin	0.00	128.20	0.00
genus	Luteolibacter	teat-sealant	0.40	595.20	0.40
genus	Luteolibacter	control	0.00	7.60	124.80
genus	Lutibacterium	cephalonium	2.60	57.00	0.60
genus	Lutibacterium	cloxacillin	5.60	1.60	3.20
genus	Lutibacterium	teat-sealant	0.40	0.00	2.40
genus	Lutibacterium	control	0.60	0.20	2.20
genus	Lyngbya	cephalonium	0.00	0.80	0.00
genus	Lyngbya	cloxacillin	0.00	1.20	0.00
genus	Lyngbya	teat-sealant	0.00	0.40	0.00
genus	Lyngbya	control	0.00	0.00	0.00
genus	Lysinibacillus	cephalonium	14.80	99.60	25.40
genus	Lysinibacillus	cloxacillin	58.60	28.00	4.80
genus	Lysinibacillus	teat-sealant	22.20	0.00	1.60
genus	Lysinibacillus	control	1.20	4.60	0.00
genus	Lysobacter	cephalonium	0.00	0.00	0.00
genus	Lysobacter	cloxacillin	26.60	222.80	0.60
genus	Lysobacter	teat-sealant	0.00	0.00	0.00
genus	Lysobacter	control	0.40	0.00	0.00
genus	Macrococcus	cephalonium	0.00	0.00	0.00
genus	Macrococcus	cloxacillin	31.00	0.00	0.00
genus	Macrococcus	teat-sealant	0.00	27.40	0.20
genus	Macrococcus	control	0.00	0.20	0.00
genus	Marinibacillus	cephalonium	1.60	11.60	0.40
genus	Marinibacillus	cloxacillin	1.40	5.80	3.40
genus	Marinibacillus	teat-sealant	2.60	0.00	7.20
genus	Marinibacillus	control	1.20	0.20	0.40
genus	Marinobacter	cephalonium	5.00	1.80	0.00

genus	Marinobacter	cloxacillin	0.80	0.40	0.00
genus	Marinobacter	teat-sealant	0.00	0.00	0.00
genus	Marinobacter	control	0.00	0.00	0.00
genus	Megasphaera	cephalonium	0.00	0.00	0.00
genus	Megasphaera	cloxacillin	19.00	0.20	0.00
genus	Megasphaera	teat-sealant	0.00	1.20	0.00
genus	Megasphaera	control	0.00	0.00	0.00
genus	Mesorhizobium	cephalonium	1.40	0.00	9.40
genus	Mesorhizobium	cloxacillin	1.40	15.80	26.20
genus	Mesorhizobium	teat-sealant	3.80	3.80	15.60
genus	Mesorhizobium	control	9.00	12.00	6.20
genus	Methanobrevibacter	cephalonium	222.20	43.20	7.60
genus	Methanobrevibacter	cloxacillin	246.20	337.40	45.00
genus	Methanobrevibacter	teat-sealant	103.00	35.40	44.60
genus	Methanobrevibacter	control	123.00	43.80	23.40
genus	Methanosarcina	cephalonium	0.00	0.00	0.00
genus	Methanosarcina	cloxacillin	1.40	0.00	0.00
genus	Methanosarcina	teat-sealant	0.00	0.00	0.00
genus	Methanosarcina	control	2.00	0.00	0.00
genus	Methanosphaera	cephalonium	26.80	0.00	12.80
genus	Methanosphaera	cloxacillin	193.20	0.40	0.00
genus	Methanosphaera	teat-sealant	0.00	0.00	0.00
genus	Methanosphaera	control	7.20	0.00	0.00
genus	Methylobacterium	cephalonium	104.40	136.60	30.00
genus	Methylobacterium	cloxacillin	209.60	92.20	190.20
genus	Methylobacterium	teat-sealant	71.60	164.60	484.60
genus	Methylobacterium	control	55.60	53.20	262.20
genus	Methylopila	cephalonium	0.00	0.00	0.00
genus	Methylopila	cloxacillin	0.00	0.00	0.00
genus	Methylopila	teat-sealant	0.00	0.00	0.00
genus	Methylopila	control	0.20	5.00	0.00
genus	Methyloversatilis	cephalonium	0.00	0.00	0.00
genus	Methyloversatilis	cloxacillin	0.00	0.00	0.00
genus	Methyloversatilis	teat-sealant	0.00	8.20	4.40
genus	Methyloversatilis	control	0.00	0.00	0.00
genus	Microbacterium	cephalonium	67.20	435.20	148.60
genus	Microbacterium	cloxacillin	174.80	91.00	68.60
genus	Microbacterium	teat-sealant	56.40	42.00	99.40
genus	Microbacterium	control	22.60	44.40	60.60
genus	Microbispora	cephalonium	1.00	6.80	1.60
genus	Microbispora	cloxacillin	0.60	0.00	1.80
genus	Microbispora	teat-sealant	1.60	0.60	0.60
genus	Microbispora	control	0.40	0.40	0.60
genus	Micrococcus	cephalonium	35.40	77.40	99.40
genus	Micrococcus	cloxacillin	107.60	21.00	46.80

genus	Micrococcus	teat-sealant	25.00	39.00	31.80
genus	Micrococcus	control	38.40	22.80	27.20
genus	Microlunatus	cephalonium	11.40	0.60	0.20
genus	Microlunatus	cloxacillin	0.00	0.00	0.00
genus	Microlunatus	teat-sealant	0.00	0.00	0.00
genus	Microlunatus	control	39.80	0.00	0.00
genus	Mitsuokella	cephalonium	0.00	0.20	0.00
genus	Mitsuokella	cloxacillin	18.20	0.00	0.00
genus	Mitsuokella	teat-sealant	0.00	0.00	0.00
genus	Mitsuokella	control	7.60	0.00	0.00
genus	Modestobacter	cephalonium	0.00	0.00	0.00
genus	Modestobacter	cloxacillin	0.00	51.20	14.20
genus	Modestobacter	teat-sealant	0.00	0.00	0.20
genus	Modestobacter	control	0.00	2.80	0.00
genus	Mogibacterium	cephalonium	10.20	6.80	0.20
genus	Mogibacterium	cloxacillin	35.40	5.20	0.20
genus	Mogibacterium	teat-sealant	4.60	0.00	2.20
genus	Mogibacterium	control	5.40	4.80	0.00
genus	Moraxella	cephalonium	20.60	0.00	0.20
genus	Moraxella	cloxacillin	0.00	0.00	28.20
genus	Moraxella	teat-sealant	0.00	16.20	0.00
genus	Moraxella	control	5.60	0.40	28.00
genus	Morganella	cephalonium	0.00	0.00	0.00
genus	Morganella	cloxacillin	0.00	0.00	0.00
genus	Morganella	teat-sealant	0.00	0.00	1.00
genus	Morganella	control	1.20	0.00	82.00
genus	Muricauda	cephalonium	0.40	0.00	0.00
genus	Muricauda	cloxacillin	0.60	0.00	0.00
genus	Muricauda	teat-sealant	1.20	0.00	0.00
genus	Muricauda	control	1.80	0.00	0.00
genus	Mycetocola	cephalonium	55.00	194.20	98.00
genus	Mycetocola	cloxacillin	65.60	68.00	88.20
genus	Mycetocola	teat-sealant	36.40	82.40	67.60
genus	Mycetocola	control	47.60	13.20	504.00
genus	Mycobacterium	cephalonium	7.20	2.20	4.40
genus	Mycobacterium	cloxacillin	0.00	0.00	59.40
genus	Mycobacterium	teat-sealant	1.80	26.00	30.20
genus	Mycobacterium	control	0.00	26.00	0.00
genus	Mycoplana	cephalonium	0.00	0.00	27.60
genus	Mycoplana	cloxacillin	0.00	0.00	0.00
genus	Mycoplana	teat-sealant	0.00	0.00	0.00
genus	Mycoplana	control	0.00	4.40	12.20
genus	Myroides	cephalonium	7.40	0.80	89.60
genus	Myroides	cloxacillin	0.20	0.00	5.80
genus	Myroides	teat-sealant	0.40	0.20	246.00

genus	Myroides	control	0.00	0.40	16.20
genus	Natronobacillus	cephalonium	91.00	63.20	59.20
genus	Natronobacillus	cloxacillin	109.20	117.60	72.60
genus	Natronobacillus	teat-sealant	13.20	0.00	0.00
genus	Natronobacillus	control	122.00	15.40	43.40
genus	Neisseria	cephalonium	0.00	0.00	0.00
genus	Neisseria	cloxacillin	0.00	5.80	35.20
genus	Neisseria	teat-sealant	77.80	14.20	43.00
genus	Neisseria	control	0.00	0.00	0.00
genus	Nelumbo	cephalonium	2.20	136.00	66.80
genus	Nelumbo	cloxacillin	19.00	70.00	113.60
genus	Nelumbo	teat-sealant	4.20	42.40	35.20
genus	Nelumbo	control	2.00	16.20	175.80
genus	Nesterenkonia	cephalonium	2.40	0.20	2.00
genus	Nesterenkonia	cloxacillin	41.80	0.60	15.40
genus	Nesterenkonia	teat-sealant	0.00	0.00	18.20
genus	Nesterenkonia	control	0.20	0.00	0.00
genus	Nitratireductor	cephalonium	5.80	0.80	2.80
genus	Nitratireductor	cloxacillin	1.40	0.80	0.00
genus	Nitratireductor	teat-sealant	0.00	1.80	6.00
genus	Nitratireductor	control	0.00	0.40	4.40
genus	Nitrobacter	cephalonium	0.40	0.00	0.20
genus	Nitrobacter	cloxacillin	0.40	0.00	0.00
genus	Nitrobacter	teat-sealant	0.20	0.60	0.00
genus	Nitrobacter	control	0.40	0.00	0.00
genus	Nocardioides	cephalonium	0.00	2.00	0.00
genus	Nocardioides	cloxacillin	1.00	0.40	0.00
genus	Nocardioides	teat-sealant	0.20	0.00	0.00
genus	Nocardioides	control	0.00	0.00	0.00
genus	Novosphingobium	cephalonium	15.80	203.80	13.20
genus	Novosphingobium	cloxacillin	16.00	125.80	119.20
genus	Novosphingobium	teat-sealant	3.40	52.80	219.60
genus	Novosphingobium	control	46.00	122.60	138.60
genus	Oceanobacillus	cephalonium	0.00	0.00	0.00
genus	Oceanobacillus	cloxacillin	0.00	0.20	0.00
genus	Oceanobacillus	teat-sealant	0.00	0.00	2.20
genus	Oceanobacillus	control	0.00	0.00	0.00
genus	Ochrobactrum	cephalonium	1.20	29.00	29.20
genus	Ochrobactrum	cloxacillin	1.80	5.60	5.60
genus	Ochrobactrum	teat-sealant	3.60	10.00	4.00
genus	Ochrobactrum	control	0.40	0.80	0.40
genus	Odoribacter	cephalonium	0.00	0.00	0.00
genus	Odoribacter	cloxacillin	1.20	18.60	0.00
genus	Odoribacter	teat-sealant	0.00	0.00	0.00
genus	Odoribacter	control	13.60	0.20	0.00

genus	Oligella	cephalonium	185.60	459.20	699.40
genus	Oligella	cloxacillin	115.40	139.00	161.80
genus	Oligella	teat-sealant	19.40	68.00	983.40
genus	Oligella	control	39.20	42.60	76.80
genus	Ornithinibacillus	cephalonium	0.40	2.40	0.40
genus	Ornithinibacillus	cloxacillin	1.40	1.40	0.00
genus	Ornithinibacillus	teat-sealant	2.60	0.00	16.60
genus	Ornithinibacillus	control	1.20	0.00	0.00
genus	Oscillospira	cephalonium	292.60	89.20	138.60
genus	Oscillospira	cloxacillin	479.00	23.20	33.60
genus	Oscillospira	teat-sealant	135.40	155.80	70.00
genus	Oscillospira	control	113.40	46.80	16.80
genus	p-75-a5	cephalonium	0.00	0.00	0.00
genus	p-75-a5	cloxacillin	4.40	0.00	0.00
genus	p-75-a5	teat-sealant	0.20	0.00	0.00
genus	p-75-a5	control	0.00	0.00	0.00
genus	Paenibacillus	cephalonium	13.00	0.80	0.00
genus	Paenibacillus	cloxacillin	11.40	27.20	28.20
genus	Paenibacillus	teat-sealant	0.00	0.00	0.60
genus	Paenibacillus	control	15.80	0.00	0.00
genus	Paenochrobactrum	cephalonium	6.40	1.60	60.40
genus	Paenochrobactrum	cloxacillin	2.60	0.80	11.40
genus	Paenochrobactrum	teat-sealant	0.00	0.20	3.40
genus	Paenochrobactrum	control	0.00	0.40	0.20
genus	Palleronia	cephalonium	1.00	4.40	0.00
genus	Palleronia	cloxacillin	0.60	0.40	6.00
genus	Palleronia	teat-sealant	0.00	0.00	1.00
genus	Palleronia	control	0.80	0.40	0.00
genus	Paludibacter	cephalonium	26.80	0.20	0.00
genus	Paludibacter	cloxacillin	19.00	0.00	0.00
genus	Paludibacter	teat-sealant	5.40	0.00	19.80
genus	Paludibacter	control	18.00	6.40	0.20
genus	Paracoccus	cephalonium	54.00	99.20	112.40
genus	Paracoccus	cloxacillin	187.00	89.60	145.40
genus	Paracoccus	teat-sealant	11.80	162.80	318.80
genus	Paracoccus	control	91.40	46.20	135.80
genus	Parvibaculum	cephalonium	0.00	0.00	0.00
genus	Parvibaculum	cloxacillin	0.00	0.40	0.00
genus	Parvibaculum	teat-sealant	0.00	1.00	0.00
genus	Parvibaculum	control	0.00	1.20	0.00
genus	Parvimonas	cephalonium	2.00	0.00	3.40
genus	Parvimonas	cloxacillin	12.00	0.00	0.00
genus	Parvimonas	teat-sealant	0.00	0.00	0.00
genus	Parvimonas	control	0.00	0.00	0.00
genus	Pediococcus	cephalonium	104.60	155.00	366.20

genus	Pediococcus	cloxacillin	138.20	300.00	771.80
genus	Pediococcus	teat-sealant	87.00	731.60	130.20
genus	Pediococcus	control	214.20	135.60	332.40
genus	Pedobacter	cephalonium	0.00	97.40	5.00
genus	Pedobacter	cloxacillin	22.40	0.40	7.60
genus	Pedobacter	teat-sealant	141.80	1.00	0.00
genus	Pedobacter	control	1.20	1.40	23.80
genus	Pedomicrobium	cephalonium	0.00	0.00	0.00
genus	Pedomicrobium	cloxacillin	0.00	0.00	0.00
genus	Pedomicrobium	teat-sealant	0.00	14.60	0.00
genus	Pedomicrobium	control	0.00	0.00	0.00
genus	Peptococcus	cephalonium	0.00	0.00	0.00
genus	Peptococcus	cloxacillin	0.00	0.00	0.00
genus	Peptococcus	teat-sealant	0.00	0.20	19.80
genus	Peptococcus	control	0.00	0.00	0.00
genus	Peptoniphilus	cephalonium	0.00	0.20	3.60
genus	Peptoniphilus	cloxacillin	16.60	4.20	69.60
genus	Peptoniphilus	teat-sealant	0.00	4.80	0.00
genus	Peptoniphilus	control	0.00	0.00	0.00
genus	Peptostreptococcus	cephalonium	3.40	0.00	0.00
genus	Peptostreptococcus	cloxacillin	0.00	0.00	0.00
genus	Peptostreptococcus	teat-sealant	0.00	0.00	0.00
genus	Peptostreptococcus	control	0.00	0.00	2.40
genus	Perlucidibaca	cephalonium	0.00	0.00	0.00
genus	Perlucidibaca	cloxacillin	1.00	0.00	18.20
genus	Perlucidibaca	teat-sealant	16.80	0.00	0.00
genus	Perlucidibaca	control	0.00	0.00	0.00
genus	ph2	cephalonium	5.60	0.00	0.00
genus	ph2	cloxacillin	9.60	0.00	0.00
genus	ph2	teat-sealant	1.00	0.00	0.00
genus	ph2	control	11.80	0.00	0.00
genus	Phascolarctobacterium	cephalonium	557.40	428.20	403.20
genus	Phascolarctobacterium	cloxacillin	1612.20	145.00	73.40
genus	Phascolarctobacterium	teat-sealant	697.80	55.00	2.00
genus	Phascolarctobacterium	control	1091.00	525.00	52.80
genus	Phenylobacterium	cephalonium	35.20	54.20	26.20
genus	Phenylobacterium	cloxacillin	10.20	17.60	18.00
genus	Phenylobacterium	teat-sealant	10.00	46.60	27.80
genus	Phenylobacterium	control	29.80	19.00	33.60
genus	Phyllobacterium	cephalonium	0.40	0.40	0.00
genus	Phyllobacterium	cloxacillin	0.00	0.00	0.00
genus	Phyllobacterium	teat-sealant	0.40	0.00	0.80
genus	Phyllobacterium	control	0.20	0.40	0.20
genus	Pigmentiphaga	cephalonium	0.00	0.00	0.00
genus	Pigmentiphaga	cloxacillin	0.00	0.00	0.20

genus	Pigmentiphaga	teat-sealant	0.20	40.40	0.00
genus	Pigmentiphaga	control	0.20	0.00	0.00
genus	Pilimelia	cephalonium	0.20	0.00	0.00
genus	Pilimelia	cloxacillin	1.20	2.00	0.00
genus	Pilimelia	teat-sealant	0.00	0.00	0.00
genus	Pilimelia	control	7.20	0.00	0.00
genus	Pirellula	cephalonium	0.00	0.00	0.00
genus	Pirellula	cloxacillin	0.20	21.00	0.00
genus	Pirellula	teat-sealant	0.00	0.00	0.00
genus	Pirellula	control	0.00	0.00	0.00
genus	Planctomyces	cephalonium	0.20	0.60	251.40
genus	Planctomyces	cloxacillin	0.00	0.20	17.20
genus	Planctomyces	teat-sealant	0.00	0.00	0.00
genus	Planctomyces	control	0.00	0.00	0.00
genus	Planococcus	cephalonium	0.40	0.00	0.00
genus	Planococcus	cloxacillin	1.00	0.00	0.00
genus	Planococcus	teat-sealant	4.40	0.00	12.00
genus	Planococcus	control	2.20	0.00	0.00
genus	Planomicrobium	cephalonium	9.60	35.80	7.00
genus	Planomicrobium	cloxacillin	30.20	5.00	12.80
genus	Planomicrobium	teat-sealant	47.00	14.40	97.00
genus	Planomicrobium	control	35.80	0.20	0.00
genus	Polaromonas	cephalonium	0.00	0.00	40.80
genus	Polaromonas	cloxacillin	0.00	0.00	0.00
genus	Polaromonas	teat-sealant	0.00	0.00	0.00
genus	Polaromonas	control	0.00	9.20	78.00
genus	Polynucleobacter	cephalonium	21.00	59.40	334.80
genus	Polynucleobacter	cloxacillin	0.00	0.20	94.60
genus	Polynucleobacter	teat-sealant	0.00	0.00	0.00
genus	Polynucleobacter	control	0.00	0.20	39.60
genus	Pontibacter	cephalonium	3.20	0.00	0.00
genus	Pontibacter	cloxacillin	2.20	0.20	0.00
genus	Pontibacter	teat-sealant	0.00	0.00	0.00
genus	Pontibacter	control	0.00	0.00	0.00
genus	Porphyromonas	cephalonium	0.00	0.00	59.80
genus	Porphyromonas	cloxacillin	8.60	13.20	20.60
genus	Porphyromonas	teat-sealant	31.00	0.00	0.40
genus	Porphyromonas	control	21.60	0.00	0.40
genus	Prauserella	cephalonium	32.60	102.80	3.80
genus	Prauserella	cloxacillin	9.80	0.00	3.60
genus	Prauserella	teat-sealant	7.20	0.00	52.20
genus	Prauserella	control	7.80	0.00	1.60
genus	Prevotella	cephalonium	59.80	95.60	154.60
genus	Prevotella	cloxacillin	136.80	20.40	51.20
genus	Prevotella	teat-sealant	13.00	3.20	37.60

genus	Prevotella	control	204.20	17.80	0.80
genus	[Prevotella]	cephalonium	68.40	5.80	56.80
genus	[Prevotella]	cloxacillin	95.80	27.80	1.80
genus	[Prevotella]	teat-sealant	7.20	0.00	2.00
genus	[Prevotella]	control	55.60	18.20	7.40
genus	Propionibacterium	cephalonium	4356.60	8271.80	4278.00
genus	Propionibacterium	cloxacillin	3132.80	5275.60	6210.60
genus	Propionibacterium	teat-sealant	5301.20	9198.60	8233.80
genus	Propionibacterium	control	6878.20	6845.40	5831.40
genus	Propionicimonas	cephalonium	25.60	0.40	0.20
genus	Propionicimonas	cloxacillin	84.00	3.60	0.00
genus	Propionicimonas	teat-sealant	1.80	0.00	0.00
genus	Propionicimonas	control	42.80	0.00	0.60
genus	Prosthecobacter	cephalonium	0.00	0.00	0.00
genus	Prosthecobacter	cloxacillin	0.00	0.00	0.00
genus	Prosthecobacter	teat-sealant	0.00	0.00	0.00
genus	Prosthecobacter	control	0.00	43.60	21.80
genus	Proteiniclasticum	cephalonium	33.40	0.60	6.20
genus	Proteiniclasticum	cloxacillin	53.60	25.60	2.80
genus	Proteiniclasticum	teat-sealant	15.20	0.40	7.20
genus	Proteiniclasticum	control	5.20	0.20	0.20
genus	Providencia	cephalonium	0.00	1.20	3.40
genus	Providencia	cloxacillin	0.00	0.00	0.00
genus	Providencia	teat-sealant	0.00	0.00	0.00
genus	Providencia	control	0.00	0.00	2.80
genus	Pseudidiomarina	cephalonium	0.60	1.20	1.80
genus	Pseudidiomarina	cloxacillin	7.00	0.00	0.00
genus	Pseudidiomarina	teat-sealant	1.80	0.00	14.00
genus	Pseudidiomarina	control	3.40	0.00	0.20
genus	Pseudoalteromonas	cephalonium	0.00	0.00	0.00
genus	Pseudoalteromonas	cloxacillin	0.00	0.00	0.00
genus	Pseudoalteromonas	teat-sealant	2.40	0.00	0.20
genus	Pseudoalteromonas	control	1.00	0.00	1.00
genus	Pseudobutyrivibrio	cephalonium	0.60	0.00	1.80
genus	Pseudobutyrivibrio	cloxacillin	1.20	0.00	0.00
genus	Pseudobutyrivibrio	teat-sealant	0.00	0.00	0.00
genus	Pseudobutyrivibrio	control	1.40	0.00	0.00
genus	Pseudoclavibacter	cephalonium	9.60	39.20	14.40
genus	Pseudoclavibacter	cloxacillin	13.80	9.40	3.40
genus	Pseudoclavibacter	teat-sealant	41.80	0.60	0.60
genus	Pseudoclavibacter	control	13.80	5.20	0.20
genus	Pseudofulvimonas	cephalonium	0.00	0.00	0.00
genus	Pseudofulvimonas	cloxacillin	0.00	0.00	0.00
genus	Pseudofulvimonas	teat-sealant	0.40	0.00	45.40
genus	Pseudofulvimonas	control	0.00	0.00	0.00

genus	Pseudomonas	cephalonium	5865.50	8875.60	10910.40
genus	Pseudomonas	cloxacillin	3882.50	6247.20	11096.50
genus	Pseudomonas	teat-sealant	6940.70	10144.00	11790.50
genus	Pseudomonas	control	6926.70	9875.80	10122.60
genus	Pseudonocardia	cephalonium	0.00	15.80	0.20
genus	Pseudonocardia	cloxacillin	16.40	0.00	0.00
genus	Pseudonocardia	teat-sealant	2.20	0.00	196.80
genus	Pseudonocardia	control	0.00	1.20	0.00
genus	Pseudoramibacter_Eubacterium	cephalonium	2.80	0.00	0.00
genus	Pseudoramibacter_Eubacterium	cloxacillin	10.00	0.00	0.00
genus	Pseudoramibacter_Eubacterium	teat-sealant	0.00	0.00	0.00
genus	Pseudoramibacter_Eubacterium	control	0.00	0.00	0.00
genus	Pseudoxanthomonas	cephalonium	0.00	8.00	0.60
genus	Pseudoxanthomonas	cloxacillin	3.00	1.20	1.80
genus	Pseudoxanthomonas	teat-sealant	8.20	0.60	0.60
genus	Pseudoxanthomonas	control	0.20	0.40	0.20
genus	Psychrobacter	cephalonium	7.00	3.60	69.20
genus	Psychrobacter	cloxacillin	107.60	17.80	119.80
genus	Psychrobacter	teat-sealant	48.40	4.20	160.60
genus	Psychrobacter	control	19.40	35.20	80.20
genus	Ramlibacter	cephalonium	0.00	0.00	5.00
genus	Ramlibacter	cloxacillin	0.00	0.00	0.00
genus	Ramlibacter	teat-sealant	0.00	0.00	0.00
genus	Ramlibacter	control	0.00	2.60	15.40
genus	Rarobacter	cephalonium	0.00	0.60	0.40
genus	Rarobacter	cloxacillin	0.20	0.40	0.20
genus	Rarobacter	teat-sealant	0.00	0.00	0.80
genus	Rarobacter	control	0.00	0.40	0.20
genus	Rathayibacter	cephalonium	1.20	0.00	2.60
genus	Rathayibacter	cloxacillin	0.20	83.20	13.20
genus	Rathayibacter	teat-sealant	0.00	0.00	0.00
genus	Rathayibacter	control	0.80	0.00	0.00
genus	rc4-4	cephalonium	36.60	23.00	0.00
genus	rc4-4	cloxacillin	121.40	0.20	0.20
genus	rc4-4	teat-sealant	25.80	0.20	0.00
genus	rc4-4	control	96.60	4.20	0.00
genus	Rheinheimera	cephalonium	0.00	0.00	13.00
genus	Rheinheimera	cloxacillin	6.20	0.00	0.00
genus	Rheinheimera	teat-sealant	1.20	1.20	0.00
genus	Rheinheimera	control	4.20	3.80	0.00
genus	Rhizobium	cephalonium	0.00	0.00	0.00
genus	Rhizobium	cloxacillin	0.00	0.00	0.20
genus	Rhizobium	teat-sealant	5.00	0.00	0.00
genus	Rhizobium	control	0.00	0.00	0.00
genus	Rhodobaca	cephalonium	13.80	32.60	0.80

genus	Rhodobaca	cloxacillin	20.60	2.40	10.40
genus	Rhodobaca	teat-sealant	1.40	1.60	0.00
genus	Rhodobaca	control	3.00	5.40	1.00
genus	Rhodobacter	cephalonium	15.60	316.80	3.60
genus	Rhodobacter	cloxacillin	78.00	49.40	125.40
genus	Rhodobacter	teat-sealant	23.00	72.20	41.40
genus	Rhodobacter	control	13.60	27.40	29.80
genus	Rhodococcus	cephalonium	21.20	9.20	2.20
genus	Rhodococcus	cloxacillin	7.60	8.80	63.00
genus	Rhodococcus	teat-sealant	7.40	1.20	2.40
genus	Rhodococcus	control	2.40	10.20	1.20
genus	Rhodoferax	cephalonium	0.00	0.00	19.80
genus	Rhodoferax	cloxacillin	0.00	0.00	0.00
genus	Rhodoferax	teat-sealant	0.00	0.00	0.00
genus	Rhodoferax	control	0.00	0.00	11.40
genus	Rhodoplanes	cephalonium	1.40	11.20	0.40
genus	Rhodoplanes	cloxacillin	1.60	9.60	3.60
genus	Rhodoplanes	teat-sealant	0.60	35.00	43.40
genus	Rhodoplanes	control	0.80	0.40	50.80
genus	Riemerella	cephalonium	3.40	8.20	0.00
genus	Riemerella	cloxacillin	25.00	0.20	0.20
genus	Riemerella	teat-sealant	1.80	0.00	0.00
genus	Riemerella	control	39.40	0.00	0.00
genus	Roseateles	cephalonium	0.00	3.00	0.00
genus	Roseateles	cloxacillin	0.20	0.20	0.00
genus	Roseateles	teat-sealant	0.20	313.00	1.00
genus	Roseateles	control	0.00	0.00	34.20
genus	Roseburia	cephalonium	36.00	6.80	56.40
genus	Roseburia	cloxacillin	135.20	5.60	0.80
genus	Roseburia	teat-sealant	7.40	0.00	5.60
genus	Roseburia	control	29.00	0.00	0.40
genus	Roseomonas	cephalonium	5.40	0.40	0.80
genus	Roseomonas	cloxacillin	0.00	0.00	0.00
genus	Roseomonas	teat-sealant	0.60	0.00	0.00
genus	Roseomonas	control	0.00	12.20	0.60
genus	Rothia	cephalonium	79.60	41.20	20.40
genus	Rothia	cloxacillin	69.00	98.40	1.20
genus	Rothia	teat-sealant	281.80	11.20	2.60
genus	Rothia	control	18.20	110.20	47.20
genus	Rubellimicrobium	cephalonium	0.00	0.00	0.00
genus	Rubellimicrobium	cloxacillin	0.00	0.00	0.00
genus	Rubellimicrobium	teat-sealant	13.00	0.20	0.00
genus	Rubellimicrobium	control	0.00	0.00	0.00
genus	Rubrivivax	cephalonium	0.00	0.00	0.00
genus	Rubrivivax	cloxacillin	0.20	1.00	0.00

genus	Rubrivivax	teat-sealant	0.00	6.60	0.00
genus	Rubrivivax	control	0.00	0.40	1.20
genus	Ruminobacter	cephalonium	69.80	0.00	0.00
genus	Ruminobacter	cloxacillin	226.60	1.60	20.40
genus	Ruminobacter	teat-sealant	75.60	0.00	0.00
genus	Ruminobacter	control	291.20	0.40	0.20
genus	Ruminococcus	cephalonium	172.00	8.40	6.20
genus	Ruminococcus	cloxacillin	534.20	1.60	22.80
genus	Ruminococcus	teat-sealant	165.80	3.00	5.80
genus	Ruminococcus	control	228.80	187.40	53.00
genus	[Ruminococcus]	cephalonium	35.40	18.40	21.60
genus	[Ruminococcus]	cloxacillin	108.60	5.00	41.00
genus	[Ruminococcus]	teat-sealant	87.40	15.80	12.40
genus	[Ruminococcus]	control	38.00	11.20	5.40
genus	Rummeliibacillus	cephalonium	268.00	177.80	306.20
genus	Rummeliibacillus	cloxacillin	804.80	30.60	190.80
genus	Rummeliibacillus	teat-sealant	265.60	92.20	44.40
genus	Rummeliibacillus	control	144.80	36.80	0.80
genus	Runella	cephalonium	0.00	0.00	0.00
genus	Runella	cloxacillin	0.00	0.00	0.00
genus	Runella	teat-sealant	0.20	72.20	0.00
genus	Runella	control	0.00	0.00	0.00
genus	S1	cephalonium	25.60	131.40	0.00
genus	S1	cloxacillin	0.20	464.80	0.60
genus	S1	teat-sealant	0.20	0.20	108.80
genus	S1	control	0.40	202.80	0.40
genus	Saccharopolyspora	cephalonium	0.00	0.00	41.60
genus	Saccharopolyspora	cloxacillin	5.60	0.00	102.00
genus	Saccharopolyspora	teat-sealant	0.40	515.60	5.20
genus	Saccharopolyspora	control	20.40	0.00	48.80
genus	Salana	cephalonium	1.60	30.40	1.20
genus	Salana	cloxacillin	10.80	11.40	8.20
genus	Salana	teat-sealant	4.20	0.00	4.00
genus	Salana	control	2.00	4.40	0.00
genus	Salinibacterium	cephalonium	19.40	69.00	6.80
genus	Salinibacterium	cloxacillin	8.00	34.40	61.80
genus	Salinibacterium	teat-sealant	23.60	18.40	22.60
genus	Salinibacterium	control	7.80	0.60	5.60
genus	Salinicoccus	cephalonium	39.60	0.00	2.00
genus	Salinicoccus	cloxacillin	35.00	0.00	0.00
genus	Salinicoccus	teat-sealant	40.60	0.00	0.00
genus	Salinicoccus	control	24.40	0.00	0.00
genus	Sanguibacter	cephalonium	12.80	41.20	13.00
genus	Sanguibacter	cloxacillin	36.60	11.00	16.40
genus	Sanguibacter	teat-sealant	13.20	20.20	65.80

genus	Sanguibacter	control	23.40	3.80	46.40
genus	Sarcina	cephalonium	0.00	0.00	0.00
genus	Sarcina	cloxacillin	2.60	0.00	0.00
genus	Sarcina	teat-sealant	0.20	0.00	0.00
genus	Sarcina	control	0.00	0.00	0.20
genus	Schumannella	cephalonium	0.40	0.20	0.00
genus	Schumannella	cloxacillin	0.20	0.40	0.00
genus	Schumannella	teat-sealant	0.00	1.20	0.00
genus	Schumannella	control	0.00	0.00	0.00
genus	Schwartzia	cephalonium	0.00	0.00	0.00
genus	Schwartzia	cloxacillin	106.60	0.40	0.60
genus	Schwartzia	teat-sealant	0.00	0.00	0.00
genus	Schwartzia	control	0.00	0.00	0.00
genus	Sedimentibacter	cephalonium	0.00	0.00	0.00
genus	Sedimentibacter	cloxacillin	0.00	0.00	0.20
genus	Sedimentibacter	teat-sealant	0.60	0.40	434.80
genus	Sedimentibacter	control	0.00	0.00	0.00
genus	Sediminibacterium	cephalonium	62.40	149.40	224.60
genus	Sediminibacterium	cloxacillin	21.00	75.00	179.00
genus	Sediminibacterium	teat-sealant	92.60	620.20	286.80
genus	Sediminibacterium	control	205.20	48.40	407.80
genus	Selenomonas	cephalonium	2.40	0.00	0.00
genus	Selenomonas	cloxacillin	18.40	0.00	0.00
genus	Selenomonas	teat-sealant	0.20	53.00	0.40
genus	Selenomonas	control	0.20	0.00	0.00
genus	Serinibacter	cephalonium	0.00	0.40	0.00
genus	Serinibacter	cloxacillin	0.00	0.20	0.00
genus	Serinibacter	teat-sealant	0.00	0.00	2.20
genus	Serinibacter	control	0.00	0.00	0.00
genus	Serinicoccus	cephalonium	8.60	0.00	0.00
genus	Serinicoccus	cloxacillin	5.40	1.80	0.00
genus	Serinicoccus	teat-sealant	0.40	0.20	10.20
genus	Serinicoccus	control	0.40	4.80	0.00
genus	Serpens	cephalonium	0.60	0.40	0.00
genus	Serpens	cloxacillin	17.20	0.60	0.00
genus	Serpens	teat-sealant	0.60	0.00	0.00
genus	Serpens	control	0.20	0.60	0.00
genus	Serratia	cephalonium	0.40	2.60	0.60
genus	Serratia	cloxacillin	0.00	0.00	6.60
genus	Serratia	teat-sealant	0.20	0.00	0.80
genus	Serratia	control	0.00	180.60	47.40
genus	Sharpea	cephalonium	0.80	0.00	0.00
genus	Sharpea	cloxacillin	3.00	0.00	0.60
genus	Sharpea	teat-sealant	0.00	0.00	0.00
genus	Sharpea	control	0.00	0.00	0.00

genus	Shewanella	cephalonium	0.40	0.40	0.40
genus	Shewanella	cloxacillin	0.40	7.20	2.40
genus	Shewanella	teat-sealant	0.60	13.20	0.60
genus	Shewanella	control	0.40	145.60	1.00
genus	Shinella	cephalonium	0.40	0.40	0.20
genus	Shinella	cloxacillin	0.80	0.00	0.00
genus	Shinella	teat-sealant	0.20	0.20	0.20
genus	Shinella	control	0.00	0.40	0.00
genus	Shuttleworthia	cephalonium	10.40	0.20	2.60
genus	Shuttleworthia	cloxacillin	3.80	0.00	0.00
genus	Shuttleworthia	teat-sealant	1.00	0.00	0.00
genus	Shuttleworthia	control	2.00	0.20	0.00
genus	Simplicispira	cephalonium	4.40	0.00	7.20
genus	Simplicispira	cloxacillin	0.20	0.00	0.00
genus	Simplicispira	teat-sealant	0.00	0.00	0.00
genus	Simplicispira	control	1.00	0.00	2.60
genus	Sinorhizobium	cephalonium	0.20	3.80	6.20
genus	Sinorhizobium	cloxacillin	4.00	0.60	2.20
genus	Sinorhizobium	teat-sealant	0.80	2.20	2.40
genus	Sinorhizobium	control	0.80	0.80	2.40
genus	SMB53	cephalonium	361.60	625.80	134.60
genus	SMB53	cloxacillin	1822.00	220.40	102.60
genus	SMB53	teat-sealant	450.40	373.80	247.80
genus	SMB53	control	439.60	127.80	250.20
genus	Solibacillus	cephalonium	932.80	858.00	436.60
genus	Solibacillus	cloxacillin	1530.20	386.20	496.80
genus	Solibacillus	teat-sealant	521.80	9.80	329.20
genus	Solibacillus	control	499.80	98.80	71.00
genus	Sphingobacterium	cephalonium	249.40	1229.60	332.00
genus	Sphingobacterium	cloxacillin	425.20	387.80	637.00
genus	Sphingobacterium	teat-sealant	77.40	10.40	318.20
genus	Sphingobacterium	control	76.00	284.40	29.60
genus	Sphingobium	cephalonium	42.00	161.20	0.40
genus	Sphingobium	cloxacillin	9.80	5.40	96.20
genus	Sphingobium	teat-sealant	8.00	41.40	33.40
genus	Sphingobium	control	29.00	20.00	6.00
genus	Sphingomonas	cephalonium	102.80	262.00	93.60
genus	Sphingomonas	cloxacillin	199.20	126.40	178.80
genus	Sphingomonas	teat-sealant	76.40	64.80	9.60
genus	Sphingomonas	control	26.40	26.00	70.00
genus	Sphingopyxis	cephalonium	12.20	11.80	0.60
genus	Sphingopyxis	cloxacillin	40.00	6.40	0.00
genus	Sphingopyxis	teat-sealant	10.40	72.20	0.20
genus	Sphingopyxis	control	0.00	39.20	0.20
genus	Spirodela	cephalonium	0.00	0.40	0.20

genus	Spirodela	cloxacillin	0.00	0.20	0.20
genus	Spirodela	teat-sealant	0.00	0.40	0.00
genus	Spirodela	control	0.60	0.00	0.00
genus	Spirosoma	cephalonium	11.20	0.00	0.00
genus	Spirosoma	cloxacillin	95.00	7.00	26.20
genus	Spirosoma	teat-sealant	2.80	0.00	0.00
genus	Spirosoma	control	1.80	0.00	0.00
genus	Sporosarcina	cephalonium	14.80	64.80	65.60
genus	Sporosarcina	cloxacillin	70.60	66.00	60.00
genus	Sporosarcina	teat-sealant	12.40	0.00	65.00
genus	Sporosarcina	control	15.00	0.00	0.00
genus	Staphylococcus	cephalonium	741.10	626.30	772.70
genus	Staphylococcus	cloxacillin	812.90	469.10	1072.30
genus	Staphylococcus	teat-sealant	912.30	276.90	1141.20
genus	Staphylococcus	control	684.50	507.00	1369.40
genus	Stenotrophomonas	cephalonium	30.70	151.20	52.00
genus	Stenotrophomonas	cloxacillin	65.60	57.10	104.30
genus	Stenotrophomonas	teat-sealant	66.50	49.60	24.70
genus	Stenotrophomonas	control	50.90	61.10	53.40
genus	Streptacidiphilus	cephalonium	0.40	4.80	0.00
genus	Streptacidiphilus	cloxacillin	0.20	0.00	0.00
genus	Streptacidiphilus	teat-sealant	0.00	0.60	0.40
genus	Streptacidiphilus	control	0.60	0.00	0.00
genus	Streptococcus	cephalonium	6985.20	14505.20	18325.00
genus	Streptococcus	cloxacillin	5949.60	15755.40	19621.60
genus	Streptococcus	teat-sealant	10093.60	12726.60	18401.80
genus	Streptococcus	control	10753.40	15089.20	15290.40
genus	Streptomyces	cephalonium	46.00	37.20	19.40
genus	Streptomyces	cloxacillin	71.40	18.80	11.40
genus	Streptomyces	teat-sealant	46.00	21.20	101.80
genus	Streptomyces	control	52.80	6.20	25.80
genus	Succiniclasticum	cephalonium	4.60	0.00	0.00
genus	Succiniclasticum	cloxacillin	16.40	0.00	0.00
genus	Succiniclasticum	teat-sealant	0.00	0.00	0.00
genus	Succiniclasticum	control	13.60	0.00	321.00
genus	Succinivibrio	cephalonium	89.20	0.00	56.40
genus	Succinivibrio	cloxacillin	243.00	16.60	0.20
genus	Succinivibrio	teat-sealant	66.40	0.20	17.20
genus	Succinivibrio	control	183.40	0.20	0.20
genus	Sutterella	cephalonium	22.00	28.60	20.00
genus	Sutterella	cloxacillin	48.00	0.00	0.00
genus	Sutterella	teat-sealant	0.40	0.00	0.00
genus	Sutterella	control	15.20	0.00	0.00
genus	Syntrophococcus	cephalonium	1.00	0.00	0.00
genus	Syntrophococcus	cloxacillin	2.40	0.00	0.00

genus	Syntrophococcus	teat-sealant	32.00	0.00	0.00
genus	Syntrophococcus	control	0.00	0.00	0.00
genus	Terribacillus	cephalonium	0.00	2.20	0.00
genus	Terribacillus	cloxacillin	0.00	0.40	0.00
genus	Terribacillus	teat-sealant	0.00	0.00	0.00
genus	Terribacillus	control	0.00	0.00	0.00
genus	Tessaracoccus	cephalonium	6.60	0.00	67.60
genus	Tessaracoccus	cloxacillin	72.00	41.40	3.00
genus	Tessaracoccus	teat-sealant	63.00	0.00	0.00
genus	Tessaracoccus	control	4.80	0.00	0.00
genus	Tetrathiobacter	cephalonium	0.60	0.00	0.00
genus	Tetrathiobacter	cloxacillin	0.00	0.00	0.00
genus	Tetrathiobacter	teat-sealant	0.00	0.00	0.00
genus	Tetrathiobacter	control	0.20	0.20	1.00
genus	Thauera	cephalonium	0.00	0.00	0.00
genus	Thauera	cloxacillin	2.20	0.00	0.00
genus	Thauera	teat-sealant	0.00	0.00	0.00
genus	Thauera	control	0.80	0.00	0.00
genus	Thermoactinomyces	cephalonium	0.00	1.60	4.20
genus	Thermoactinomyces	cloxacillin	2.60	0.00	0.00
genus	Thermoactinomyces	teat-sealant	10.60	0.00	76.40
genus	Thermoactinomyces	control	4.00	0.00	0.00
genus	Thermobifida	cephalonium	0.00	0.00	0.00
genus	Thermobifida	cloxacillin	10.40	0.00	0.00
genus	Thermobifida	teat-sealant	0.00	0.00	0.00
genus	Thermobifida	control	0.00	0.00	0.80
genus	Thermomonas	cephalonium	2.60	1.20	11.20
genus	Thermomonas	cloxacillin	5.60	5.60	16.00
genus	Thermomonas	teat-sealant	0.40	80.20	0.20
genus	Thermomonas	control	0.00	0.00	0.00
genus	Thermus	cephalonium	0.00	0.00	0.00
genus	Thermus	cloxacillin	9.60	0.20	258.40
genus	Thermus	teat-sealant	61.00	4.40	0.00
genus	Thermus	control	0.00	0.00	0.00
genus	Tissierella_Soehngenia	cephalonium	20.40	0.00	0.00
genus	Tissierella_Soehngenia	cloxacillin	20.60	0.00	23.80
genus	Tissierella_Soehngenia	teat-sealant	47.20	27.80	0.00
genus	Tissierella_Soehngenia	control	3.80	0.00	1.20
genus	Trabulsiella	cephalonium	0.00	0.60	0.00
genus	Trabulsiella	cloxacillin	0.00	0.00	0.00
genus	Trabulsiella	teat-sealant	0.00	0.00	0.00
genus	Trabulsiella	control	0.60	56.60	0.20
genus	Treponema	cephalonium	185.60	1.20	543.80
genus	Treponema	cloxacillin	81.20	0.00	74.40
genus	Treponema	teat-sealant	166.20	0.20	0.20

genus	Treponema	control	133.80	0.40	0.00
genus	Trichococcus	cephalonium	176.80	241.40	562.20
genus	Trichococcus	cloxacillin	239.40	14.60	301.20
genus	Trichococcus	teat-sealant	67.00	1.20	260.80
genus	Trichococcus	control	60.00	55.40	33.20
genus	Trueperella	cephalonium	5.20	0.00	28.00
genus	Trueperella	cloxacillin	5.40	0.00	0.00
genus	Trueperella	teat-sealant	11.00	0.20	109.20
genus	Trueperella	control	0.00	0.00	0.00
genus	Turicibacter	cephalonium	329.20	57.60	2.80
genus	Turicibacter	cloxacillin	706.60	4.40	15.40
genus	Turicibacter	teat-sealant	216.00	30.20	2.00
genus	Turicibacter	control	1963.40	147.20	1.60
genus	Vagococcus	cephalonium	4.60	0.20	0.00
genus	Vagococcus	cloxacillin	93.00	0.20	0.00
genus	Vagococcus	teat-sealant	0.00	0.00	0.00
genus	Vagococcus	control	0.00	0.20	0.00
genus	Varibaculum	cephalonium	0.00	0.00	0.00
genus	Varibaculum	cloxacillin	0.00	0.00	8.20
genus	Varibaculum	teat-sealant	0.00	0.00	0.00
genus	Varibaculum	control	0.00	18.60	0.00
genus	Variovorax	cephalonium	23.00	0.00	0.60
genus	Variovorax	cloxacillin	5.60	0.00	0.20
genus	Variovorax	teat-sealant	20.80	0.20	0.00
genus	Variovorax	control	0.00	0.00	6.40
genus	Veillonella	cephalonium	43.40	0.00	0.00
genus	Veillonella	cloxacillin	12.60	209.60	234.40
genus	Veillonella	teat-sealant	53.40	0.00	169.20
genus	Veillonella	control	68.00	645.80	161.60
genus	Vibrio	cephalonium	4.60	0.00	0.00
genus	Vibrio	cloxacillin	1.60	0.00	0.00
genus	Vibrio	teat-sealant	0.00	0.00	0.00
genus	Vibrio	control	0.00	0.00	0.00
genus	Victoria	cephalonium	1.20	32.60	17.60
genus	Victoria	cloxacillin	5.00	11.60	17.80
genus	Victoria	teat-sealant	0.40	9.60	7.60
genus	Victoria	control	0.60	3.40	44.80
genus	Virgibacillus	cephalonium	2.20	9.40	10.60
genus	Virgibacillus	cloxacillin	2.00	12.20	2.20
genus	Virgibacillus	teat-sealant	3.80	0.00	10.60
genus	Virgibacillus	control	1.40	0.40	0.00
genus	Vitreoscilla	cephalonium	0.00	0.00	0.00
genus	Vitreoscilla	cloxacillin	18.20	0.20	0.00
genus	Vitreoscilla	teat-sealant	0.60	3.20	0.00
genus	Vitreoscilla	control	0.00	0.00	0.00

genus	Wautersiella	cephalonium	384.40	946.40	595.60
genus	Wautersiella	cloxacillin	447.00	262.40	1245.60
genus	Wautersiella	teat-sealant	90.40	48.40	392.00
genus	Wautersiella	control	101.40	263.60	267.40
genus	Weeksella	cephalonium	21.60	184.40	313.80
genus	Weeksella	cloxacillin	21.60	28.00	392.60
genus	Weeksella	teat-sealant	6.00	0.00	260.20
genus	Weeksella	control	0.00	17.20	10.80
genus	Weissella	cephalonium	0.00	0.00	0.00
genus	Weissella	cloxacillin	3.40	144.20	0.20
genus	Weissella	teat-sealant	0.00	0.00	4.20
genus	Weissella	control	0.00	1.40	0.00
genus	Williamsia	cephalonium	37.00	0.00	0.20
genus	Williamsia	cloxacillin	0.60	0.00	0.00
genus	Williamsia	teat-sealant	0.20	0.00	0.60
genus	Williamsia	control	0.00	0.00	0.00
genus	Xanthomonas	cephalonium	2.00	67.00	0.20
genus	Xanthomonas	cloxacillin	7.20	1.00	0.20
genus	Xanthomonas	teat-sealant	3.20	0.00	45.20
genus	Xanthomonas	control	9.60	0.00	0.20
genus	Xenorhabdus	cephalonium	0.00	2.60	5.60
genus	Xenorhabdus	cloxacillin	0.00	0.60	0.00
genus	Xenorhabdus	teat-sealant	0.00	0.00	0.00
genus	Xenorhabdus	control	0.00	0.00	4.60
genus	Yaniella	cephalonium	73.80	248.40	66.00
genus	Yaniella	cloxacillin	280.20	153.00	145.40
genus	Yaniella	teat-sealant	32.40	0.40	42.00
genus	Yaniella	control	52.40	10.20	3.20
genus	YRC22	cephalonium	50.20	13.00	4.80
genus	YRC22	cloxacillin	69.20	16.80	0.20
genus	YRC22	teat-sealant	43.20	0.00	5.00
genus	YRC22	control	169.00	4.20	2.20
genus	Zhihengliuella	cephalonium	0.00	0.80	0.00
genus	Zhihengliuella	cloxacillin	0.40	0.00	0.40
genus	Zhihengliuella	teat-sealant	0.20	0.00	0.20
genus	Zhihengliuella	control	0.20	0.00	0.40
genus	Zymomonas	cephalonium	0.00	6.20	0.00
genus	Zymomonas	cloxacillin	0.40	0.20	0.00
genus	Zymomonas	teat-sealant	0.20	0.00	0.00
genus	Zymomonas	control	0.00	0.00	6.40
